# Computational Identification of Potent Multitarget Natural Ligands for Alzheimer’s Disease Therapeutics

**DOI:** 10.1155/sci5/1132636

**Published:** 2025-12-29

**Authors:** Nadia Sharif, Ayesha Bibi, Rakhshinda Sadiq, Iffat Ullah, Abdul Rauf, Muhammad Tayyab Arshad, Shahid Bashir, Hafsa Zamir, Sawaira Gull, Taqwa Anwar, Emmanuel Laryea

**Affiliations:** ^1^ Department of Biotechnology, Women University Mardan, Mardan, 23200, Pakistan; ^2^ Department of Human Nutrition and Dietetics, Women University Mardan, Mardan, 23200, Pakistan; ^3^ Department of Pharmaceutical Chemistry, Faculty of Pharmaceutical Sciences, Prince of Songkla University, Hat Yai, 90110, Songkhla, Thailand, psu.ac.th; ^4^ Faculty of Pharmaceutical Sciences, Drug Delivery System Excellence Center, Prince of Songkla University, Hat Yai, 90110, Songkhla, Thailand, psu.ac.th; ^5^ Faculty of Agro-Industry, Functional Food and Nutrition Program, Center of Excellence in Functional Foods and Gastronomy, Prince of Songkla University, Hat Yai, Songkhla, Thailand, psu.ac.th; ^6^ University Institute of Food Science and Technology, The University of Lahore, Lahore, Pakistan, uol.edu.pk; ^7^ Department of Zoology, Women University Mardan, Mardan, 23200, Pakistan; ^8^ Department of Food Science and Technology, Kwame Nkrumah University of Science and Technology, Kumasi, Ghana, knust.edu.gh

**Keywords:** Alzheimer, amyloid-beta (Aβ), ginkgolide, molecular docking, tau protein

## Abstract

Alzheimer’s disease (AD), a complex neurodegenerative disorder, urgently necessitates a multitarget therapeutic approach. This study presents a novel in silico framework targeting a unique combination of four AD‐relevant proteins—sortilin, clusterin, tau, and amyloid‐beta (Aβ)—not previously explored together in multitarget docking studies. The study leveraged a comprehensive computational strategy integrating ADME (absorption, distribution, metabolism, excretion) and ProTox‐3.0 analyses with AutoDock Vina molecular docking, binding, and bond interaction via SiteMap/CASTp and PLIP, respectively. Fifteen novel natural ligands and three established AD reference drugs (donepezil, memantine, and rivastigmine) were assessed against four key AD proteins: sortilin, clusterin, Aβ peptide, and tau. Pharmacokinetic and toxicity predictions revealed favorable drug‐likeness for many ligands, 4‐tert‐amylphenol, allicin, apigenin, and resveratrol, which exhibited high gastrointestinal absorption but varied in blood–brain barrier (BBB) permeation, solubility, and drug‐likeness. Ligands, such as apigenin, cyanidin, and galantamine, demonstrated favorable oral bioavailability and lead‐likeness. Nevertheless, predicted toxicity profiles revealed potential hepatotoxicity concerns for ligands like 4‐tert‐amylphenol and berberine. Comparison with reference drugs highlighted the importance of optimizing ADME properties and minimizing toxicity. Molecular docking results consistently highlighted ginkgolide with multitarget binding to sortilin (−16.29 kcal/mol), clusterin (−13.98 kcal/mol), and tau (−10.63 kcal/mol). Critical interactions were identified, including binding to the aggregation domain of tau via HIS329. Other promising natural ligands, including ginsenosides, berberine, and apigenin, also exhibited strong multitarget interactions. Ginsenosides were a notable lead, demonstrating key molecular contacts with ILE141 on sortilin and directly targeting the Aβ core at ALA4. Apigenin also showed strong binding to the tau repeat domain at ILE328. Notably, memantine displayed significant binding to both sortilin and Aβ, forming a hydrogen bond with the amyloidogenic ILE5 residue. The study identified several potent multitarget binding capabilities compounds, offering compelling avenues for developing novel, more effective therapeutics for AD.

## 1. Introduction

Alzheimer’s disease (AD), a devastating neurodegenerative disorder, currently afflicts over 55 million individuals globally, a figure projected to more than double by 2050, particularly in rapidly aging populations within low‐ and middle‐income countries [1]. This escalating prevalence constitutes a formidable public health crisis, exacting immense societal and economic tolls worldwide. Pathologically, AD traits include the accumulation of intracellular neurofibrillary tangles of hyperphosphorylated tau protein and extracellular amyloid‐beta (Aβ) plaques, which drive progressive neuronal dysfunction and cognitive decline. Despite decades of research, current therapeutic strategies remain largely symptomatic, failing to halt or reverse disease development, underscoring an urgent need for effective disease‐modifying interventions [2, 3].

The multifaceted etiology of AD necessitates a multitarget pharmacological approach capable of simultaneously addressing various pathological pathways. Beyond the canonical Aβ and tau pathologies, emerging evidence highlights the roles of proteins like sortilin and clusterin in amyloid processing, clearance, and neuroprotection, rendering them compelling targets for comprehensive AD therapy [4]. Developing compounds that engage these diverse targets simultaneously represents a promising avenue for therapeutic breakthroughs.

Computational techniques have transformed drug discovery by significantly accelerating the identification of lead compounds. Molecular docking simulations offer a powerful initial screening tool, predicting ligand–protein binding affinities and providing atomic‐level insights into interaction mechanisms [5]. Crucially, the modern drug discovery paradigm integrates in silico ADME (absorption, distribution, metabolism, excretion) and toxicity prediction (e.g., ProTox‐II) early in the pipeline [6]. These computational screens are indispensable for filtering out compounds with unfavorable pharmacokinetic profiles or potential adverse effects, thereby mitigating the substantial costs and high attrition rates of experimental validation. This holistic computational strategy is paramount for identifying truly druggable candidates combining potent target engagement with acceptable bioavailability and safety [7].

Natural compounds, owing to their vast chemical diversity and inherent polypharmacological properties, offer an invaluable reservoir for novel drug discovery. Historically, various medicinal plants have been revered for their neurological benefits [8]. For instance, ginkgolide, a prominent constituent of *Ginkgo biloba*, is well documented for its neuroprotective and cognitive‐enhancing attributes in neurodegenerative conditions [9, 10]. Similarly, ginsenosides from *Panax ginseng* [11], berberine from *Berberis* species [12], and apigenin—a widely distributed flavonoid—have garnered significant attention for their reported neuroprotective, anti‐inflammatory, and anti‐amyloidogenic activities relevant to AD pathophysiology [13–15]. Their innate capacity for multitarget engagement positions them as prime candidates for computational exploration in AD [16].

Molecular docking simulation tools were employed to investigate the binding affinities and specific amino acid interactions of a carefully selected library of natural compounds with four key AD‐related protein targets: sortilin, clusterin, tau, and amyloid. While compounds like berberine and ginkgolide are widely accepted for their general neuroprotective effects, the molecular basis for their multitarget effectiveness in AD remains functionally dissociated across the literature [17–19]. To address this critical gap, this study provides the first computational assessment of this specific panel of natural compounds against a comprehensive network of four key AD targets. It will lead to the identification of potential candidates exhibiting promising multitarget binding capabilities, serving as a crucial initial step toward the discovery of novel AD therapeutics that would warrant further comprehensive ADME and toxicity evaluation.

## 2. Methodology

### 2.1. Computational Study Design Overview

This study employed an integrated ex vivo​ and in vitro literature and in silico analysis to identify potential multitarget natural compound candidates for AD therapy. The methodology involved target protein preparation, comprehensive ligand library construction, molecular docking simulations, and in silico assessment of ADME and toxicity profiles for promising compounds. The analysis focused on four key AD‐related proteins: sortilin‐related receptor (SORL1), clusterin, tau peptide, and amyloid.

### 2.2. Target Protein Preparation

The four target protein structures, SORL1 (PDB ID: 7VTO), clusterin (PDB ID: 7ZET), tau peptide (PDB ID: 7MKH), and amyloid (PDB ID: 5TXD), were retrieved from the Protein Data Bank (PDB) database (https://www.rcsb.org) [20]. Before docking, each protein structure underwent rigorous preparation using AutoDock Tools (Version 1.5.7). This involved removing all co‐crystallized ligands, water molecules, and other extraneous nonprotein entities. Polar hydrogen atoms were added, and gasteiger charges were assigned to all atoms to ensure proper electrostatic representation for docking calculations. The prepared protein structures were then saved in PDBQT format, compatible with AutoDock Vina [21].

### 2.3. Ligand Library Construction and Preparation

The selection of compounds for this study involved a thorough literature review focused on identifying 16 natural compounds with documented neuroprotective, anti‐inflammatory, antioxidant, or other mechanisms relevant to AD pathology, as well as those with a history of traditional medicinal use for cognitive health and neurodegenerative disorders. Selected natural compounds were beta‐carotene, cannabidiol, ginkgolide, harman, dronabinol, cyanidin, curcumin, berberine, resveratrol, astragaloside, allicin, ginsenocide, hesperidin, and apigenin. Additionally, three known drugs used in Alzheimer’s treatment were included: memantine, rivastigmine, and donepezil. The formulas and PubChem IDs of selected ligands and drugs are detailed in Table 1.

**Table 1:** Ligands, their formulas, and ligand IDs from PubChem.

Sr. no.	Ligands/compounds	Formula	PubChem IDs
1	4‐tert‐Amylphenol	C_11_H_16_O	6643
2	Allicin	C_6_H_10_OS_2_	65036
3	Apigenin	C_15_H_10_O_5_	5280443
4	Astragaloside	C_28_H_32_O_17_	5488387
5	Berberine	C_20_H_18_NO_4_^+^	2353
6	Beta‐carotene	C_40_H_56_	5280489
7	Cannabidiol	C_21_H_30_O_2_	644019
8	Curcumin	C_21_H_20_O_6_	969516
9	Cyanidin	C_15_H_11_O_6_^+^	128861
10	Dronabinol	C_21_H_30_O_2_	16078
11	Galantamine	C_17_H_21_NO_3_	9651
12	Ginkgolide	C_20_H_24_O_10_	6324617
13	Ginsenosides	C_30_H_52_O_2_	3086007
14	Harman	C_12_H_10_N_2_	5281404
15	Hesperidin	C_28_H_34_O_15_	10621
16	Resveratrol	C_14_H_12_O_3_	445154

*Drugs*			
1	Donepezil	C_24_H_29_NO_3_	3152
2	Memantine	C_12_H_21_N	4054
3	Rivastigmine	C_14_H_22_N_2_O_2_	77991

The 2D chemical structures (SMILES format) of these compounds were retrieved from the PubChem database (https://pubchem.ncbi.nlm.nih.gov/) [22]. For molecular docking, these ligands were converted from SMILES/SDF to PDB format using Open Babel GUI (Version 3.1.1) [23]. Subsequently, AutoDock Tools (Version 1.5.7) was used to prepare the ligands, which involved setting rotatable bonds and converting them to AutoDock Vina‐compatible PDBQT format.

### 2.4. Molecular Docking Simulations

Molecular docking simulations were executed using AutoDock Vina to evaluate the binding conformations and affinities of the 15 selected ligands with each of the four target proteins. Rigid docking was conducted, and grid center coordinates were set individually for each protein target to accurately capture its binding site. For sortilin, the binding pocket was centered in the negative *x*‐quadrant, with positive *y* and *z* coordinates. The grid center ranges were *x*: −18 to −22, *y*: 27 to 33, and *z*: 14 to 22. The clusterin binding site was situated in positive *x* and *z* regions, with y‐coordinates ranging from approximately −5 to 1. The coordinate ranges were *x*: 11 to 21, *y*: −5 to 1, and z: 26 to 35. The amyloid binding site was consistently located in a region near the origin, with small, negative *x* and *y* coordinates and a small positive *z* coordinate. The ranges were approximately *x*: −2.5 to −5.7, *y*: −0.7 to −3.0, and *z*: 2.3 to 4.2. Lastly, the TAU binding site was positioned in a unique area of the simulated space, far from the origin. All its grid center coordinates were large and positive, with ranges of approximately *x*: 167 to 178, *y*: 170 to 177, and *z*: 163 to 181. An exhaustiveness value of 10 was used for all docking runs, allowing for a comprehensive search for optimal binding poses. Best results were redocked through AutoDock Vina and reconfirmed through MTi‐AutoDock, DockThor, and Webina 1.0.5. LigPlot+ [24] was used for 2D interaction visualization; numeric interaction geometry was obtained from PLIP [25]. PLIP analysis of top‐ranked complexes from AutoDock Vina identified conserved hydrogen bonds and hydrophobic interactions that match known binding residues. PyMOL measurements show that key H‐bond distances remain within accepted geometric ranges (≤ 3.5 Å) and that prioritized poses are substantially buried in the pocket. The docking procedures were revalidated through redocking (RMSD < 2 Å) and binding‐site confirmation via SiteMap as well as CASTp after AutoDock Vina analysis.

### 2.5. In Silico ADME and Toxicity Prediction

The physicochemical, pharmacokinetic, and drug‐likeness properties of the selected ligands were assessed using the SwissADME [26] web server (http://www.swissadme.ch/). This analysis evaluated key ADME features, including gastrointestinal (GI) absorption, blood–brain barrier (BBB) permeability, and interaction with major metabolizing enzymes. Drug‐likeness was evaluated based on established criteria such as Lipinski’s Rule of Five [27], Ghose’s rule [28], Veber’s rule [29], and Muegge’s rule [30]. The ligands’ SMILES strings, obtained from the ChEMBL database (https://www.ebi.ac.uk/chembl/), served as input for the Swiss ADME server. Toxicity profiles, including mutagenicity, carcinogenicity, cytotoxicity, and immunotoxicity, were predicted using the ProTox‐3.0 web server (https://tox.charite.de/protox3/). This tool provides insights into potential adverse effects, contributing to the early identification of lead compounds with acceptable safety profiles.

### 2.6. Molecular Interaction Analysis

Following molecular docking simulations, the top‐ranked binding conformations for each ligand–protein complex, based on the lowest binding energy, were selected for detailed analysis. Visualization and analysis of the intermolecular interactions, including hydrogen bonds, hydrophobic interactions, pi‐stacking, and van der Waals forces, were performed using Discovery Studio (Version v24.1.0.23298) [31]. This enabled the identification of key amino acid residues involved in the binding interfaces, providing structural insights into the observed binding affinities.

## 3. Results

The comprehensive in silico analysis encompassed ADME and ProTox‐3.0 predictions for pharmacokinetic and safety profiles, followed by molecular docking simulations to assess binding affinities and interactions with four key AD targets: the sortilin receptor, clusterin receptor, Aβ peptide, and tau protein.

### 3.1. ADME and ProTox‐3.0 Analysis

**Figure 1:**
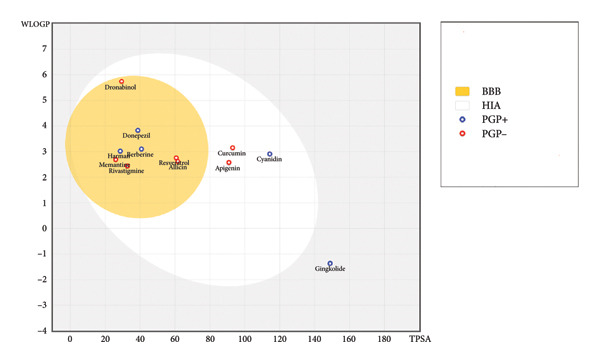
BOILED‐egg model of selected compounds. Where the yellow region (yolk) represents compounds predicted to penetrate the blood–brain barrier (BBB). The white region (egg white) represents compounds predicted to be absorbed by the human intestine (HIA). Blue circles (PGP+) indicate compounds that are substrates of P‐glycoprotein, an efflux pump. Red circles (PGP−) indicate compounds that are not substrates of P‐glycoprotein.

Most ligands, including 4‐tert‐amylphenol, allicin, apigenin, astragalosides, berberine, cannabidiol, curcumin, cyanidin, dronabinol, galantamine, harman, and resveratrol, demonstrated high GI absorption. Conversely, ginkgolide, ginsenosides, and hesperidin showed low GI absorption. BBB permeation varied, with 4‐tert‐amylphenol, allicin, astragalosides, berberine, cannabidiol, dronabinol, galantamine, harman, and resveratrol predicted as BBB permeant. Solubility (ESOL class) ranged from *very soluble* (allicin) to *poorly soluble* (dronabinol, ginsenosides), represented through Figure 1 which highlights the varying BBB and human intestine absorption (HIA) predictions.

Regarding drug‐likeness (Lipinski’s Rule of Five), many ligands (e.g., 4‐tert‐amylphenol, allicin, apigenin, berberine, curcumin, cyanidin, galantamine, ginkgolide, harman, and resveratrol) showed zero violations, indicating favorable oral bioavailability. However, astragalosides (2 violations), cannabidiol (1 violation), dronabinol (1 violation), ginsenosides (1 violation), and hesperidin (3 violations) exhibited deviations. Lead‐likeness also varied, with apigenin, cyanidin, and galantamine meeting criteria, while others showed violations. The bioavailability score was consistently 0.55 for most compounds, except hesperidin (0.17). Skin permeation rates also showed a wide range.

ProTox‐3.0 analysis revealed predicted hepatotoxicity (“Active”) for 4‐tert‐amylphenol, berberine, and ginkgolide, while all others were “Inactive.” Cytotoxicity was “Active” only for galantamine, with all other ligands predicted as “Inactive.” The ADME and ProTox‐3.0 results (ST2) for the three reference drugs show that donepezil (364.39 g/mol), memantine (215.76 g/mol), and rivastigmine (250.34 g/mol) all have high GI absorption and a bioavailability score of 0.55. Memantine and rivastigmine are predicted to be BBB permeant, while donepezil is not. All three drugs showed zero violations for drug‐likeness. For lead‐likeness, donepezil and memantine had 2 violations, while rivastigmine had 0. In terms of toxicity, donepezil was “Active” for cytotoxicity but “Inactive” for hepatotoxicity, whereas memantine and rivastigmine were “Inactive” for both.

### 3.2. AutoDock Vina Results: Sortilin Receptor

**Figure 2:**
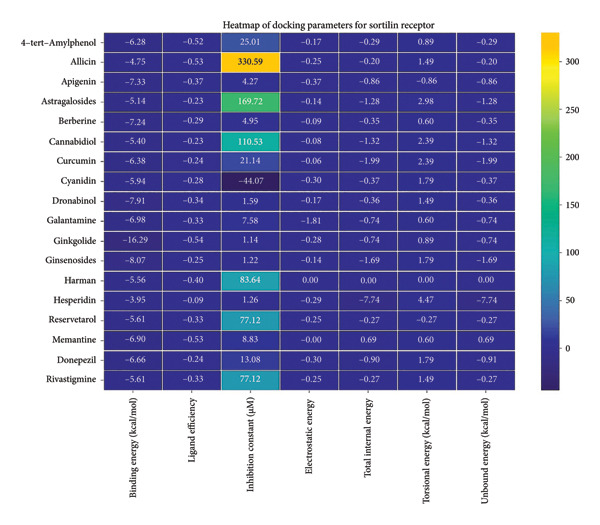
Characteristics of natural ligands and reference drugs for the sortilin receptor analyzed through AutoDock Vina.

The sortilin receptor demonstrated the highest affinity for ginkgolide (−16.29 kcal/mol), representing the strongest binding observed across all targets. Key interactions for ginkgolide involved hydrogen bonding with ARG292 and TYR318. Other strong binders included ginsenosides (−8.07 kcal/mol) interacting with ILE141 and MET139 and dronabinol (−7.91 kcal/mol); for instance, 4‐tert‐amylphenol formed highly favorable bonds with ARG292 (donor–acceptor D–A distance: 2.92, angle Ǻ and 3.11 Ǻ:164.68°, Figure 2 details the binding characteristics of all tested ligands. Table 2 shows the predicted hydrogen bond interactions for selected molecules and drugs against sortilin, clusterin, amyloid, and tau proteins.

**Table 2:** Predicted hydrogen bond interactions for selected molecules and drugs against sortilin, clusterin, amyloid, and tau proteins.

Sr. no.	Molecules	Sortilin			Clusterin			Amyloid			Tau		
		Key interacting residues	Distance D–A	Donor angle (°)	Key interacting residues	Distance D–A	Donor angle (°)	Key interacting residues	Distance D–A	Donor angle (°)	Key interacting residues	Distance D–A	Donor angle (°)
1	4‐tert‐Amylphenol	ARG292: HH 21	2.93	168.42	GLN349:HE22	2.78	136.03	ILE5:0, ILE5:HN	2.79	133.34	VAL339:HN	2.84	149.87
		TYR318: HN	3.11	164.68					3.05	161.79			

2	Allicin	SER283: HG 1	2.64	159.74	ASP188:HG	3.55	161.85	GLY3:HN	2.84	165.69	VAL339:HN	2.69	159.07
		ARG 292: HE 1	2.82	162.25									

3	Apigenin	ILE494: HN SER541:HN	3.11 3.02	164.85 147.95	LYS351:HZ2 GLN361:HE21	3.16 2.78	158.94 156.50	LYS2:0, LYS2:HZ2	3.03 2.96	169.52 161.58	GLY326:0 VAL363:0 ILE328:HN	3.13 2.99 2.91	153.85 159.52 161.21

4	Astragalosides	TRP83:HN 1	2.98	158.76	LYS351:HZ2	2.95	146.15	GLY3:HN	3.05	159.41	GLY326:0	3.09	164.72
		SER541:HN 1	2.84	157.23									

5	Berberine	MET139: HN	3.17	158.84	ASN291:HD2	2.82	148.62	ILE5:0	3.26	163.06	GLN351:HN	2.74	128.07
		TYR189: HN	2.79	130.87									

6	Cannabidiol	TRP228: HN	2.87	172.18	ARG 282: HE 1	2.78	165.12	GLY3:0	2.79	123.57	ARG282:0	2.78	165.12

7	Curcumin	SER432: HN	2.96	156.85	GLU364:OE1	2.63	146.06	ASN1:HD22	2.97	176.29	VAL339:HN	3.17	149.87
		SER447:HN	3.11	125.35							LEU357:HN	3.00	151.75
		GLY544:HN	3.01	139.21							SER341:HN	2.98	154.17
											VAL337:0	3.00	154.80

8	Cyanidin	ILE141:HN	2.65	127.48	THR293	3.46	112.51	ILE5:0	3.00	145.01	ILE360:0	3.35	154.29
								ILE5:HN	2.76	168.52			
								ASN1:0	2.81	154.59			

9	Dronabinol	ARG292:HH	3.01	134.22	ASN43:OD1	2.61	125.76	LYS2:HZ2	3.00	152.17	ILE354:0	3.13	137.08

10	Galantamine	ARG403:HH21	2.91	168.21	GLN255:HE22	2.80	158.22	GLY3	3.27	120.26	VAL337:0	3.93	103.71
											VAL339:HN	2.90	146.27

11	Ginkgolide	TYR318:HN1	3.45	161.86	HIS205:HD1	3.24	135.06	ILE5	3.14	162.26	HIS329:HD1	3.05	170.04

12	Ginsenosides	ILE141:HN1 MET139: O	2.77 2.89	163.69 158.28	PHE240:HN,	3.00	151.37	ALA4:0, ALA4:HN, LYS2:HZ2	2.56 2.83 3.10	160.82 157.52 163.98	HIS329:0 PRO364:0	2.692.86	126.86 157.86

13	Harman	ILE141:O	3.12	166.19	GLU287:OE2	2.77	160.48	GLY3:0	2.92	143.20	ASN327:0D1	3.00	147.86

14	Hesperidin	TRP228:HN	2.79	132.67	SER161: O, THR 283: O, GLU287:OE1, THR293:HG1,	3.06 2.88 2.91 2.75	147.39 156.25 154.84 126.10	LYS2:HZ, GLY3:HN, GLY3:0	2.71 3.01 2.83	159.85 157.82 156.74	ASN359:0	2.88	152.68

15	Resveratrol	ILE494:HN 1	3.04	164.05	PRO212:0, ALA373:0	2.94 3.03	139.48 145.09	LYS2:HZ, GLY3:HN GLY3:0	2.99 3.05 2.96	131.58 158.19 145.43	ASP358:HN, ILE360:0, SER356:0G, LYS353:HZ3	3.04 2.84 2.91 3.05	157.89 130.96 165.71 162.79

1	Memantine	GLN400:O	2.71	113.33	SER356:0	3.28	114.92	ILE5:HN	2.80	159.52	No Hydrogen Bound	—	—

2	Donepezil	TYR189:O	3.18	119.31	GLY378	2.62	102.11	ILE5:0	3.46	130.03	HIS362:HN	3.56	144.19

3	Rivastigmine	ILE494:HN	3.04	164.05	THR293:HG1	3.03	140.22	GLY3:HN	2.88	176.39	VAL339:HN	2.98	159.86

### 3.3. AutoDock Vina Results: Clusterin Receptor

Ginkgolide showed the strongest binding at −13.98 kcal/mol, interacting with HIS205. Other potent ligands were ginsenosides (−8.02 kcal/mol), which formed contacts with PHE240, and berberine (−6.46 kcal/mol), interacting with ASN291. Resveratrol also exhibited favorable binding (−6.34 kcal/mol), forming H‐bonds with PRO212 and ALA373. Cannabidiol had the weakest binding at −3.56 kcal/mol, interacting with ARG292:HE1. The weakest binder was cyanidin (−3.92 kcal/mol), which showed no hydrogen bond interactions.

**Figure 3:**
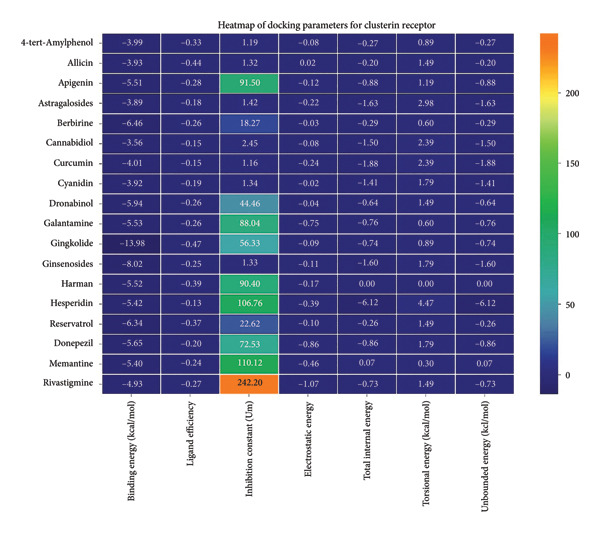
Characteristics of natural ligands and reference drugs for the clusterin receptor analyzed through AutoDock Vina.

For reference drugs, donepezil (−5.65 kcal/mol) with GLY378, Figure 3 illustrates the binding characteristics of natural ligands and reference drugs for the clusterin receptor.

### 3.4. AutoDock Vina Results: Aβ Peptide

Against the core Aβ peptide, ginkgolide again showed the lowest binding energy (−8.40 kcal/mol), though no direct hydrogen bond interactions were detected in the lowest‐energy pose. Ginsenosides (−4.36 kcal/mol) demonstrated stable interactions, notably forming H‐bonds with ALA4 and LYS2.

**Figure 4:**
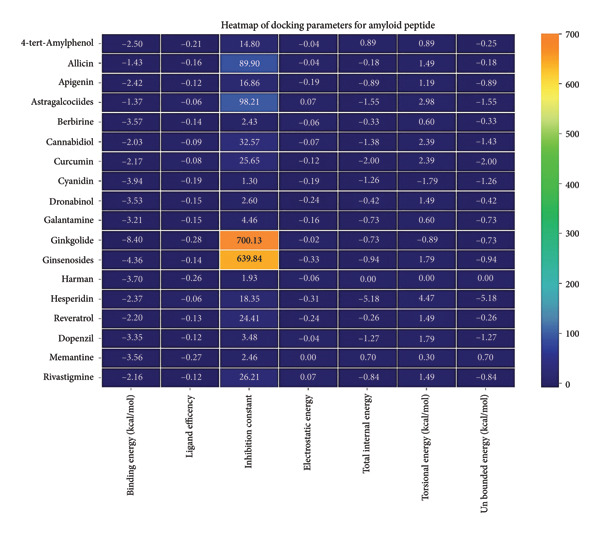
Characteristics of natural ligands and reference drugs for the amyloid‐beta peptide analyzed through AutoDock Vina.

Ginsenosides (−4.36 kcal/mol) formed a highly optimal bond with ALA4 (carbonyl oxygen [O] donor) with a short distance (2.56 Ǻ) and a near line angle (160.82°). Cyanidin (−3.94 kcal/mol) also formed H‐bonds with ILE5 and ASN1. The reference drug memantine exhibited significant binding at −3.56 kcal/mol, forming an H‐bond with the critical amyloidogenic residue ILE5 D‐A 2.80 Ǻ, angle: 159.52°. Figure 4 summarizes the binding characteristics for Aβ peptide.

### 3.5. AutoDock Vina Results: Tau Protein

**Figure 5:**
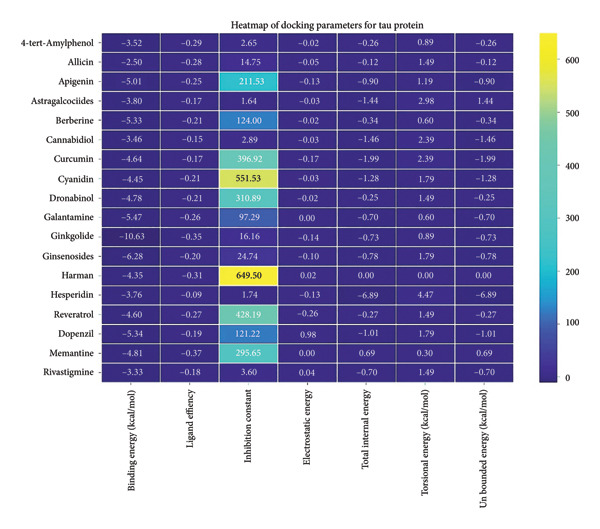
Characteristics of natural ligands and reference drugs for the tau protein analyzed through AutoDock Vina.

For the tau protein repeat domain, ginkgolide recorded a strong binding energy of −10.63 kcal/mol, with a key stabilizing interaction involving HIS329. This interaction was characterized by a D–A distance of 3.05 Ǻ and an excellent angle of 170.04°. Other top binding compounds included ginsenosides (−6.28 kcal/mol), also interacting with HIS329 and PRO364, and galantamine (−5.47 kcal/mol), forming bonds with VAL337 and VAL339. Berberine bound at −5.33 kcal/mol via GLN351. Apigenin exhibited favorable interaction with the tau repeat domain via a hydrogen bond with ILE 328 (D–A: 2.91 Ǻ; angle 161.21°), highlighting the high quality of the interaction. Among the reference drugs, donepezil bound at −5.34 kcal/mol. Figure 5 details the binding characteristics for tau protein.

**Figure 6:**
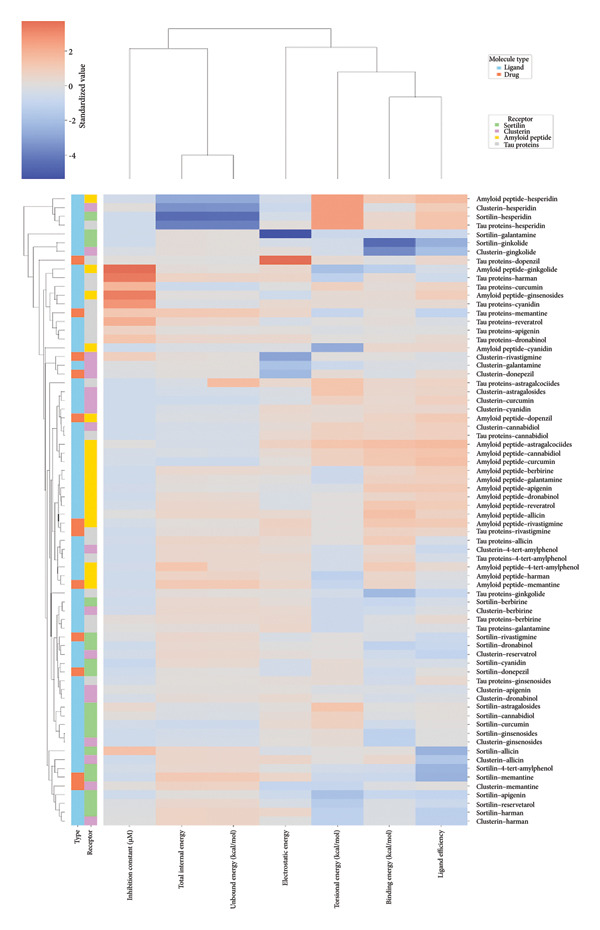
Heatmap and hierarchical clustering of AutoDock Vina docking results for natural ligands and reference drugs against Alzheimer’s disease‐related receptors.

The heatmap in Figure 6 provides a comprehensive visual interpretation of the AutoDock Vina docking analysis of natural ligands (light blue‐coded) and reference drugs (orange‐coded) against four key AD‐related receptors. Ginkgolide consistently demonstrates strong (deep red coloration) binding energies across all four receptors: sortilin (blue‐coded), clusterin (green‐coded), Aβ peptide (yellow‐coded), and tau proteins (purple‐coded). Natural ligands exhibited varied yet significant binding profiles; ginsenosides showed strong affinities for tau proteins and Aβ peptide, with moderate binding to sortilin and clusterin. Dronabinol displayed particularly strong interactions with sortilin and Aβ peptide, while berberine and apigenin consistently showed favorable binding across multiple targets, underscoring their broad spectrum of action. In stark contrast, hesperidin consistently presented among the weakest binding affinities across most receptors (indicated by blue/lighter colors), suggesting limited direct molecular interaction.

Comparative analysis against the established reference drugs—memantine, donepezil, and rivastigmine—revealed that several natural compounds, most notably ginkgolide, achieved binding affinities that were superior to these therapeutic agents across various AD‐related targets, highlighting their significant potential as alternatives or adjuncts. The hierarchical clustering patterns displayed by the dendrograms provided further insights: Molecules grouped together along the *y*‐axis (ligand–receptor pairs) often shared similar overall binding characteristics. For example, ginkgolide consistently clustered within highly favorable binding groups across multiple receptors (Figure 7), and groups like ginsenosides and cyanidin showed similar strong binding patterns toward tau proteins. Notably, certain natural ligands, such as allicin and harman, clustered with other smaller, more drug‐like natural compounds. Importantly, this clustering also revealed instances where natural ligands exhibited similar binding profiles to reference drugs like memantine, donepezil, or rivastigmine, suggesting potentially shared binding modes or therapeutic mechanisms.

Figure 7Molecular docking analysis of ginkgolide interactions with Alzheimer’s disease‐related proteins: Molecular docking analysis of ginkgolide’s interactions with four key proteins—sortilin, clusterin, amyloid‐beta peptide, and tau protein. Column (a, d, g, j) shows the receptor–ligand interaction surface, providing a visual representation of how ginkgolide binds to the active site of each protein. The second column (b, e, h, k) depicts the secondary structure and ligand interactions, offering insights into the specific amino acid residues and structural motifs convoluted in binding. The third column (c, f, i, l) presents the LigPlot+ interaction diagram, providing a 2D schematic of the ligand–protein interactions, highlighting hydrogen bonds and hydrophobic contacts between ginkgolide and the surrounding amino acids of the binding site.

**Figure:**
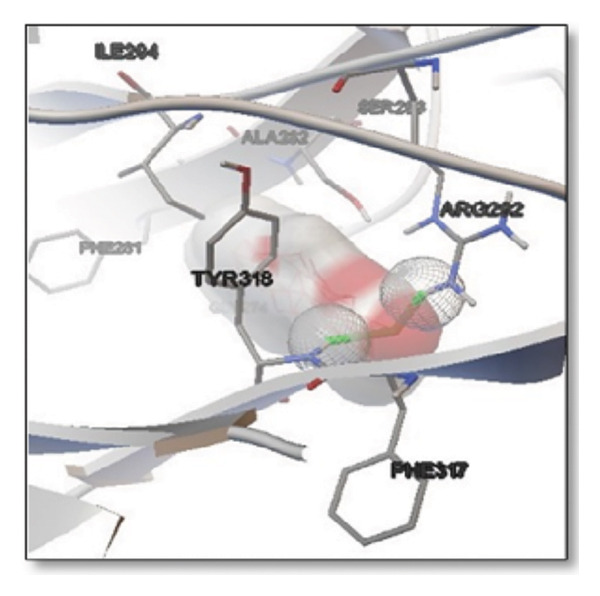
(a)

**Figure:**
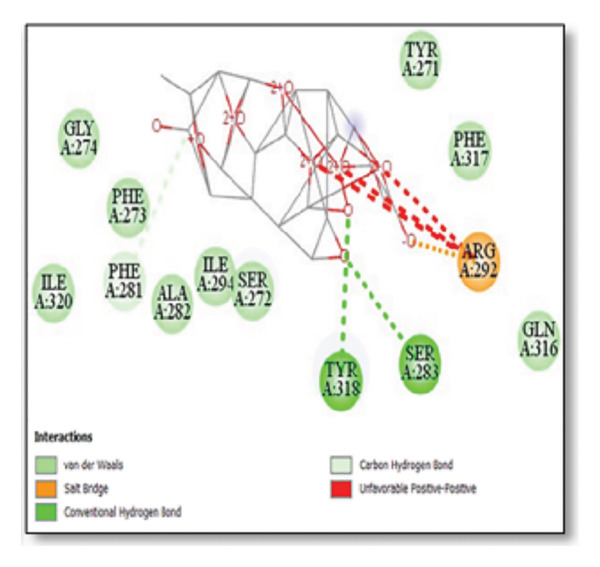
(b)

**Figure:**
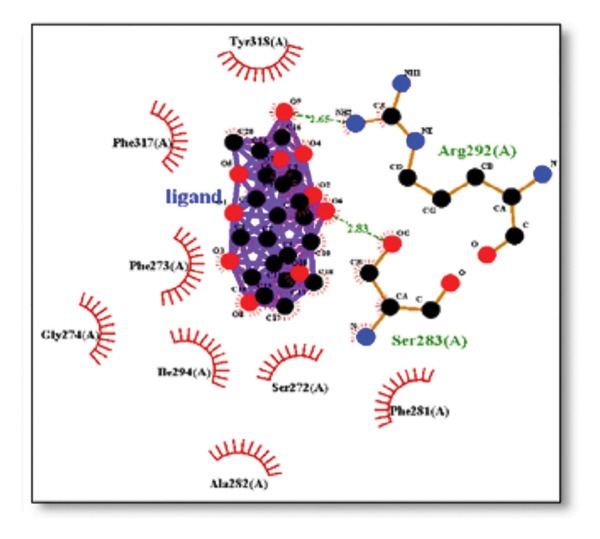
(c)

**Figure:**
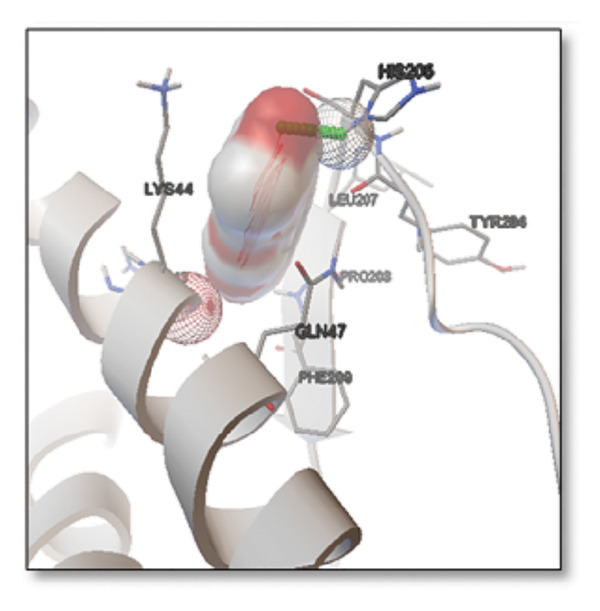
(d)

**Figure:**
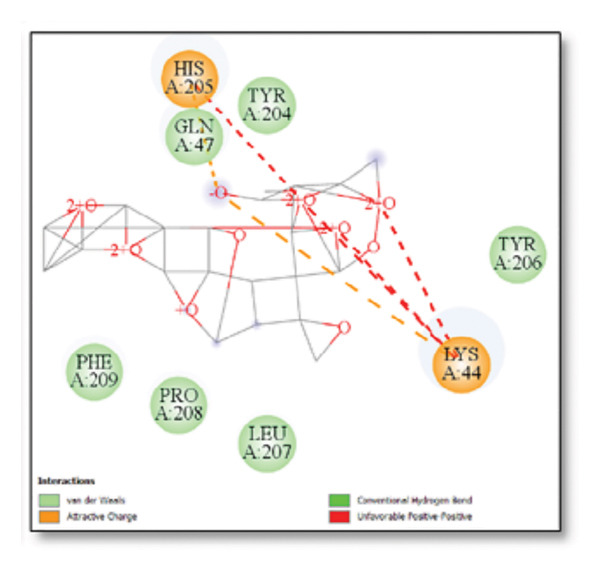
(e)

**Figure:**
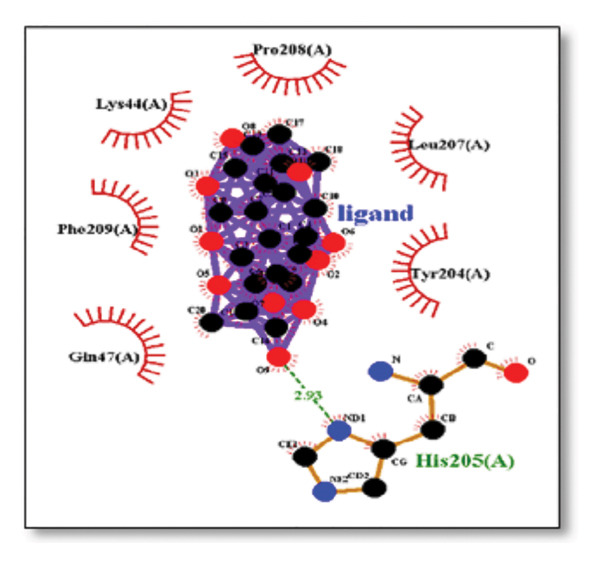
(f)

**Figure:**
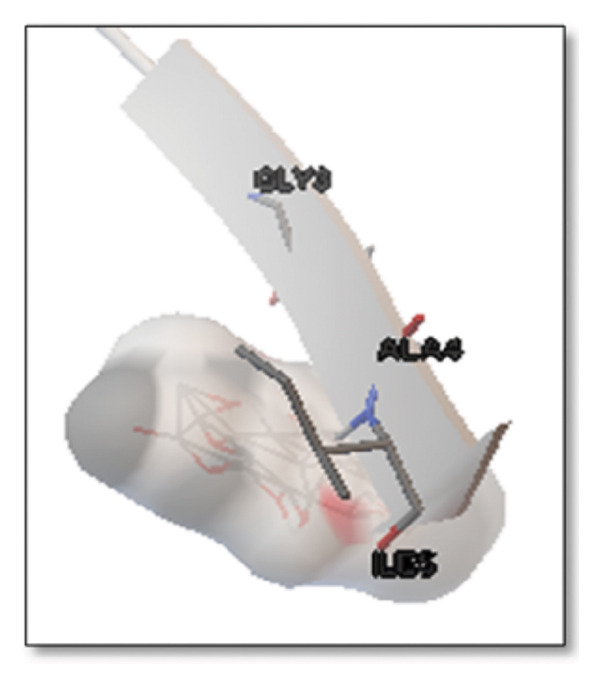
(g)

**Figure:**
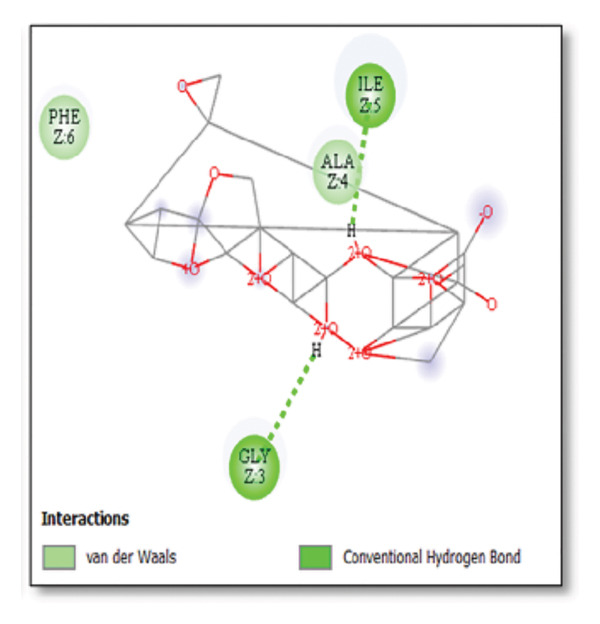
(h)

**Figure:**
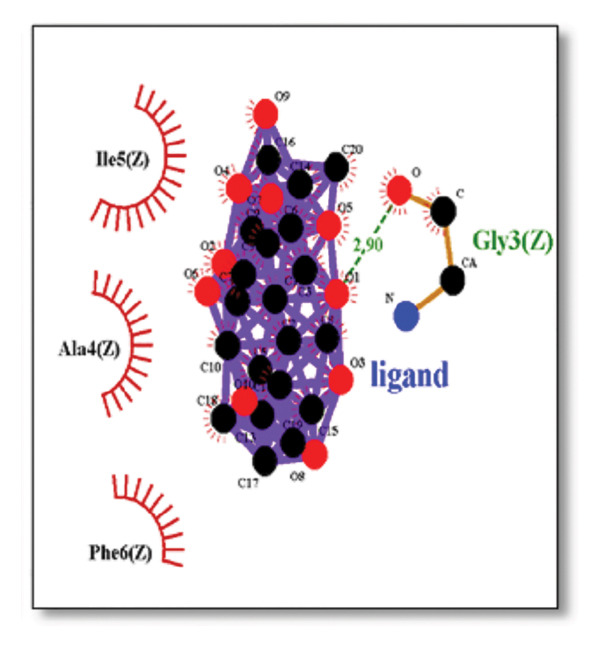
(i)

**Figure:**
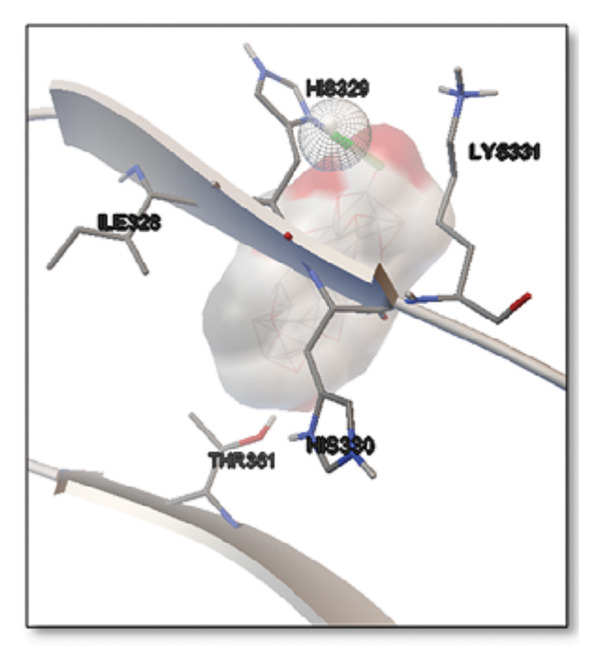
(j)

**Figure:**
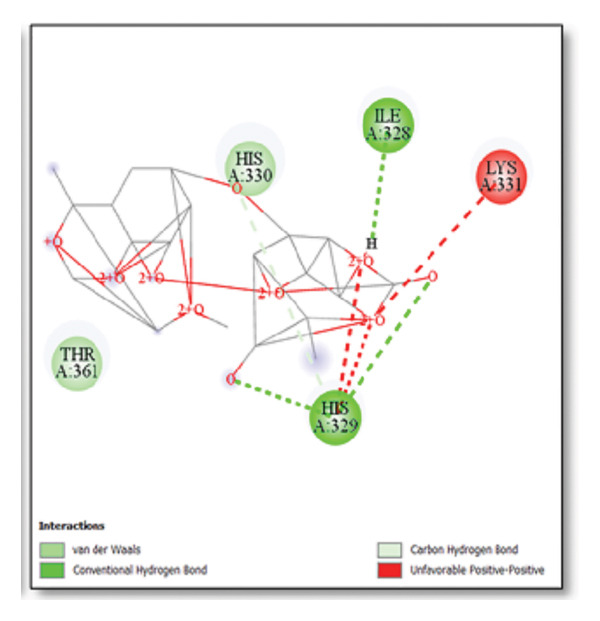
(k)

**Figure:**
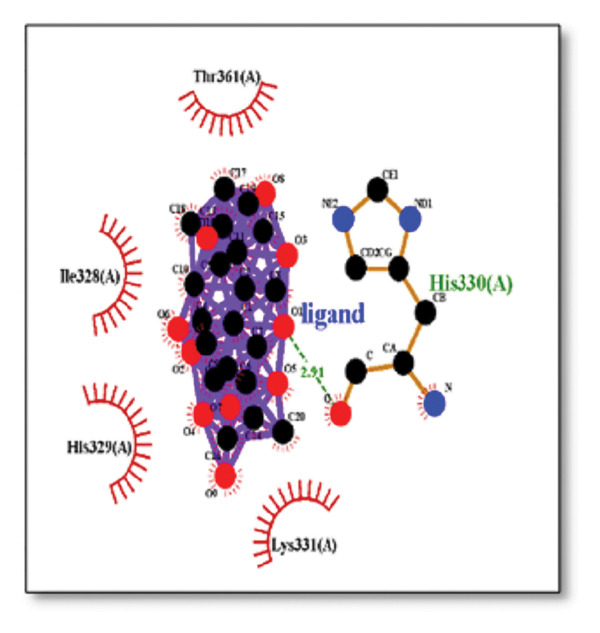
(l)

Multiplatform docking (MTi‐AutoDock, DockThor, and Webina) and PLIP analysis confirmed consistent binding orientations and affinities. Key interactions (H‐bonds, hydrophobic contacts) aligned with known active‐site residues. Redocking RMSD values (< 2 Å) indicated high pose reproducibility. Binding‐site validation confirmed biologically relevant cavities.

## 4. Discussion

The intractable complexity of AD pathology, encompassing multifaceted aberrations from Aβ aggregation and tau hyperphosphorylation to widespread neuroinflammation and synaptic dysfunction, urgently necessitates therapeutic strategies capable of simultaneously modulating multiple targets. This study addresses this imperative through a comprehensive in silico framework, integrating ADME and ProTox‐3 analyses with AutoDock Vina molecular docking against four pivotal AD‐associated proteins: sortilin receptor, clusterin receptor, Aβ peptide, and tau protein. This approach provides a robust preliminary assessment of fifteen novel ligands, many derived from natural sources, alongside three established AD reference drugs (donepezil, memantine, and rivastigmine), yielding critical insights for future multitarget drug discovery in AD.

Apigenin and allicin have favorable predicted ADME properties, suggesting they could be developed into traditional oral drugs. Ginkgolide and ginsenoside, however, exhibit less favorable ADME profiles, particularly low permeability across the BBB, which is critical for an Alzheimer’s drug. Despite these challenges, ginkgolide stands out due to its multitarget activity and a history of research supporting its neuroprotective, anti‐inflammatory, and antioxidant effects. This established therapeutic promise necessitates overcoming its ADME drawbacks. To ensure physiological relevance and clinical translation, strategic CNS delivery approaches must be employed. Evidence‐based methods, such as utilizing lipid‐based nanocarriers or chemical modifications to exploit receptor‐mediated transcytosis across the BBB, offer viable paths to achieve therapeutic concentrations of ginkgolide at its targets.

Ginkgolide, a diterpenoid lactone derived from *G. biloba*, consistently demonstrated the predicted binding affinities of all screened compounds against sortilin (−16.29 kcal/mol), clusterin, and tau protein, significantly surpassing the affinities of the three reference drugs tested. The computational evidence provides a compelling molecular basis for the widely reported, yet often mechanistically unclarified, neuroprotective and cognitive‐enhancing benefits of *G. biloba* extracts in numerous neurodegenerative disorders, comprising AD and Parkinson’s disease [32–34]. Further findings [35–37] suggest that ginkgolide’s observed broad‐spectrum clinical effects might be attributable, at least in part, to its direct engagement with multiple critical AD targets, moving beyond general antioxidant or anti‐inflammatory actions to specific protein interactions.

The in silico predictions for ginkgolide’s interactions with sortilin, clusterin, and tau are well‐supported by the established structural and functional mechanisms of these proteins in scientific literature. For sortilin, the predicted interaction with ARG292 and TYR318 is consistent with the protein’s known structure. Studies have shown that its large, ten‐bladed β‐propeller domain is the primary ligand‐binding site; ARG292, often positioned near the VPS10P domain, likely provides critical electrostatic stabilization, while TYR318 contributes $\pi‐\pi$ or $\pi$‐cation interactions, collectively stabilizing the ligand–receptor complex. This binding site overlaps with regions known to undergo conformational change during ligand recognition, suggesting ginkgolide may allosterically modulate the receptor’s trafficking function. A 2017 study by [38], for example, demonstrated how conformational changes in this domain, caused by pH, disrupt binding sites. Similarly, a review by Quistgaard et al. [39] highlights how the architecture of the VPS10 domain, which includes these residues, is vital for its function. For clusterin, the predicted interaction with HIS205 is supported by the protein’s established role as a molecular chaperone that prevents protein aggregation.

Histidine residues are frequently involved in coordinating chaperone‐substrate interactions. The binding of ginkgolide at HIS205 suggests it may stabilize the active conformation of the chaperone, thereby enhancing its ability to bind to and inhibit the formation of amyloid fibrils, a key mechanism reinforced by recent research published [40]. This research has consistently reinforced that clusterin’s ability to bind to and inhibit the formation of amyloid fibrils is a key mechanism of action, particularly in neurodegenerative diseases. While direct mention of HIS205 is rare, its chaperone function is broadly understood to rely on such key residues. Elias et al. [41] and Carini et al. [42] confirm that clusterin’s role in inhibiting aggregation is a major focus of current research. Finally, the predicted binding to HIS329 in tau is highly significant and directly corroborated by recent literature. HIS329 is explicitly located within the microtubule‐binding repeat domain, which is the primary site for tau’s pathological self‐aggregation. Ginkgolide’s predicted binding at this position is a direct mechanism of aggregation inhibition, suggesting it acts as a steric wedge or “capping agent” to prevent the formation of toxic beta sheet structures, aligning with similar strategies explored. Studies have explicitly identified histidine residues within the microtubule‐binding repeat domain as participants in tau’s self‐aggregation process. Hernández et al. [43] specifically names residues like H329 as being involved in this mechanism. Molecular docking and simulation studies, such as one from 2025 by Saha and Natarajan [44], also focus on targeting this domain with small molecules to inhibit tau pathology, providing strong support for the in silico predictions.

Beyond ginkgolide, several other natural product‐derived ligands demonstrated significant multitarget potential, reinforcing the growing recognition of phytocompounds as rich sources for complex disease therapeutics. Ginsenosides, core triterpenoid saponins from ginseng, showed strong binding to sortilin, clusterin, and Aβ peptide. The computational modeling and molecular docking simulations performed in this study identified ginsenosides that exhibit a strong binding affinity for several key AD receptors. This novel finding is well‐supported by prior in vivo literature, which demonstrates the neuroprotective efficacy of these compounds. The docking analysis revealed specific molecular‐level interactions that underpin this strong binding. For instance, ginsenosides were found to interact with the sortilin receptor, forming key contacts with amino acid residues ILE141 and MET139. The functional significance of this interaction is that these hydrophobic residues are positioned at the entrance or periphery of the main ligand cavity. Binding here suggests an allosteric modulatory mechanism, where the ginsenosides could induce a conformational shift that impacts sortilin’s crucial role in the trafficking and endocytosis of the amyloid precursor protein (APP), potentially leading to reduced Aβ production and clearance [45]. The binding of ginsenosides at these specific residues could therefore modulate sortilin’s function, leading to reduced Aβ pathology.

Similarly, interactions with the clusterin receptor (also known as ApoJ) were mediated through a crucial binding site involving PHE240. PHE240 is a key hydrophobic residue within clusterin’s large chaperone domain. Our findings suggest that ginsenosides use a hydrophobic interaction at this specific residue to modulate the chaperone activity, thereby potentially stabilizing clusterin in a conformation that effectively prevents $\text{A}\beta$ from forming toxic oligomers [46]. Aβ peptide ginsenosides were shown to interact directly with ALA4 and LYS2. These particular residues are located in a critical hydrophobic region of the Aβ peptide, which is essential for its initial self‐association and subsequent aggregation into neurotoxic oligomers and fibrils. The ginsenoside interaction at these sites provides a compelling direct mechanism for how these compounds could inhibit Aβ aggregation, a central pathological event in AD [47].

The broad pharmacological relevance of ginsenosides further supports their multitarget potential. Prior in vivo research demonstrates that these compounds exhibit anticonvulsant, antipsychotic, analgesic, and antifatigue activity, while also enhancing cognitive performance. This is attributed to the inhibition of tyrosine hydroxylase, which reduces locomotor dysfunction and protects against neuronal cell death [48]. Specific ginsenosides have been shown to reduce excitotoxicity, provide antioxidant and anti‐inflammatory effects, and decrease dopaminergic cell loss. These actions prevent the development of locomotion deficits by blocking the Jun N‐terminal kinase signaling pathway and reducing α‐synuclein aggregates [49]. As an adaptogen, the compounds activate the nuclear factor erythroid 2‐related factor 2 pathway while inhibiting mitogen‐activated protein kinases and nuclear factor kappa‐light‐chain‐enhancer of activated B cells signaling. This dual action, facilitated by a variety of ginsenosides, provides anti‐apoptosis, anti‐inflammatory, and antioxidant benefits in preclinical models [50].

Collectively, these mechanistic insights derived from our computational modeling, combined with strong support from existing literature on sortilin, clusterin, and Aβ aggregation, provide a robust validation for our findings. They suggest that ginsenosides are not only potent multitarget binders but also promising therapeutic candidates for the treatment of AD by interfering with multiple pathological pathways.

Abbaoui et al. [51] conducted an in silico analysis focusing on AD acetylcholinesterase (AChE) as the target protein and examined carnosic acid and related abietane‐type diterpenes from rosemary. Analysis revealed favorable ADME/T profiles and robust binding affinities (−5.560 to −7.270 Kcal/mol) with AChE. Tedeschi et al. [52] examined garlic and its components in experimental cellular or mouse models of AD. The review highlighted the beneficial antioxidant and neuroprotective anti‐inflammatory properties of allicin and AGE contained in garlic extracts. Apigenin (4,5,7‐trihydroxy flavone) is a natural flavone in fruits, parsley, celery, and chamomile [53]. Apigenin, a ubiquitous flavonoid, demonstrated favorable binding to sortilin and tau. Apigenin’s interactions with GLY326, VAL363, and ILE328 in Tau are consistent with the known strategies of tau aggregation inhibitors [54] that target residues within its repeat domain [55]. Mechanistically, these residues form part of the core aggregation motif, suggesting apigenin functions as a small molecule inhibitor by sterically hindering the $\beta$‐sheet stacking essential for self‐assembly. Studies indicate that it has antioxidant, neuroprotective, anti‐inflammatory, and anti‐Aβ aggregation activities [56]. More crucially, it can pass the BBB. As per Siddique et al. [57], apigenin is powerful in lowering the AD symptoms, which are mimicked in the transgenic *Drosophila* model of AD. It is concluded that apigenin inhibits the activity of acetylcholinesterase and the formation of Aβ‐42 aggregates.

Dourado et al. [58] assessed apigenin’s neuroprotective and neuroimmunomodulatory potential by utilizing in vitro models of AD‐related neuroinflammation. Similarly, berberine exhibited promising interactions with sortilin, clusterin, and Aβ. Berberine’s predicted binding to ILE5 in the Aβ peptide is highly relevant, given that ILE5 is a crucial residue within the core amyloidogenic sequence, suggesting a direct interference with Aβ aggregation. ILE5 is a crucial hydrophobic residue located within the core amyloidogenic sequence. This suggests berberine acts as a hydrophobic anchor, directly interfering with the $\text{A}\beta$ self‐aggregation initiation step. Similarly, memantine, a known NMDA receptor antagonist [59], surprisingly exhibited strong binding to both sortilin (GLN400) and the Aβ peptide (ILE5). Its interaction with ILE5 in Aβ is particularly noteworthy, given the hydrophobic nature of both memantine and the Aβ core, suggesting a plausible direct interaction that could contribute to its reported anti‐aggregation properties in some studies [60]. This unexpected binding profile suggests a potential previously unrecognized multitarget mechanism for memantine that contributes to its clinical benefits beyond primary NMDA antagonism, underscoring the value of systematic multitarget screening for uncovering novel therapeutic pathways even in well‐characterized drugs. Donepezil also showed reasonable binding to sortilin and tau, providing further context. These comparisons not only validate in silico approach but also highlight the potential for our novel ligands to rival or even surpass the binding profiles of existing drugs, potentially offering improved efficacy through more comprehensive multitarget modulation.

The study demonstrates the power of an integrated in silico approach for rapidly identifying and prioritizing multitarget drug candidates for complex diseases like AD. The predicted binding affinities represent a theoretical interaction likelihood. The inhibition constants derived are estimates and require precise experimental quantification. Compelling in silico profiles of ginkgolide, ginsenosides, berberine, and apigenin necessitate rigorous experimental validation. The next steps include direct in vitro binding assays (e.g., surface plasmon resonance or isothermal titration calorimetry) to confirm binding affinities and functional cell‐based assays to elucidate the precise biological consequences of these interactions (e.g., inhibition of Aβ aggregation, modulation of tau phosphorylation pathways, or impact on sortilin‐ or clusterin‐mediated signaling). Subsequent longer‐timescale molecular dynamics simulations would be critical to understand the dynamic stability of these ligand–protein complexes and explore detailed binding mechanisms. Finally, comprehensive in vivo studies in established AD animal models will be essential to assess their therapeutic efficacy, pharmacokinetic properties, and long‐term safety, ultimately paving the way for translational development. This systematic progression from computational discovery to robust experimental validation is crucial for accelerating the development of multitarget therapeutics for AD.

## 5. Conclusion

This in silico investigation fundamentally reshapes the search for AD therapeutics, unveiling a potent new class of multitarget ligand candidates through systematic evaluation against four critical AD proteins: sortilin, clusterin, tau, and amyloid. Leveraging an advanced computational pipeline encompassing molecular docking of ginkgolide unequivocally stands as a breakthrough lead and requires an advanced drug delivery method. Its consistently lower binding affinities across all four targets, demonstrably surpassing even established AD drugs like memantine, redefine its therapeutic potential. Natural ligands, including ginsenosides, berberine, and apigenin, showcase compelling multitarget engagement, bolstered by favorable pharmacokinetic and safety profiles that designate them as truly “druggable” entities. While these in silico discoveries ignite immense promise, future studies should employ MM‐PBSA or MM‐GBSA to further quantify binding affinities; rigorous experimental validation remains the indispensable next frontier and a crucial bridge to confirm predicted efficacy, assess functional modulation, and fully characterize pharmacological profiles, ultimately accelerating the journey from computational insight to tangible clinical intervention.

## Ethics Statement

This study did not involve human or animal subjects.

## Consent

This study did not involve human subjects.

## Disclosure

All authors have read and agreed to publish the current version of the manuscript in its present form.

## Conflicts of Interest

The authors declare no conflicts of interest.

## Author Contributions

The authors listed have contributed equally to this work.

## Funding

This study received no external funding.

## Supporting Information

The Supporting data of this research is available in the attached Supporting file in which tables are represented as T1 to T5, and figures are represented as S1 to S72.

## Supporting information


**Supporting Information** Additional supporting information can be found online in the Supporting Information section.

## Data Availability

The data that support the findings of this study are available from the corresponding author upon reasonable request.
