# Supplementary material for: Computational Identification of Potent Multitarget Natural Ligands for Alzheimer’s Disease Therapeutics
Source: Scientifica (Cairo). 2025 Dec 29;2025:1132636. doi: 10.1155/sci5/1132636 (PMC12782343; doi:10.1155/sci5/1132636)
Supplement: Supplementary file 1 — Supporting Information Additional supporting information can be found online in the Supporting Information section. [file SCI5-2025-1132636-s001.docx]

**SUPPLEMENTARY DATA**

**Computational Identification of Potent Multi-Target Natural Ligands for Alzheimer's Disease Therapeutics**

Nadia Sharif ^1^, Ayesha Bibi ^2^, Rakhshinda Sadiq ^1^, Iffat Ullah ^3,4^, Abdul Rauf ^3,4^, Muhammad Tayyab Arshad ^5^, Hafsa Zamir ^6^, Sawaira gull ^6^, Taqwa Anwar ^6^, Emmanuel Laryea ^7^

1. Department of Biotechnology, Women University Mardan, 23200, Pakistan ([nadiasharif.s7@gmail.com](mailto:nadiasharif.s7@gmail.com)) ([rakhshinda_sadiq@yahoo.com](mailto:rakhshinda_sadiq@yahoo.com))
2. Department of Human Nutrition and Dietetics, Women University Mardan, 23200, Pakistan ([ayeshabb2009@yahoo.com](mailto:ayeshabb2009@yahoo.com))
3. Department of Pharmaceutical Chemistry, Faculty of Pharmaceutical Sciences, Prince of Songkla University, Hat Yai 90110, Songkhla, Thailand ([rauf31015@gmail.com](mailto:rauf31015@gmail.com)) ([Iffatph126@gmail.com](mailto:Iffatph126@gmail.com))
4. Drug Delivery System Excellence Center, Faculty of Pharmaceutical Sciences, Prince of Songkla University, Hat Yai 90110, Songkhla, Thailand
5. Functional Food and Nutrition Program, Center of Excellence in Functional Foods and Gastronomy, Faculty of Agro-Industry, Prince of Songkla University, Hat Yai, Songkhla, Thailand ([tayyabarshad5512@gmail.com](mailto:tayyabarshad5512@gmail.com))
6. Department of Zoology, Women University Mardan,23200, Pakistan ([zhafsa555@gmail.com](mailto:zhafsa555@gmail.com)) ([sawairagull9@gmail.com](mailto:sawairagull9@gmail.com)) ([taqwaanwar2020@gmail.com](mailto:taqwaanwar2020@gmail.com))
7. Department of Food Science and Technology, Kwame Nkrumah University of Science and Technology, Kumasi, Ghana ([etettehlaryea@gmail.com](mailto:etettehlaryea@gmail.com))

***Corresponding author**

Emmanuel Laryea ([etettehlaryea@gmail.com](mailto:etettehlaryea@gmail.com))

This supplementary file contains information supporting the computational identification of potent multi-target natural ligands for Alzheimer disease therapeutics. Listed below tables and figures provide detailed insights into the receptor structures, molecular docking results, and interaction analyses. Specifically, this file includes: (1) receptor details (T1), (2) AutoDock Vina results for Sortilin, Clustrin, Amyloid peptide, and Tau protein (T2-T5), and (3) visualizations of receptor-ligand interactions (Figures S1-S72).

**T1: Details of the receptor Sortilin, Clustrin, Tau and amyloid peptide including its PDB (IDs) three-dimensional structures.**

| **Sr.No.** | **Protein name** | **Pdb IDs** | **Structures** |
| --- | --- | --- | --- |
| 1. | Sortilin related receptor(SORL1) | 7VTO | 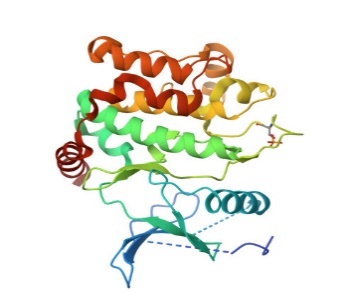 |
| 2. | Clusterin | 7ZET | 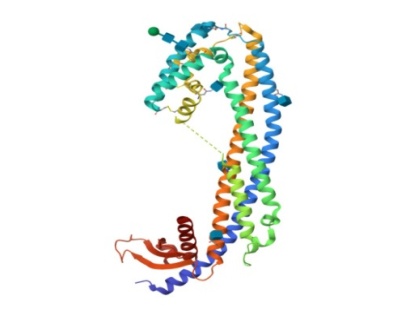 |
| 3. | Tau Peptide | 7mkh | 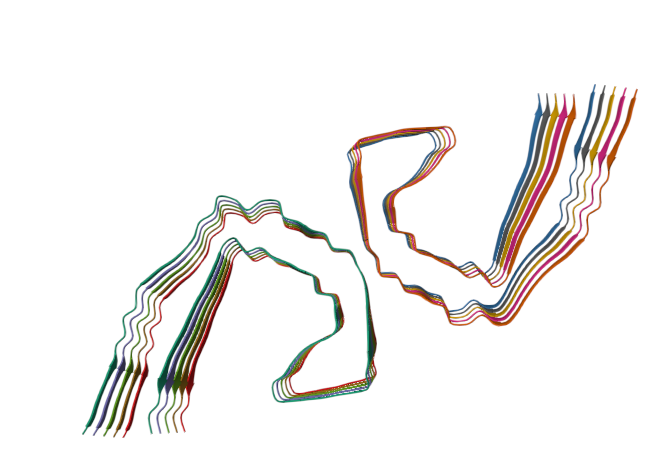 |
| 4. | Amyloid | 5txd | 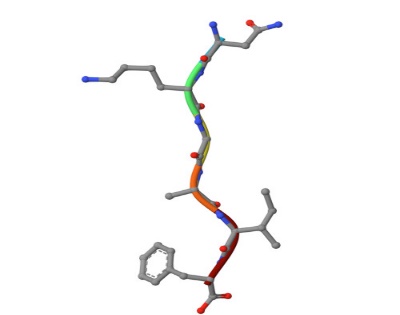 |

**T2: Auto dock vina results of different ligands with Sortilin receptor and 3 drugs.**

| **Sr.No.** | **Molecules** | **Binding energy**  **(kcal/mol)** | **Ligand efficiency** | **Inhibition constant (µM)** | **Electrostatic**  **Energy** | **Total Internal energy** | **Torsional energy**  **(kcal/mol)** | **Unbound energy**  **(kcal/mol)** | **Ref RMS** | **Amino acid**  **Residue** |
| --- | --- | --- | --- | --- | --- | --- | --- | --- | --- | --- |
|  | 4-tert-Amylphenol | -6.28 | -0.52 | 25.01 | -0.17 | -0.29 | 0.89 | -0.29 | 51.75 | ARG292: HH 21  TYR318: HN |
|  | Allicin | -4.75 | -0.53 | 330.59 | -0.25 | -0.2 | 1.49 | -0.2 | 52.2 | SER283: HG 1  ARG 292: HE 1 |
|  | Apigenin | -7.33 | -0.37 | 4.27 | -0.37 | -0.86 | -0.86 | -0.86 | 38.01 | ILE494: HN  SER541:HN |
|  | Astragalosides | -5.14 | -0.23 | 169.72 | -0.14 | -1.28 | 2.98 | -1.28 | 43.07 | TRP83:HN 1  SER541:HN 1 |
|  | Berberine | -7.24 | -0.29 | 4.95 | -0.09 | -0.35 | 0.6 | -0.35 | 49.49 | MET139: HN  TYR189: HN |
|  | Cannabidiol | -5.4 | -0.23 | 110.53 | -0.08 | -1.32 | 2.39 | -1.32 | 50.07 | TRP228: HN |
|  | Curcumin | -6.38 | -0.24 | 21.14 | -0.06 | -1.99 | 2.39 | -1.99 | 34.2 | SER432: HN  SER447:HN  GLY544:HN |
|  | Cyanidin | -5.94 | -0.28 | -44.07 | -0.3 | -0.37 | 1.79 | -0.37 | 50.33 | ILE141:HN |
|  | Dronabinol | -7.91 | -0.34 | 1.59 | -0.17 | -0.36 | 1.49 | -0.36 | 49.12 | ARG292:HH21 1 |
|  | Galantamine | -6.98 | -0.33 | 7.58 | -1.81 | -0.74 | 0.6 | -0.74 | n/a | ARG403:HH21 |
|  | Gingkolide | -16.29 | -0.54 | 1.14 | -0.28 | -0.74 | 0.89 | -0.74 | 54.03 | ARG292:HH21 1  TYR318:HN1 |
|  | Ginsenosides | -8.07 | -0.25 | 1.22 | -0.14 | -1.69 | 1.79 | -1.69 | 48.14 | ILE141:HN1  MET139: O |
|  | Harman | -5.56 | -0.4 | 83.64 | 0.00 | 0.0 | 0.0 | 0.0 | 50.12 | ILE141:O |
|  | Hesperidin | -3.95 | -0.09 | 1.26 | -0.29 | -7.74 | 4.47 | -7.74 | 50.054 | TRP228:HN |
|  | Resveratrol | -5.61 | -0.33 | 77.12 | -0.25 | -0.27 | -0.27 | -0.27 | 37.95 | ILE494:HN 1 |
| **Drugs** | | | | | | | | | |  |
|  | Memantine | -6.9 | -0.53 | 8.83 | -0.0 | 0.69 | 0.6 | 0.69 | 31.7 | GLN400:O |
|  | Donepezil | -6.66 | -0.24 | 13.08 | -0.3 | -0.9 | 1.79 | -0.91 | n/a | TYR189:O |
|  | Rivastigmine | -5.61 | -0.33 | 77.12 | -0.25 | -0.27 | 1.49 | -0.27 | 37.95 | ILE494:HN |

**T3: Autodock via results of different 13 ligands with Clustirin receptor and 3 drugs.**

| **Sr. No.** | **Molecules** | **Binding energy (kcl/mol)** | **Ligand efficiency** | **Inhabitation constant (Um)** | **Electrostatic energy** | **Total internal energy** | **Torsional energy(kcal/mol)** | **Unbounded energy(kcl/mol)** | **Ref RMS** | **Amino acid residue** |
| --- | --- | --- | --- | --- | --- | --- | --- | --- | --- | --- |
|  | 4-tert-Amylphenol | -3.99 | -0.33 | 1.19 | -0.08 | -0.27 | 0.89 | -0.27 | 26.38 | GLN349:HE22 |
|  | Allicin | -3.93 | -0.44 | 1.32 | 0.02 | -0.2 | 1.49 | -0.2 | 36.19 | SER184:HG |
|  | Apigenin | -5.51 | -0.28 | 91.5 | -0.12 | -0.88 | 1.19 | -0.88 | 33.98 | LYS351:HZ2  GLN361:HE21 |
|  | Astragalosides | -3.89 | -0.18 | 1.42 | -0.22 | -1.63 | 2.98 | -1.63 | 33.36 | LYS351:HZ2  NAG503:H2 |
|  | Berbirine | -6.46 | -0.26 | 18.27 | -0.03 | -0.29 | 0.6 | -0.29 | 19.72 | ASN291:HD2 |
|  | Cannabidiol | -3.56 | -0.15 | 2.45 | -0.08 | -1.5 | 2.39 | -1.5 | 32.77 | ARG 292: HE 1 |
|  | Curcumin | -4.01 | -0.15 | 1.16 | -0.24 | -1.88 | 2.39 | -1.88 | 44.07 | GLU364:OE1 |
|  | Cyanidin | -3.92 | -0.19 | 1.34 | -0.02 | -1.41 | 1.79 | -1.41 | 19.01 | No hydrogen bond |
|  | Dronabinol | -5.94 | -0.26 | 44.46 | -0.04 | -0.64 | 1.49 | -0.64 | 64.23 | ASN43:OD1 |
|  | Galantamine | -5.53 | -0.26 | 88.04 | -0.75 | -0.76 | 0.6 | -0.76 | 38.67 | GLN255:HE22 |
|  | Gingkolide | -13.98 | -0.47 | 56.33 | -0.09 | -0.74 | 0.89 | -0.74 | 56.75 | HIS205:HD1 |
|  | Ginsenosides | -8.02 | -0.25 | 1.33 | -0.11 | -1.6 | 1.79 | -1.6 | 61.72 | PHE240:HN, |
|  | Harman | -5.52 | -0.39 | 90.4 | -0.17 | 0.0 | 0.0 | 0.0 | 21.05 | GLU287:OE2 |
|  | Hesperidin | -5.42 | -0.13 | 106.76 | -0.39 | -6.12 | 4.47 | -6.12 | 19.54 | SER161:O, THR 283:O, GLU 287: OE1, THR293:HG1, |
|  | Resveratrol | -6.34 | -0.37 | 22.62 | -0.1 | -0.26 | 1.49 | -0.26 | 60.78 | NAG504:04, PRO212:0, ALA373:0 |
| Drugs | | | | | | | | | |  |
| 1 | Donepezil | -5.65 | -0.2 | 72.53 | -0.86 | -0.86 | 1.79 | -0.86 | 38.88 | GLY378:0 |
| 2 | Memantine | -5.4 | -0.24 | 110.12 | -0.46 | 0.07 | 0.3 | 0.07 | 27.96 | No hydrogen bond |
| 3 | Rivastigmine | -4.93 | -0.27 | 242.2 | -1.07 | -0.73 | 1.49 | -0.73 | 19.33 | THR293:HG1 |

**T4. Autodock vina results of different ligands with Amyloid peptide.**

| **Sr.No.** | **Molecule** | **Binding Energy (kcal/m0l)** | **Ligand Efficency** | **Inhibition constant** | **Electrostatic energy** | **Total internal energy** | **Torsional energy (kcal/mol)** | **Un bounded energy (kcal/mol)** | **ref RMS** | **Amino acid residue** |
| --- | --- | --- | --- | --- | --- | --- | --- | --- | --- | --- |
|  | 4 -tert-Amylphenol | -2.5 | -0.21 | 14.8 | -0.04 | 0.89 | 0.89 | -0.25 | 6.1 | ILE5::0, ILE5:HN |
|  | Allicin | -1.43 | -0.16 | 89.9 | -0.04 | -0.18 | 1.49 | -0.18 | 6.47 | GLY3:HN |
|  | Apigenin | -2.42 | -0.12 | 16.86 | -0.19 | -0.89 | 1.19 | -0.89 | 5.8 | LYS2:0, LYS2:HZ2 |
|  | Astragalcociides | -1.37 | -0.06 | 98.21 | 0.07 | -1.55 | 2.98 | -1.55 | 5.77 | GLY3:HN |
|  | Berbirine | -3.57 | -0.14 | 2.43 | -0.06 | -0.33 | 0.6 | -0.33 | 5.89 | ILE5:0 |
|  | Cannabidiol | -2.03 | -0.09 | 32.57 | -0.07 | -1.38 | 2.39 | -1.43 | 4.35 | GLY3:0 |
|  | Curcumin | -2.17 | -0.08 | 25.65 | -0.12 | -2.0 | 2.39 | -2.0 | 5.63 | ASN1:HD22 |
|  | Cyanidin | -3.94 | -0.19 | 1.3 | -0.19 | -1.26 | -1.79 | -1.26 | 4.58 | ILE5:0  ILE5:HN  ASN1:0 |
|  | Dronabinol | -3.53 | -0.15 | 2.6 | -0.24 | -0.42 | 1.49 | -0.42 | 8.19 | LYS2:HZ2 |
|  | Galantamine | -3.21 | -0.15 | 4.46 | -0.16 | -0.73 | 0.6 | -0.73 | 8.64 | No hydrogen bond |
|  | Ginkgolide | -8.4 | -0.28 | 700.13 | -0.02 | -0.73 | -0.89 | -0.73 | 7.0 | No hydrogen bond |
|  | Ginsenosides | -4.36 | -0.14 | 639.84 | -0.33 | -0.94 | 1.79 | -0.94 | 5.74 | ALA4:0, ALA4:HN, LYS2:HZ2 |
|  | Harman | -3.7 | -0.26 | 1.93 | -0.06 | 0.0 | 0.0 | 0.0 | 4.66 | GLY3:0 |
|  | Hesperidin | -2.37 | -0.06 | 18.35 | -0.31 | -5.18 | 4.47 | -5.18 | 5.8 | LYS2:HZ2, GLY3:HN, GLY3:0 |
|  | Resveratrol | -2.2 | -0.13 | 24.41 | -0.24 | -0.26 | 1.49 | -0.26 | 4.39 | LYS2:HZ1, GLY3:HN, GLY3:0 |
|  | Dopenzil | -3.35 | -0.12 | 3.48 | -0.04 | -1.27 | 1.79 | -1.27 | 5.4 | ILE5:HN |
|  | Memantine | -3.56 | -0.27 | 2.46 | 0.0 | 0.7 | 0.3 | 0.7 | 6.71 | ILE5:0 |
|  | Rivastigmine | -2.16 | -0.12 | 26.21 | 0.07 | -0.84 | 1.49 | -0.84 | 7.22 | GLY3:HN |

**T5. Autodock vina results of different ligands with tau proteins.**

| **Sr.**  **No** | **Molecules** | **Binding**  **Energy (kcal/m0l)** | **Ligand effiency** | | **Inhibition constant** | | **Electrostatic energy** | **Total internal energy** | **Torsional**  **energy**  **(kcal/mol)** | **Un bounded energy(kcal/mol)** | **ref RMS** | **Amino acid residue** |
| --- | --- | --- | --- | --- | --- | --- | --- | --- | --- | --- | --- | --- |
|  | 4 -tert-Amylphenol | -3.52 | -0.29 | 2.65 | | -0.02 | | -0.26 | 0.89 | -0.26 | 301.08 | VAL339:HN |
|  | Allicin | -2.5 | -0.28 | 14.75 | | -0.05 | | -0.12 | 1.49 | -0.12 | 292.96 | VAL339:HN |
|  | Apigenin | -5.01 | -0.25 | 211.53 | | -0.13 | | -0.9 | 1.19 | -0.9 | 293.09 | GLY326:0  VAL363:0  ILE328:HN |
|  | Astragalcociides | -3.8 | -0.17 | 1.64 | | -0.03 | | -1.44 | 2.98 | 1.44 | 295.34 | GLY326:0 |
|  | Berberine | -5.33 | -0.21 | 124.0 | | -0.02 | | -0.34 | 0.6 | -0.34 | 315.74 | GLN351:HN |
|  | Cannabidiol | -3.46 | -0.15 | 2.89 | | -0.03 | | -1.46 | 2.39 | -1.46 | 269.48 | No hydrogen bond |
|  | Curcumin | -4.64 | -0.17 | 396.92 | | -0.17 | | -1.99 | 2.39 | -1.99 | 299.11 | VAL339:HN  LEU357:HN  SER341:HN  VAL337:0 |
|  | Cyanidin | -4.45 | -0.21 | 551.53 | | -0.03 | | -1.28 | 1.79 | -1.28 | 304.57 | ILE360:0 |
|  | Dronabinol | -4.78 | -0.21 | 310.89 | | -0.02 | | -0.25 | 1.49 | -0.25 | 288.21 | No hydrogen bond |
|  | Galantamine | -5.47 | -0.26 | 97.29 | | 0.0 | | -0.7 | 0.6 | -0.7 | 294.37 | VAL337:0  VAL339:HN |
|  | Ginkgolide | -10.63 | -0.35 | 16.16 | | -0.14 | | -0.73 | 0.89 | -0.73 | 295.52 | HIS329:HD1 |
|  | Ginsenosides | -6.28 | -0.2 | 24.74 | | -0.1 | | -0.78 | 1.79 | -0.78 | 301.95 | HIS329:0  PRO364:0 |
|  | Harman | -4.35 | -0.31 | 649.5 | | 0.02 | | 0.0 | 0.0 | 0.0 | 296.17 | ASN327:0D1 |
|  | Hesperidin | -3.76 | -0.09 | 1.74 | | -0.13 | | -6.89 | 4.47 | -6.89 | 291.1 | ASN359:0 |
|  | Resveratrol | -4.6 | -0.27 | 428.19 | | -0.26 | | -0.27 | 1.49 | -0.27 | 305.08 | ASP358:HN, ILE360:0, SER356:0G, LYS353:HZ3 |
| 1 | Dopenzil | -5.34 | -0.19 | 121.22 | | 0.98 | | -1.01 | 1.79 | -1.01 | 269.0 | No Hydrogen Bound |
| 2 | Memantine | -4.81 | -0.37 | 295.65 | | 0.0 | | 0.69 | 0.3 | 0.69 | 300.92 | ILE360:0 |
|  | Rivastigmine | -3.33 | -0.18 | 3.6 | | 0.04 | | -0.7 | 1.49 | -0.7 | 301.06 | VAL339:HN |


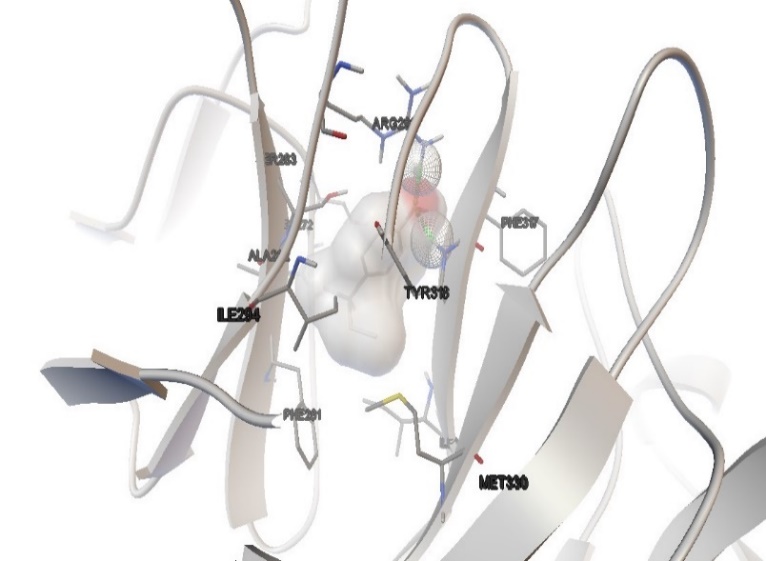

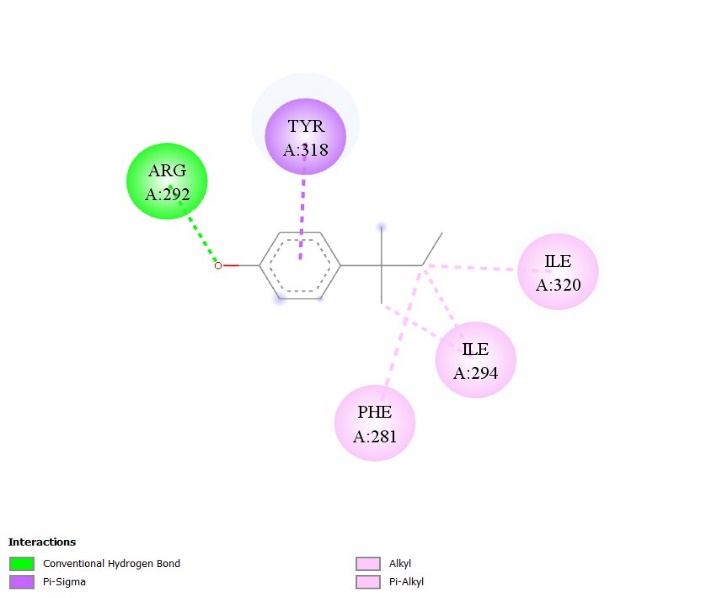

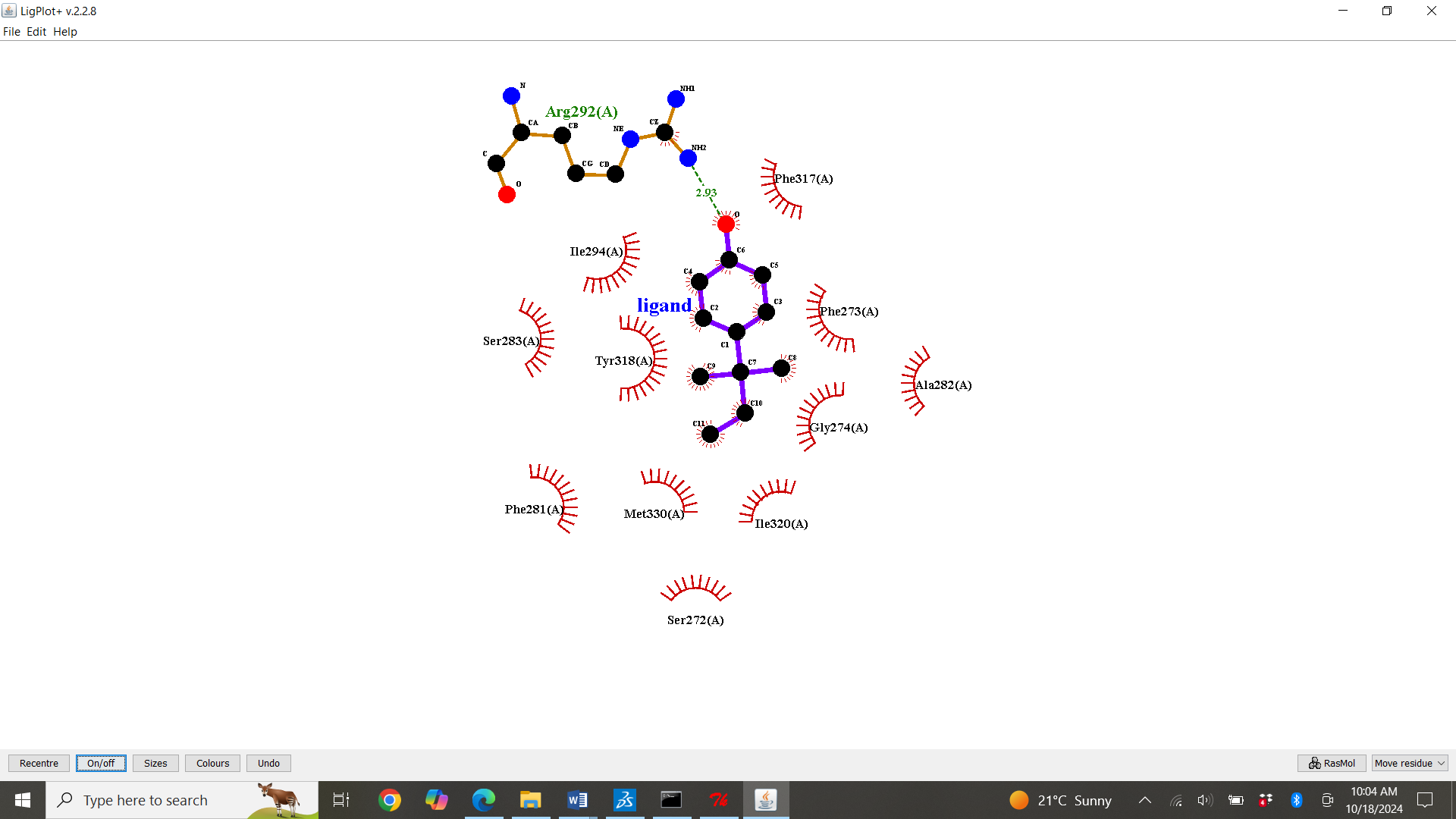


**S1: Molecular Docking, secondary structure interaction and ligplus analysis of 4 -tert-Amylphenol interactions with Sortilin.**
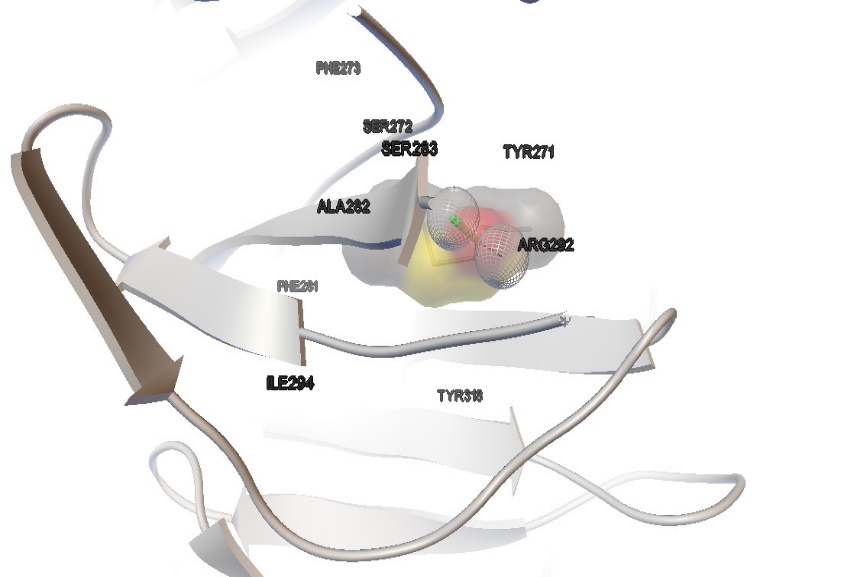

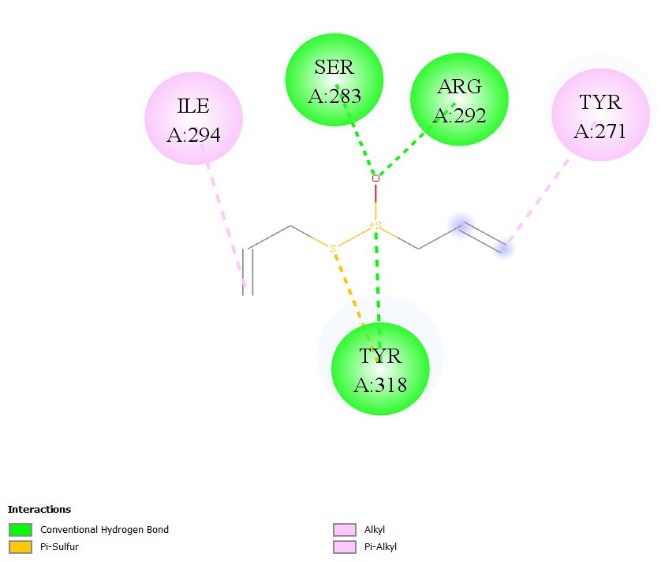

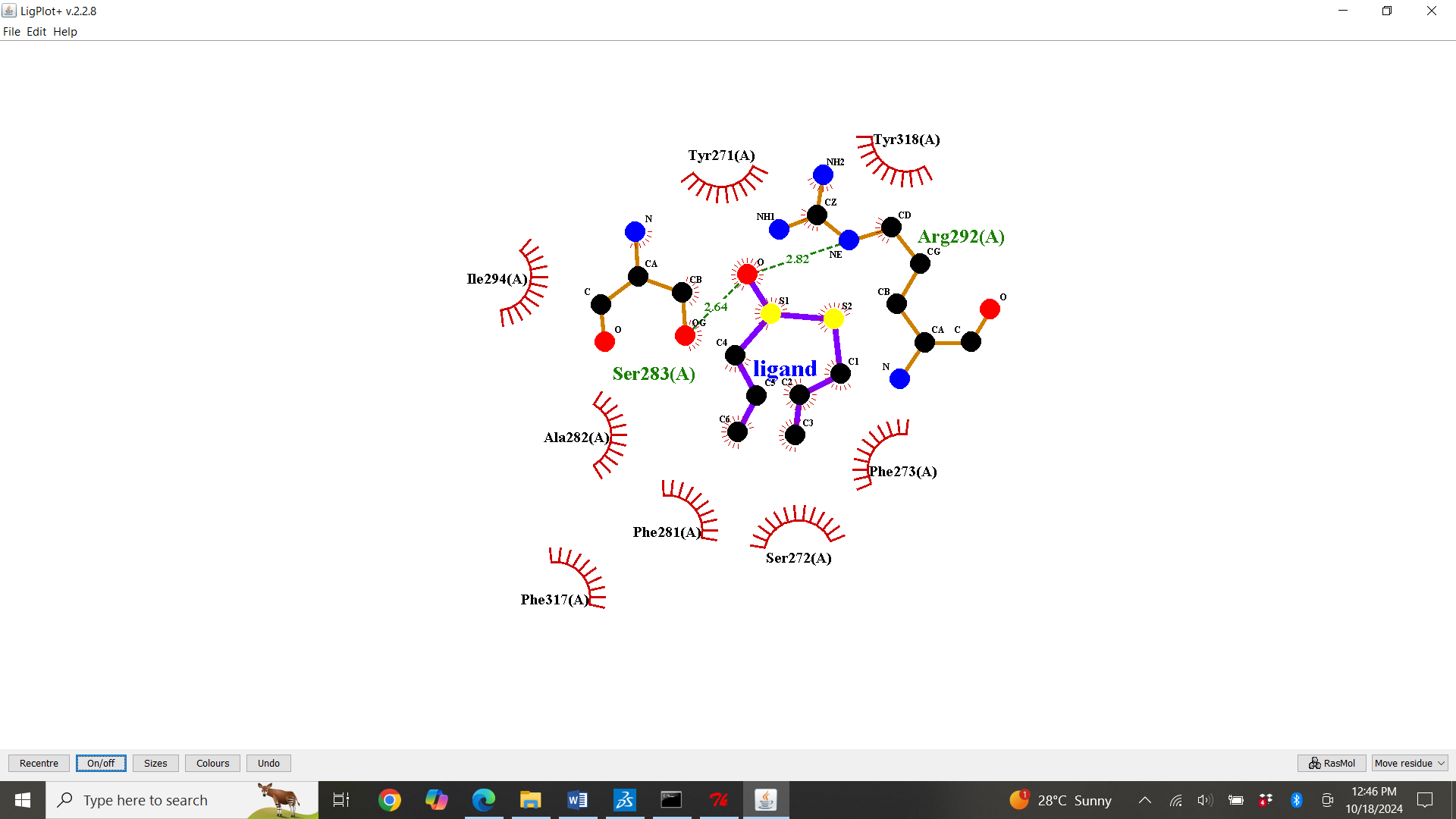


**S2: Molecular Docking, secondary structure interaction and ligplus analysis of Allicin interactions with Sortilin.**


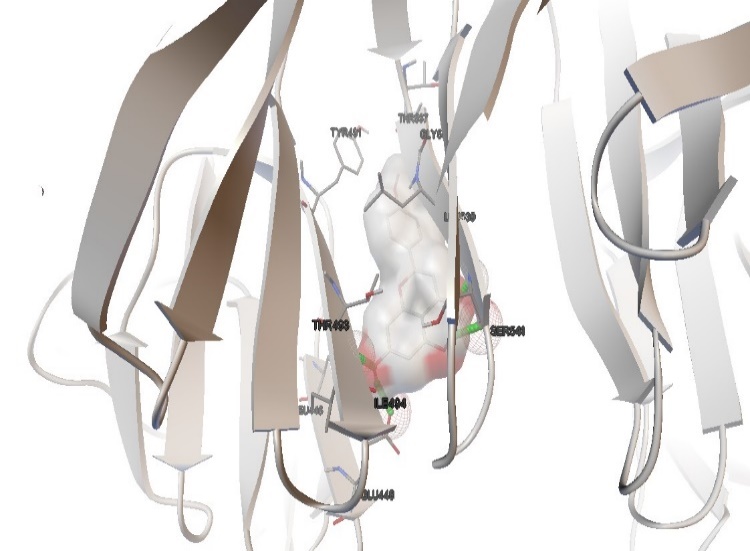

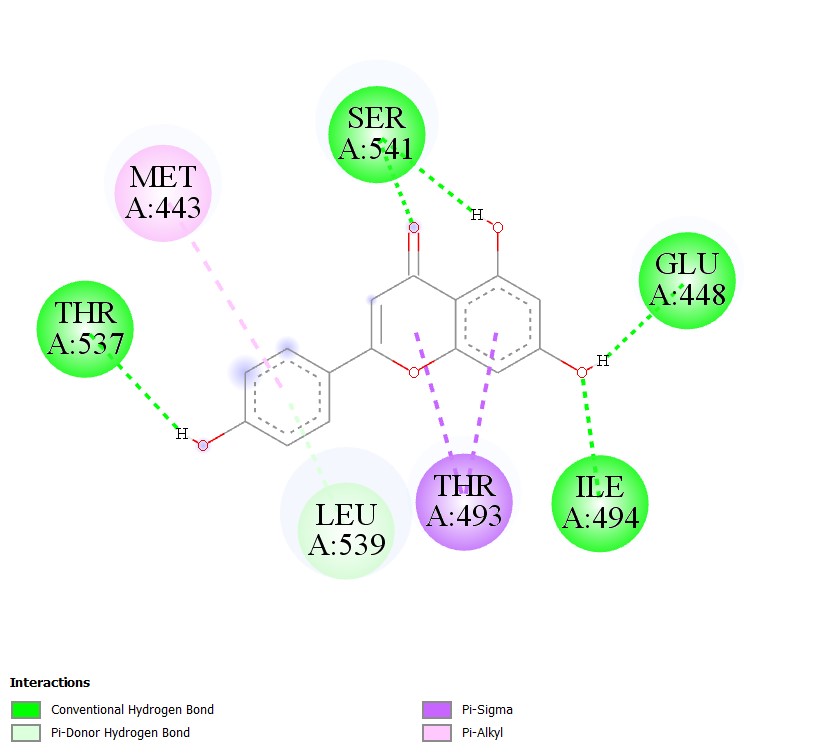

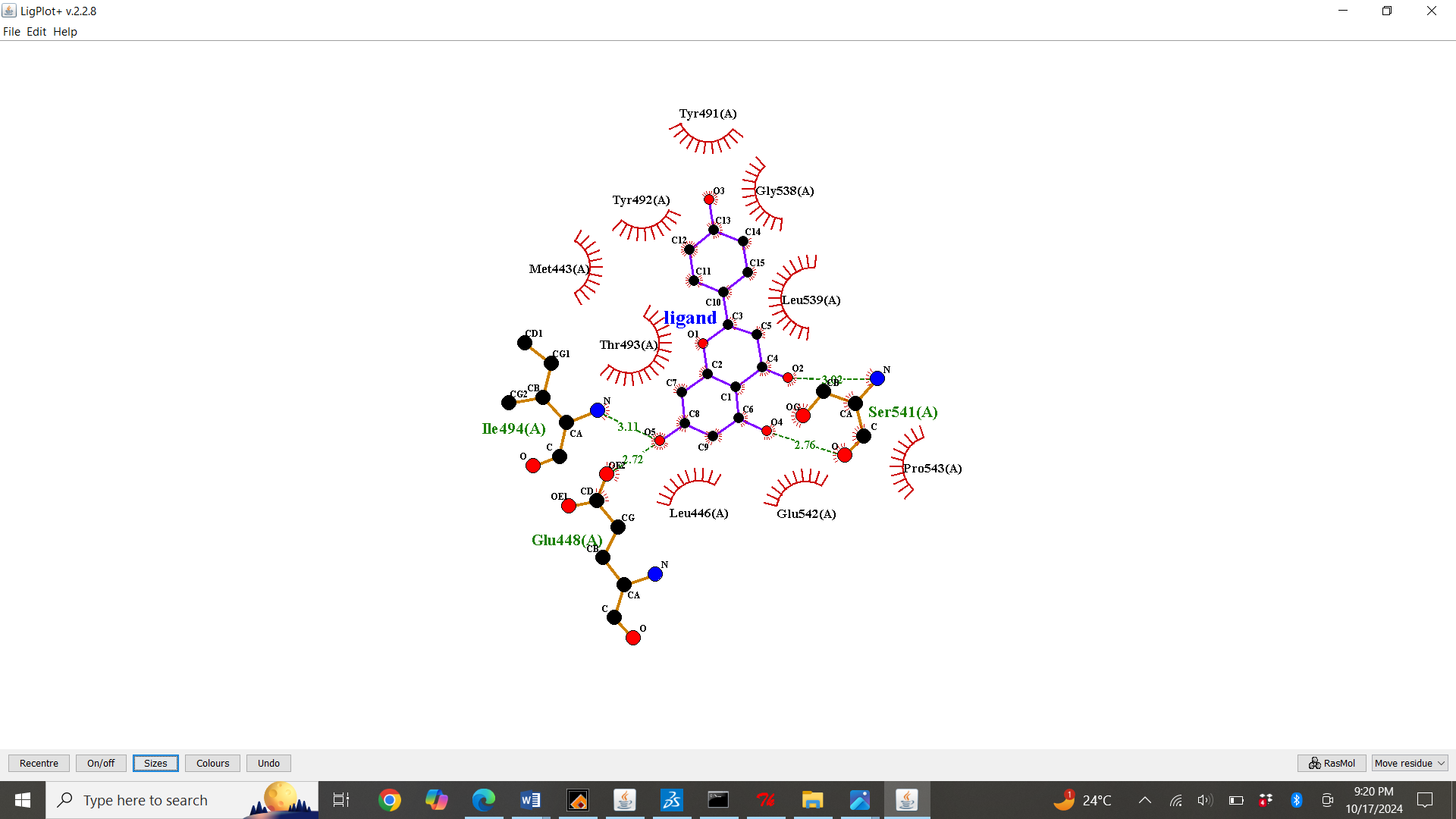


**S3: Molecular Docking, secondary structure interaction and ligplus analysis of Apigenin interactions with Sortilin.**


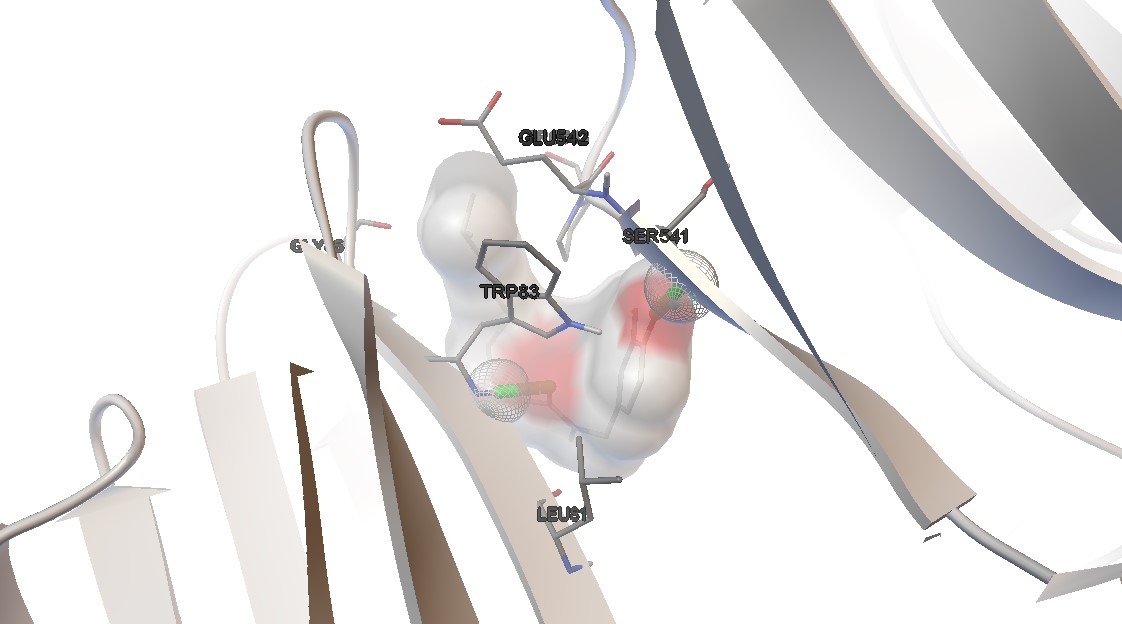

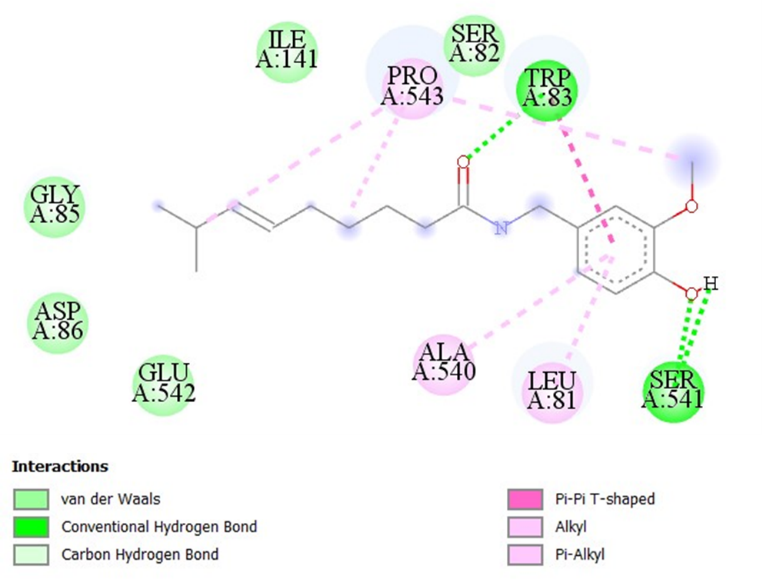

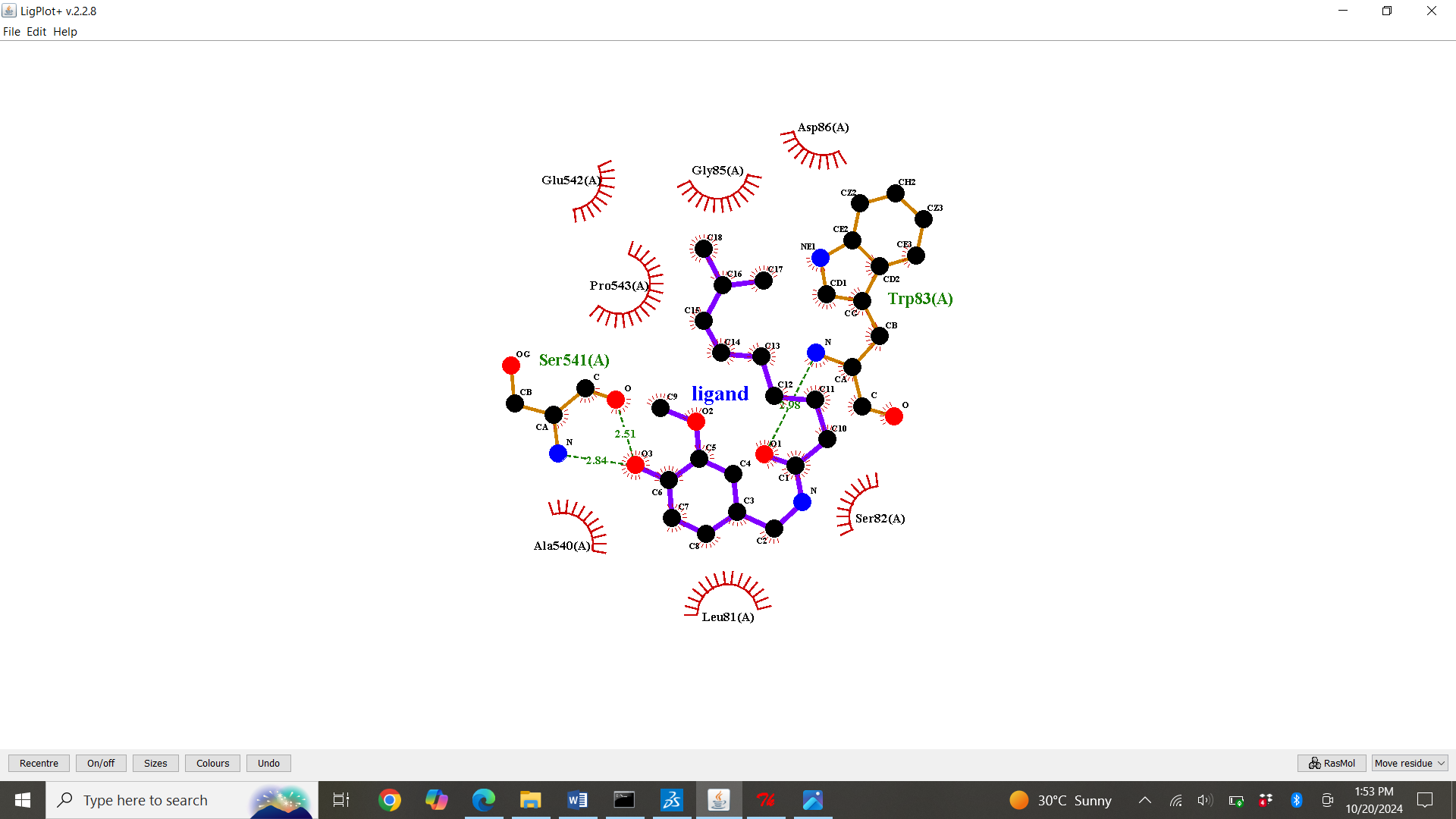


S**4: Molecular Docking, secondary structure interaction and ligplus Analysis of Astragalosides Interactions with Sortilin.**


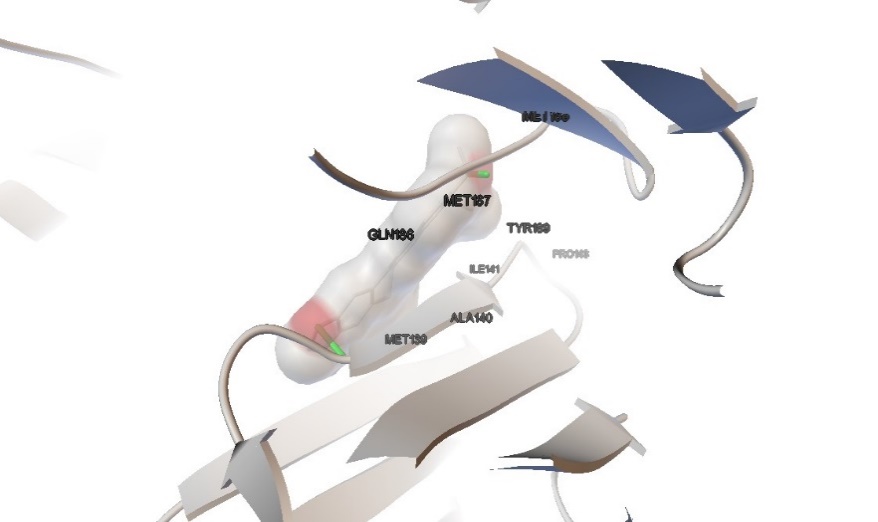

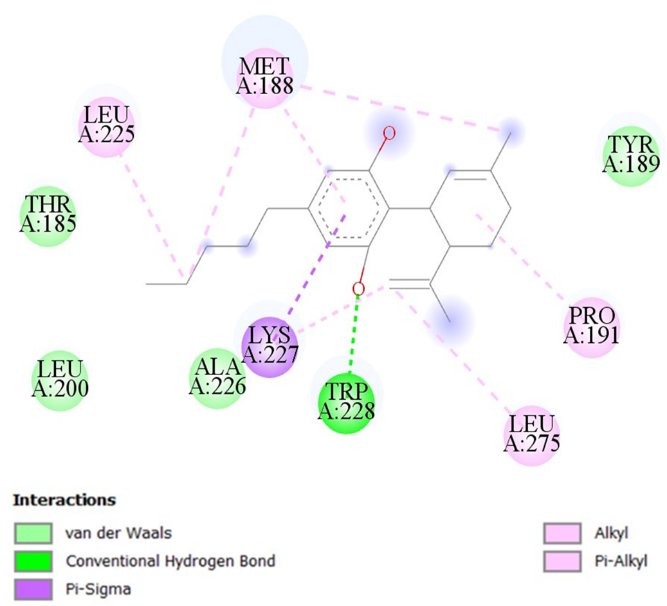

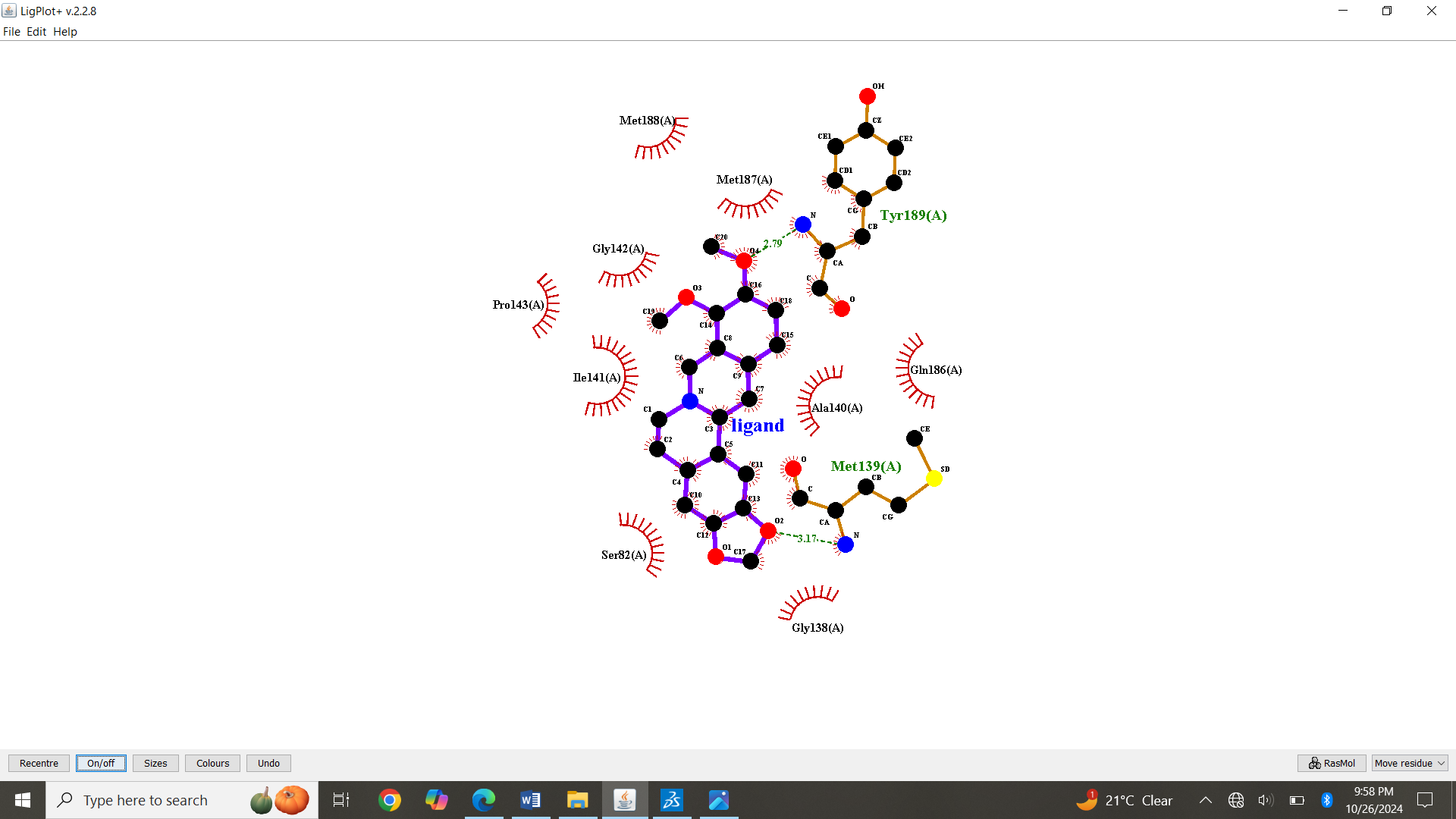


S**5: Molecular Docking, secondary structure interaction and ligplus analysis of Berberine interactions with Sortilin.**
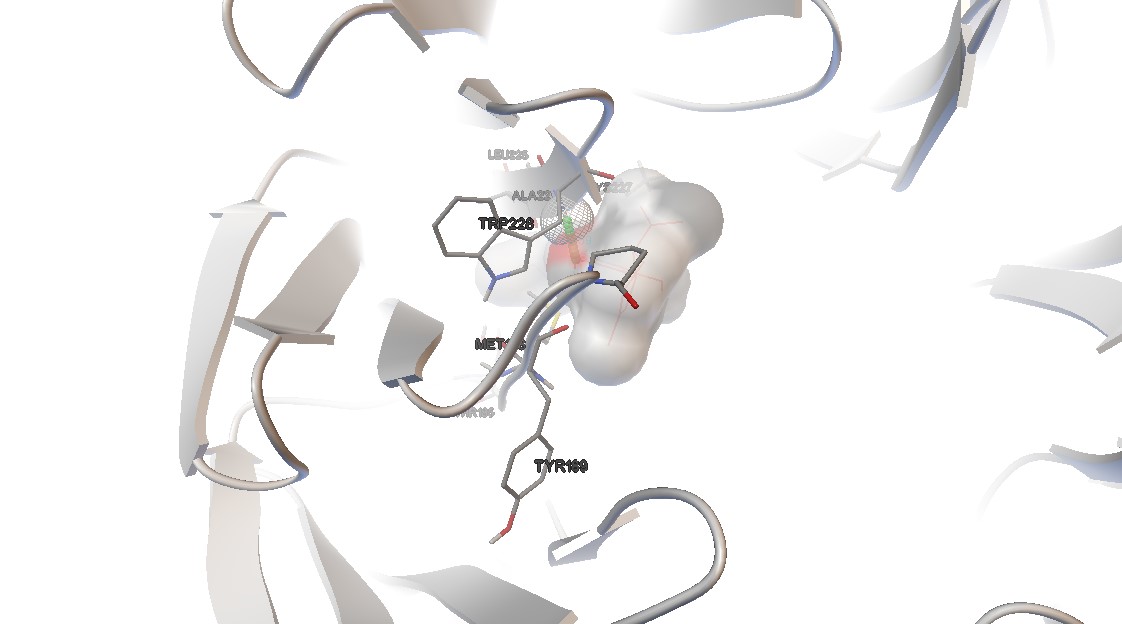

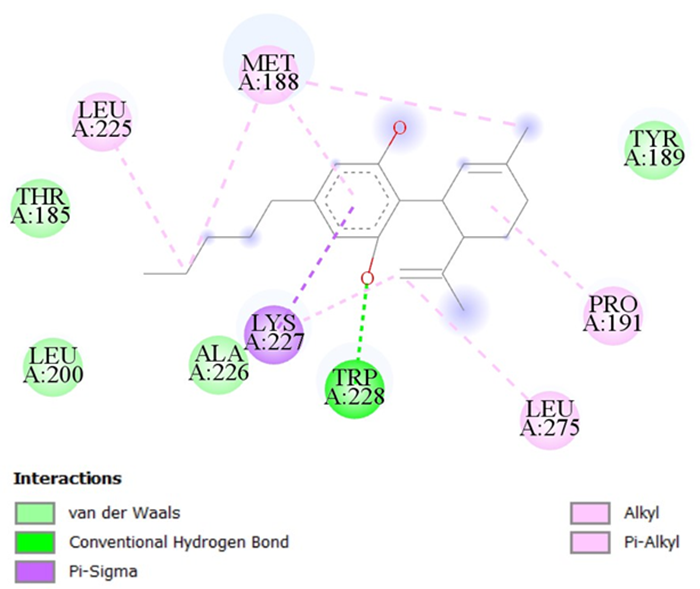

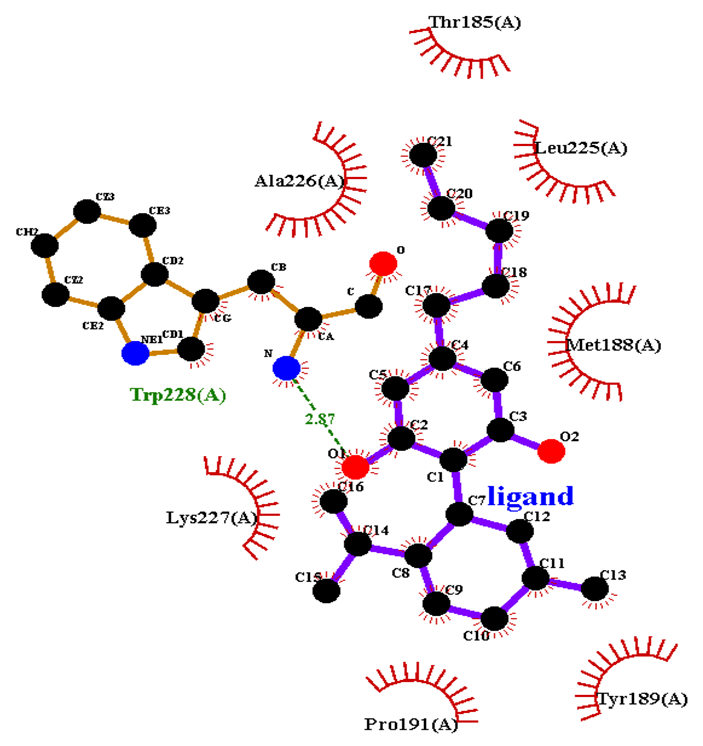


**S6: Molecular Docking, secondary structure interaction and ligplus analysis of Cannabidiol interactions with Sortilin.**


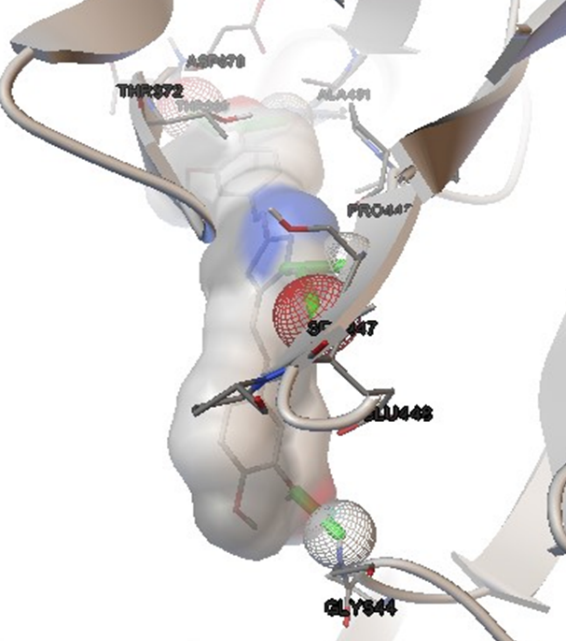

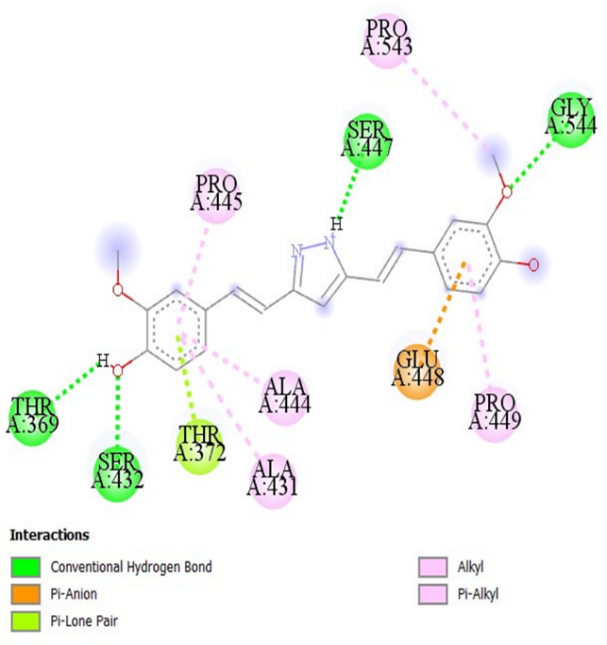

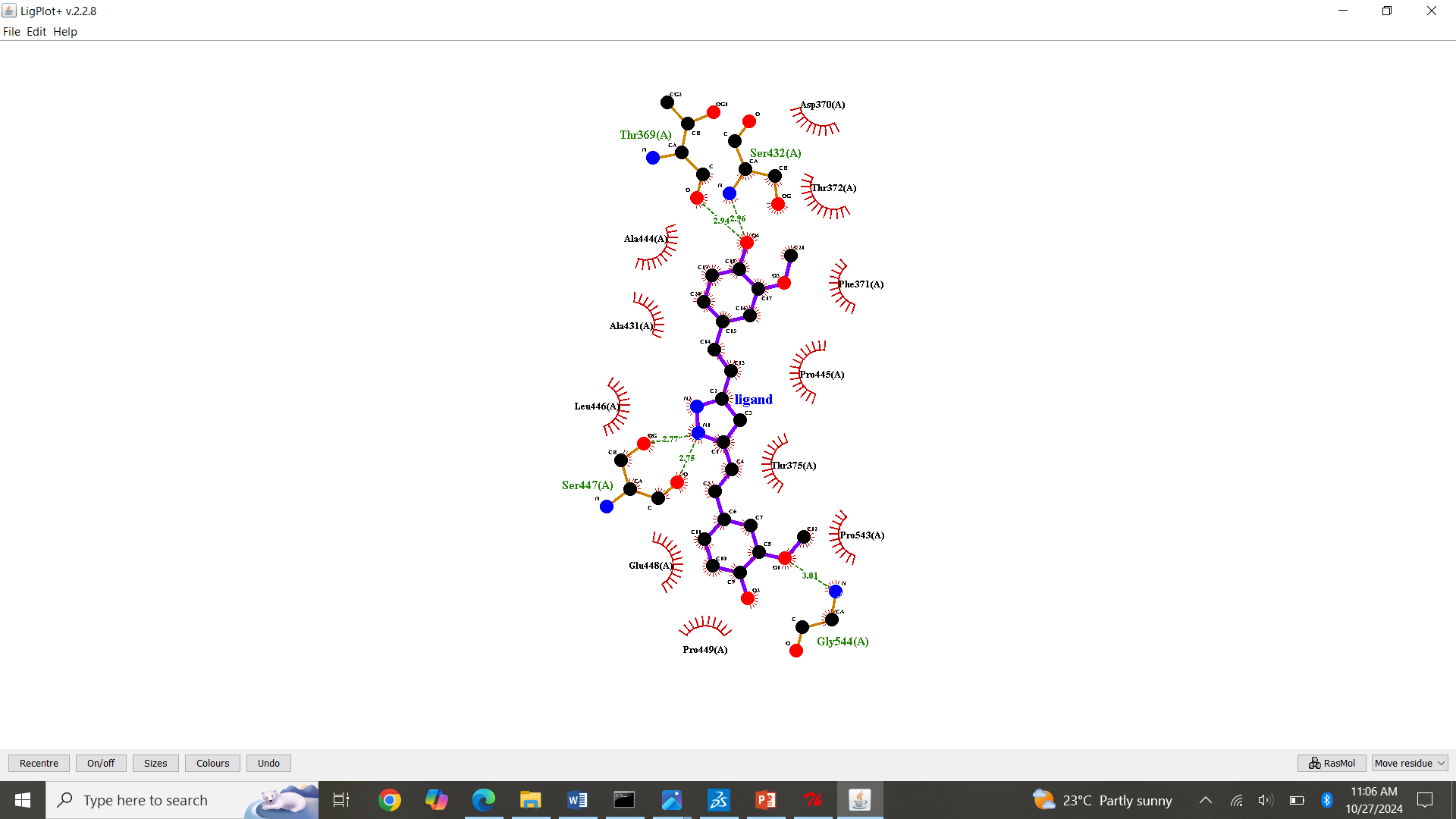


S**7: Molecular Docking, secondary structure interaction and ligplus analysis of Curcumin interactions with Sortilin.**
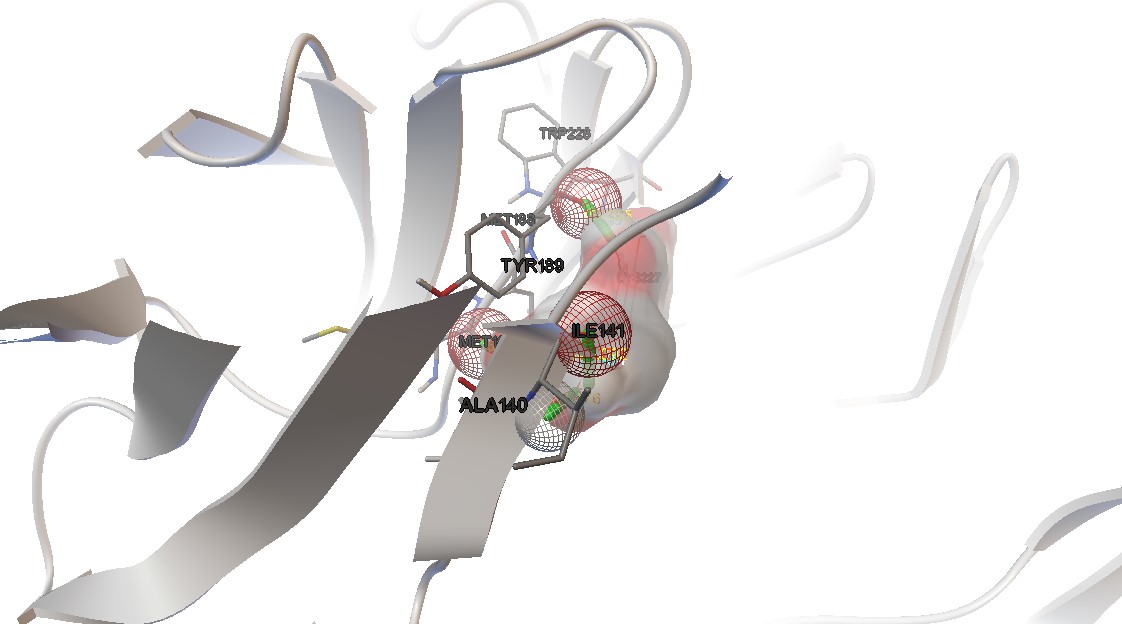

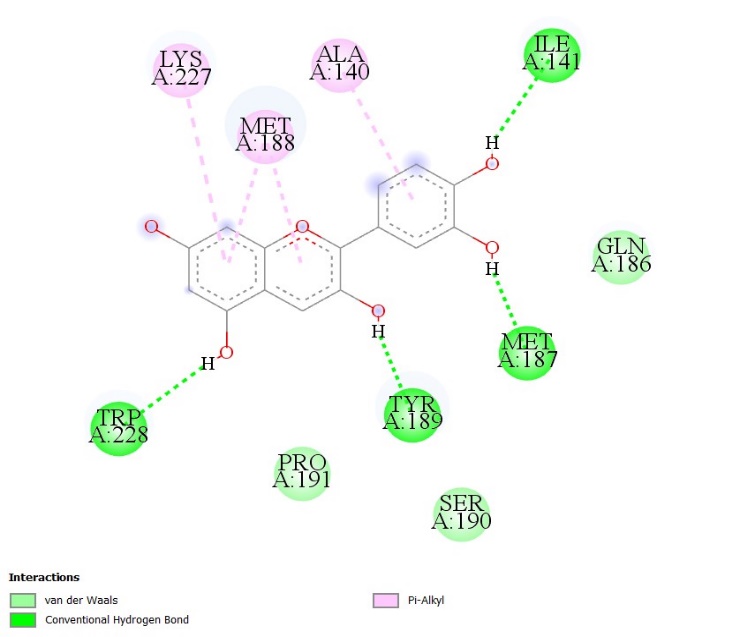

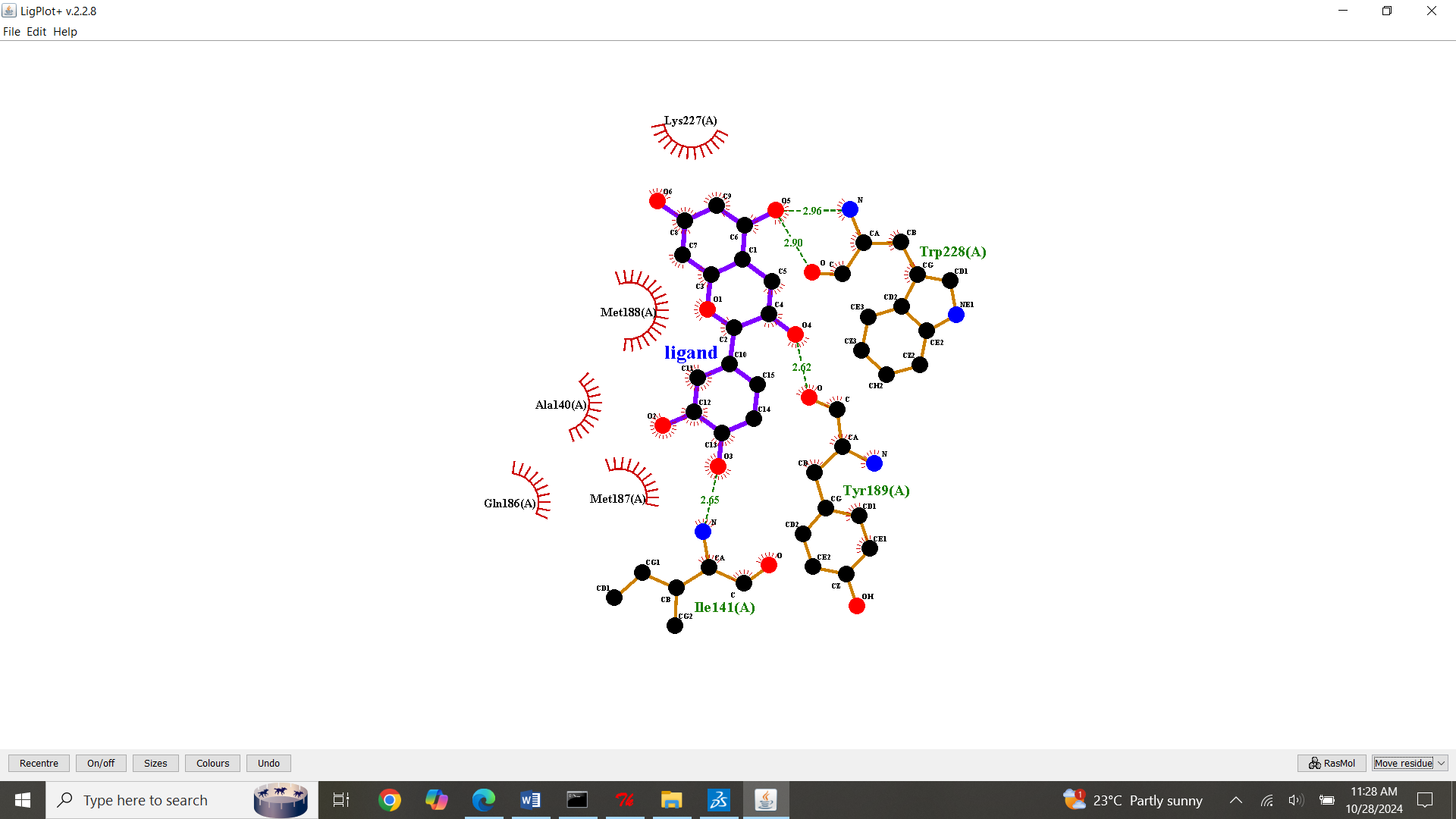


S**8: Molecular Docking, secondary structure interaction and ligplus analysis of Cyanidin interactions with Sortilin.**


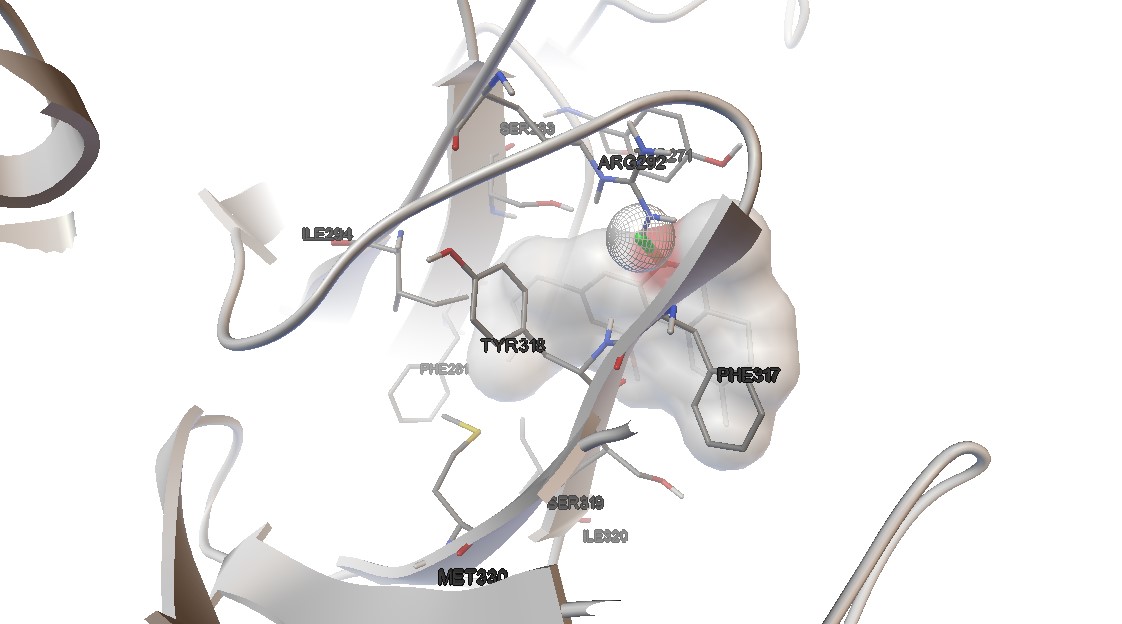

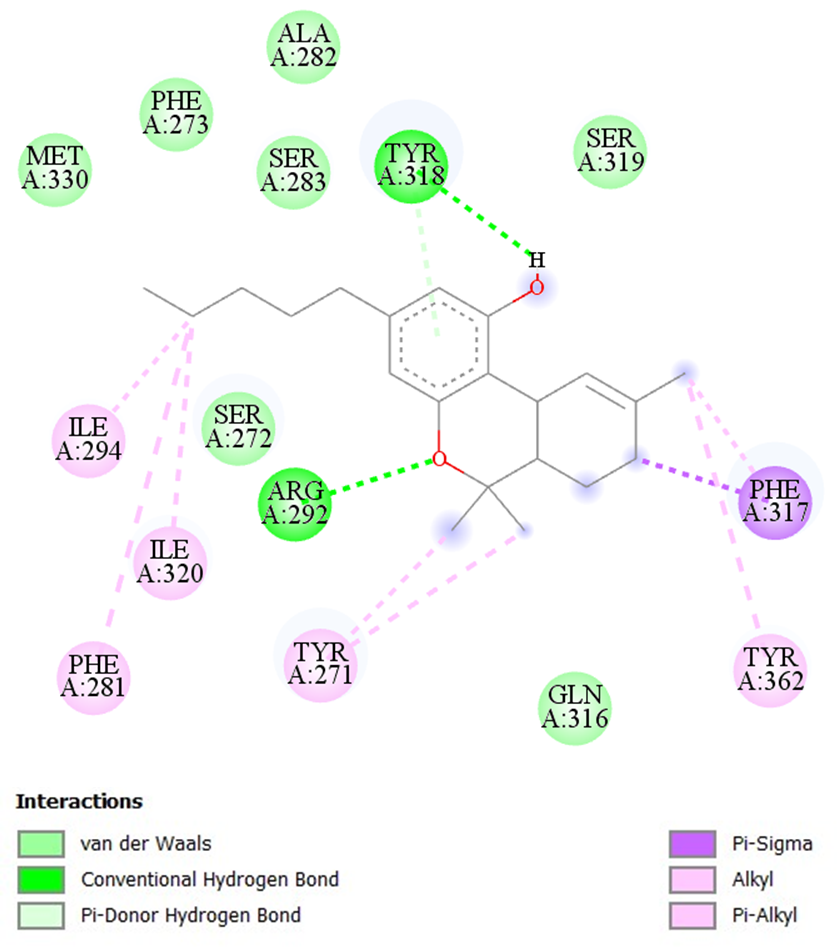

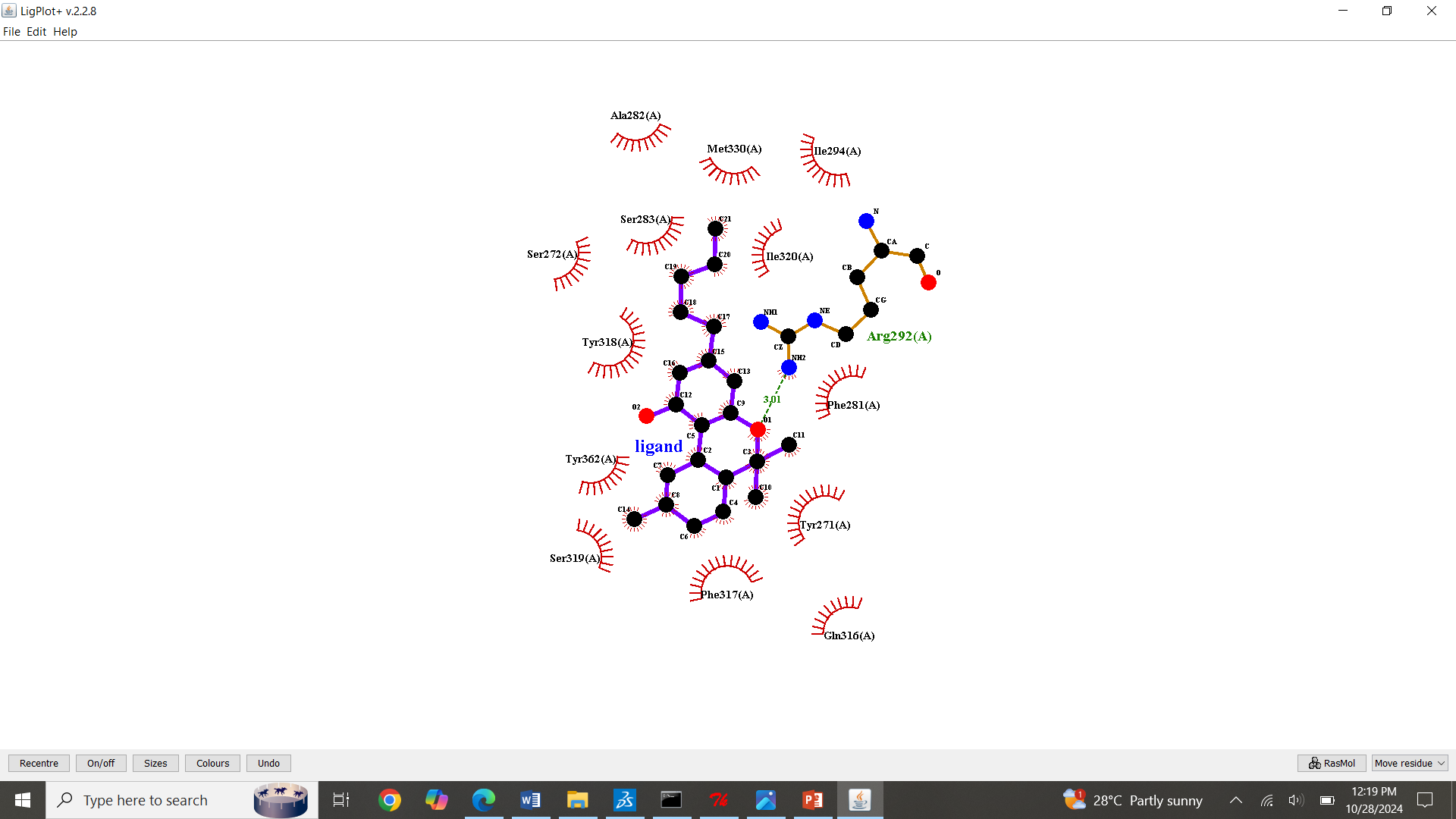


**S9: Molecular Docking, secondary structure interaction and ligplus analysis of Dronabinol interactions with Sortilin.**


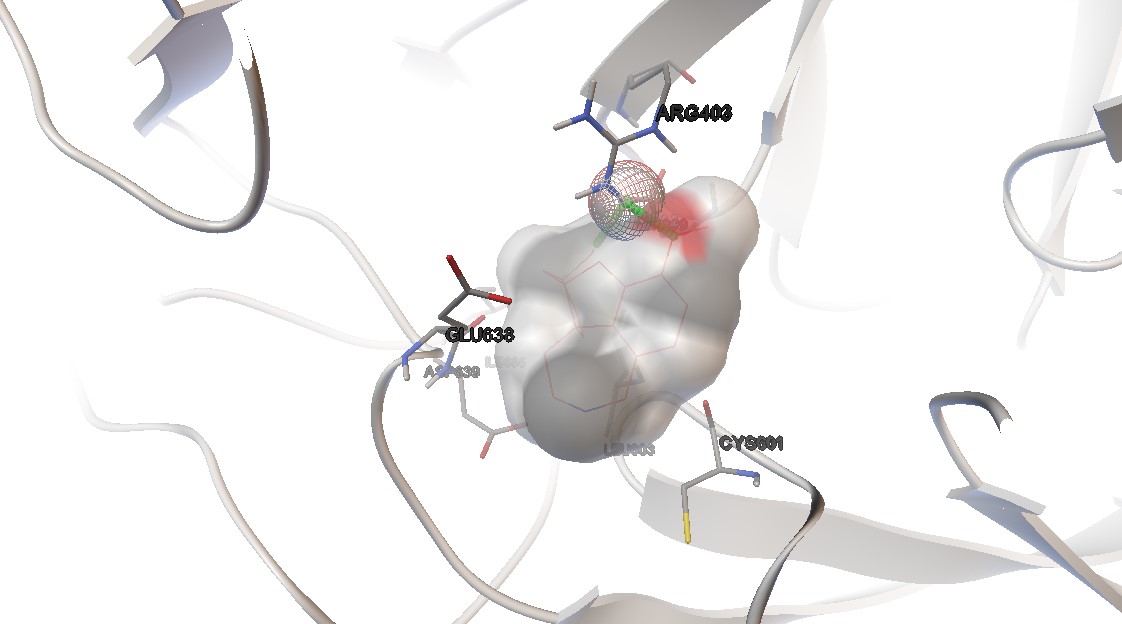

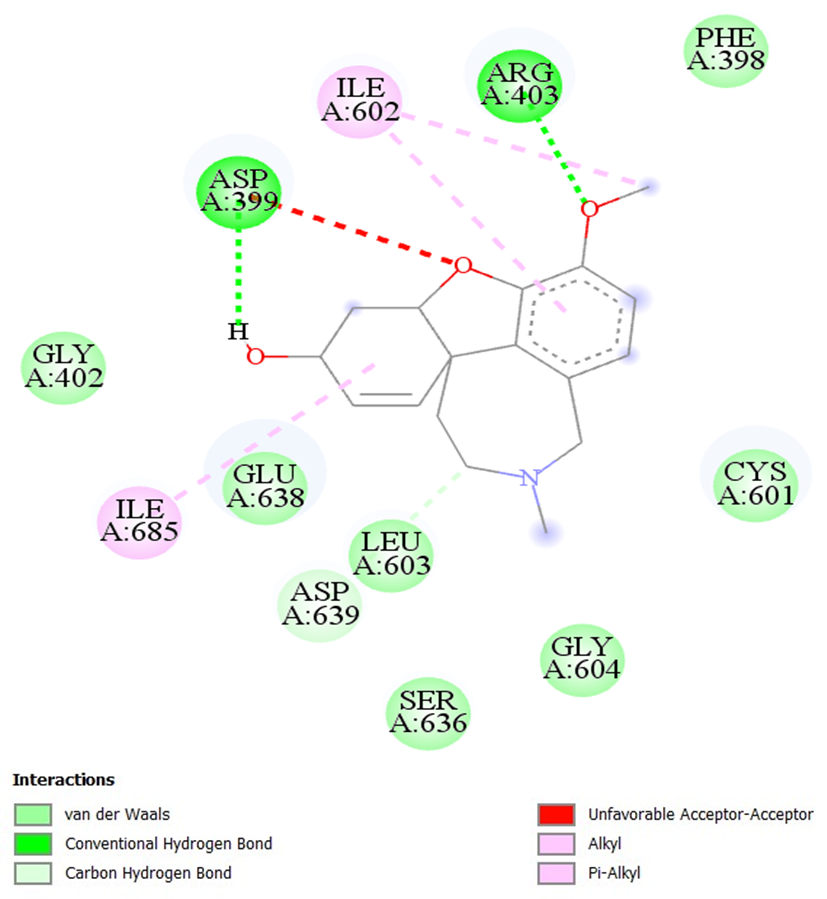

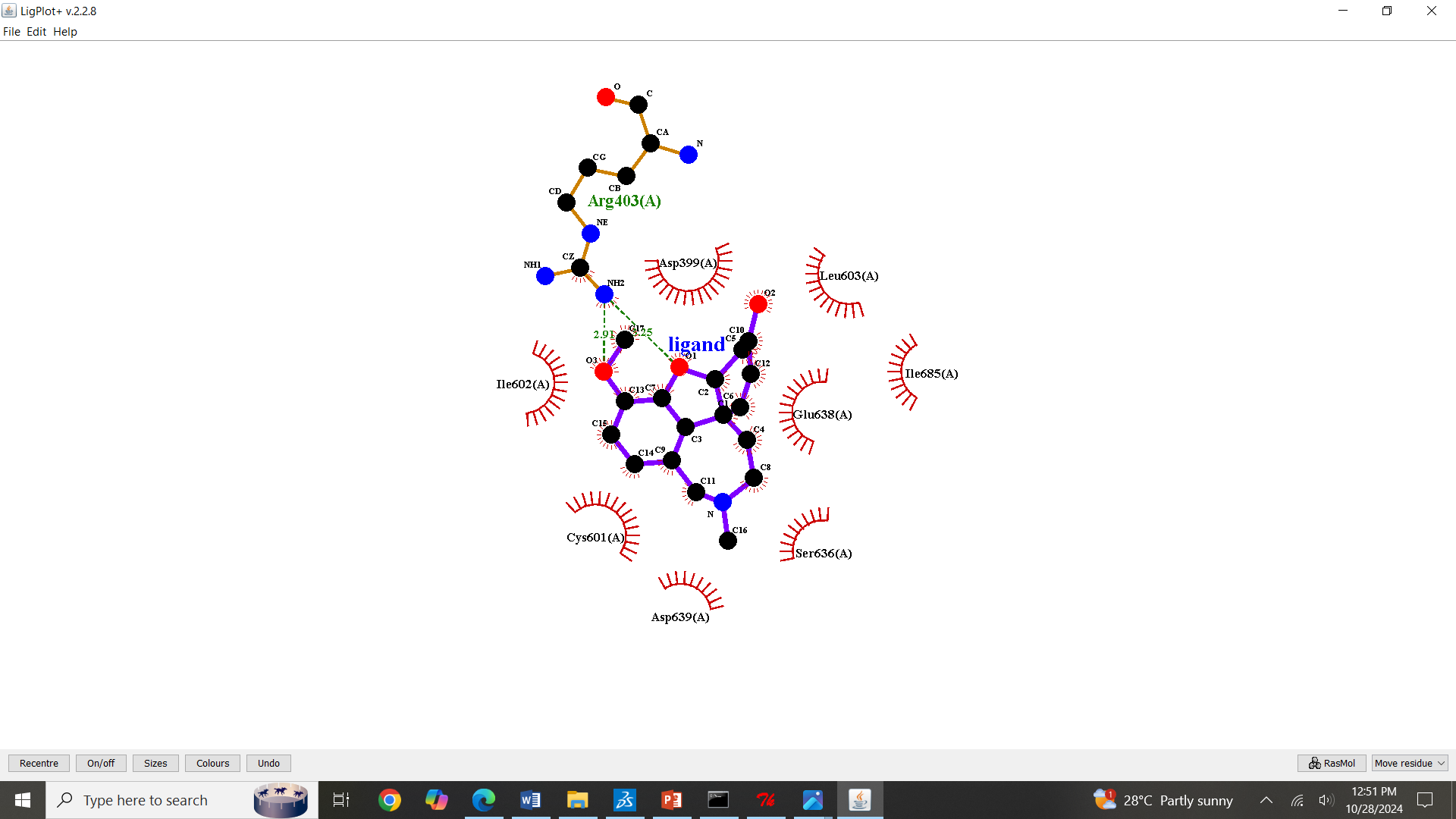


**S10: Molecular Docking, secondary structure interaction and ligplus analysis of Galantamine interactions with Sortilin.**


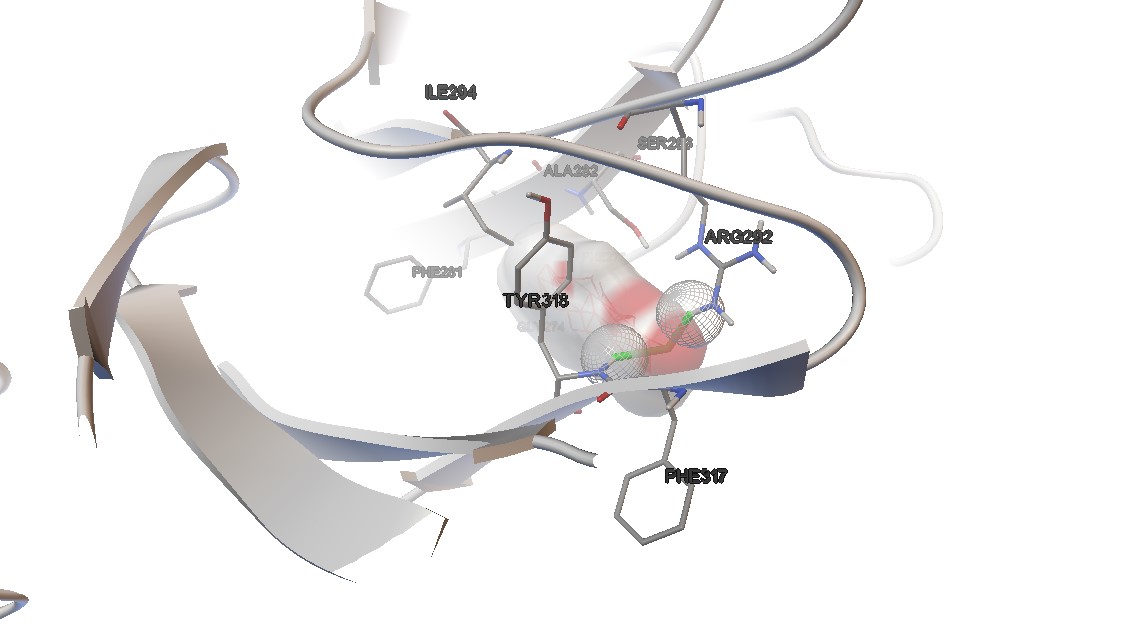

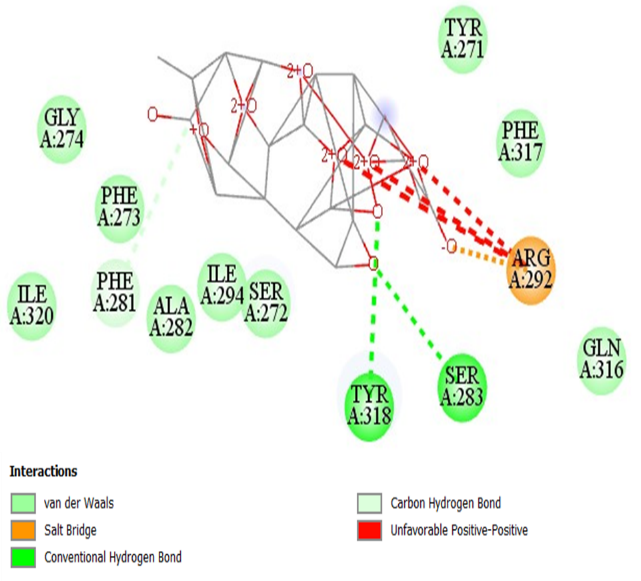

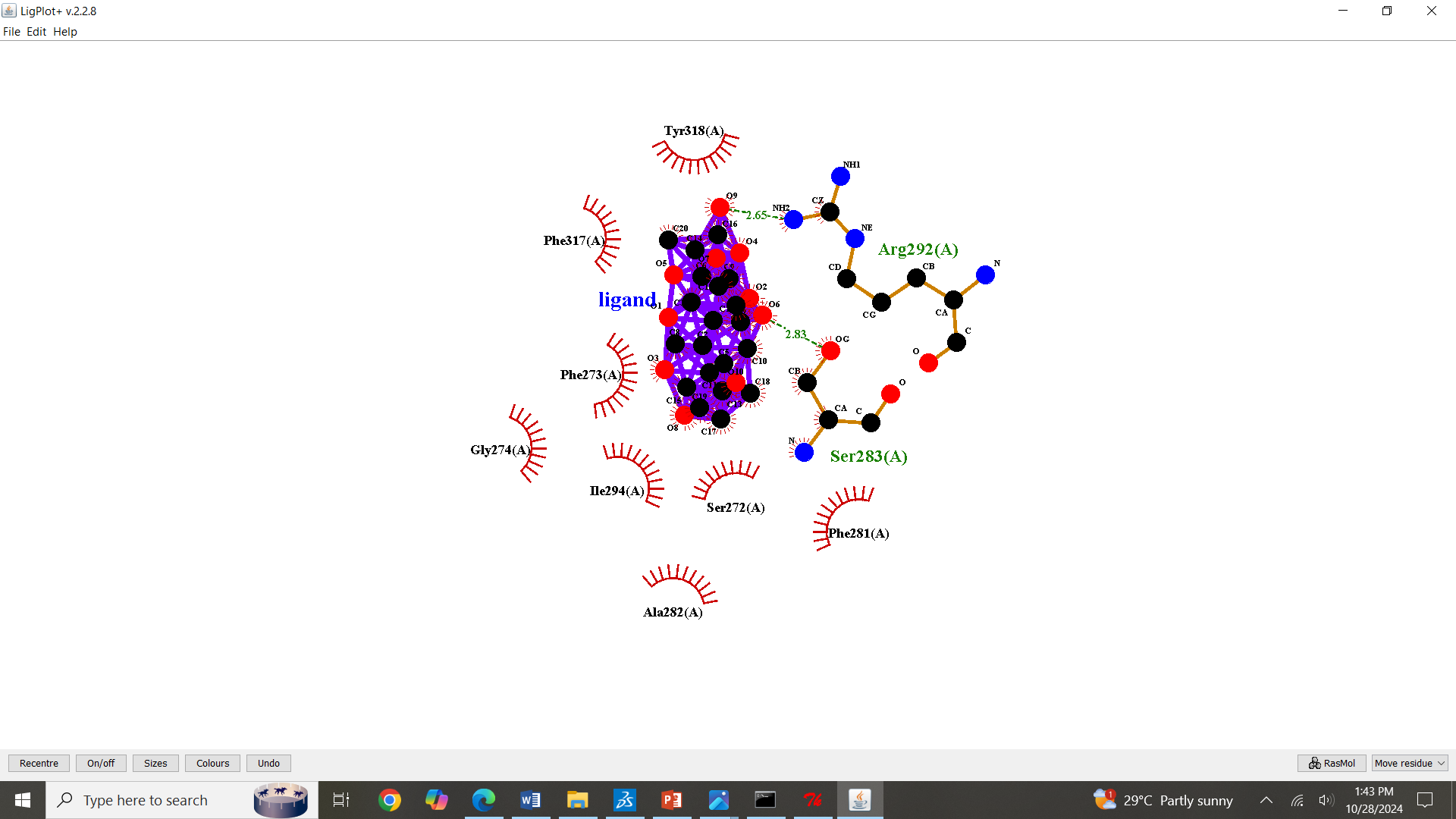


S**11: Molecular Docking, secondary structure interaction and ligplus analysis of Ginkolide interactions with Sortilin.**


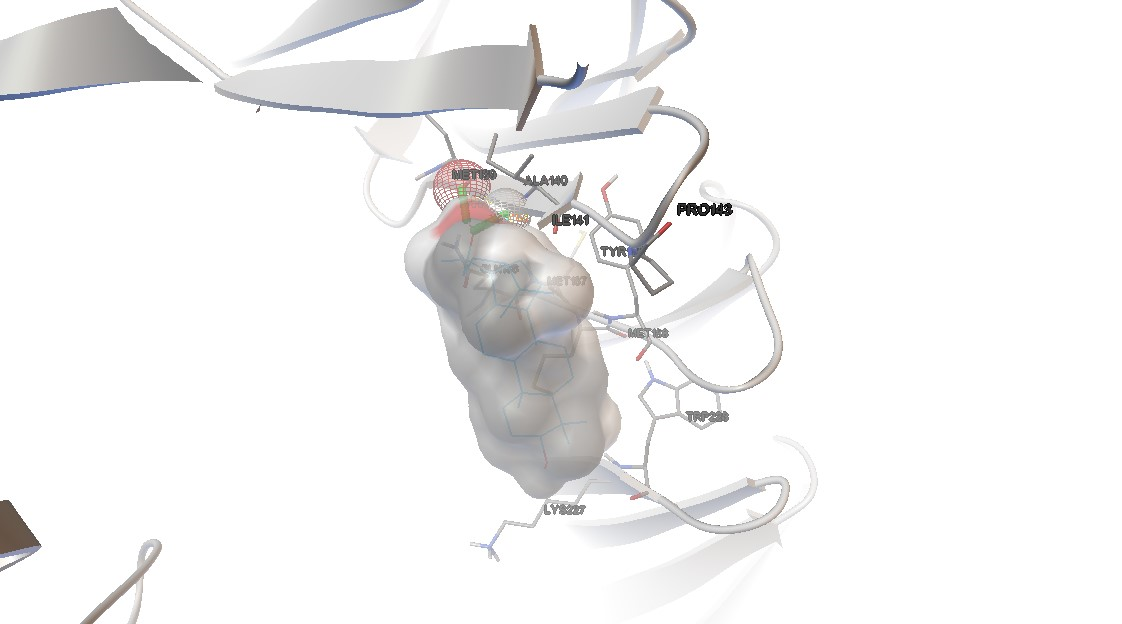
 **
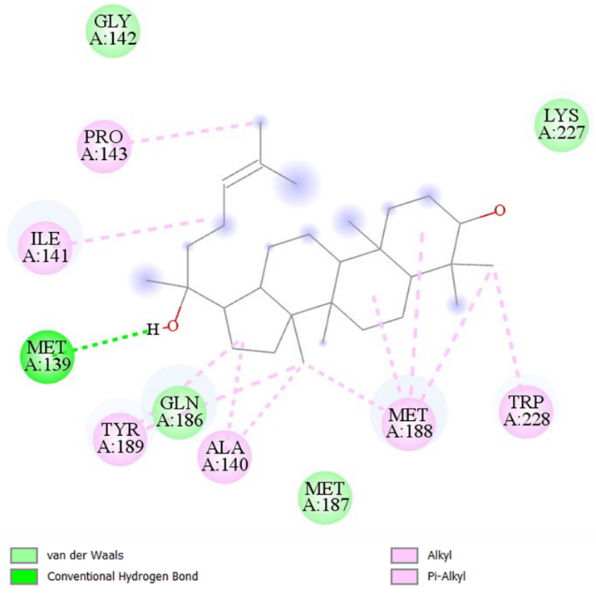

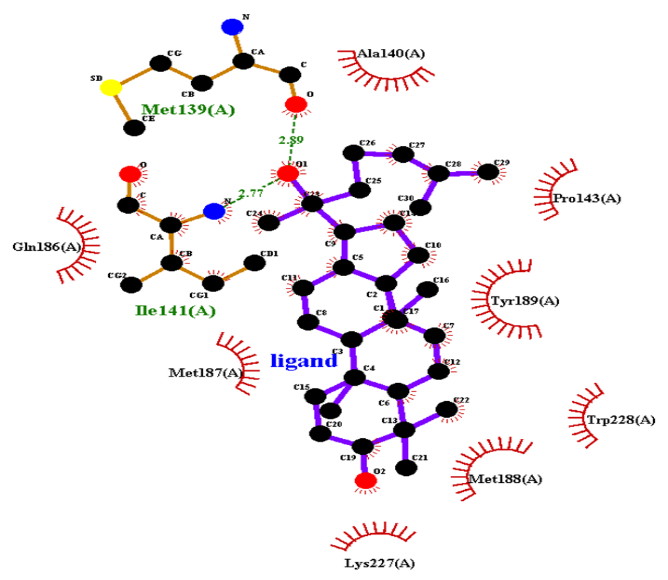
**

S**12: Molecular Docking, secondary structure interaction and ligplus analysis of Ginsenosides interactions with Sortilin.**

**
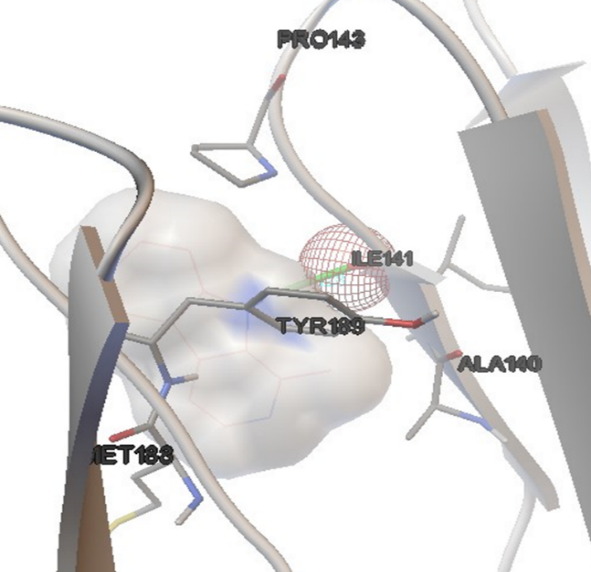

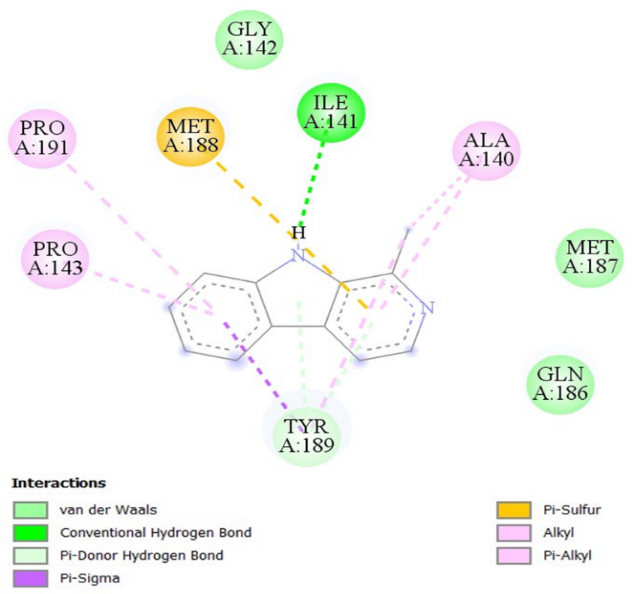

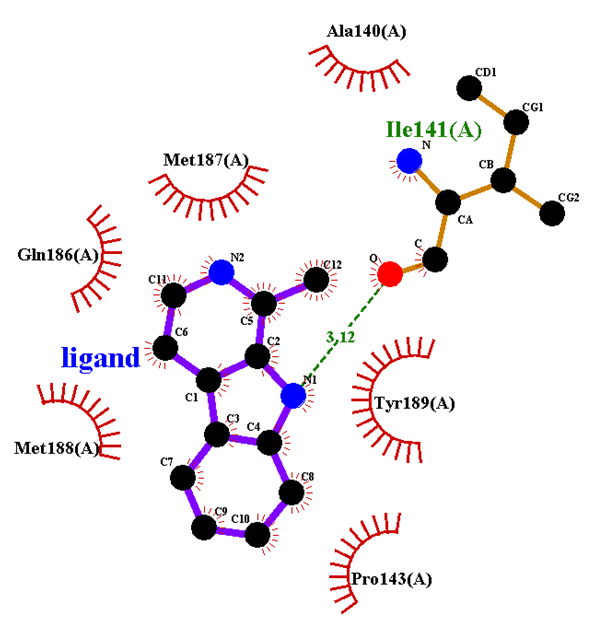
**

**S13: Molecular Docking, secondary structure interaction and ligplus analysis of Harman interactions with Sortilin.**


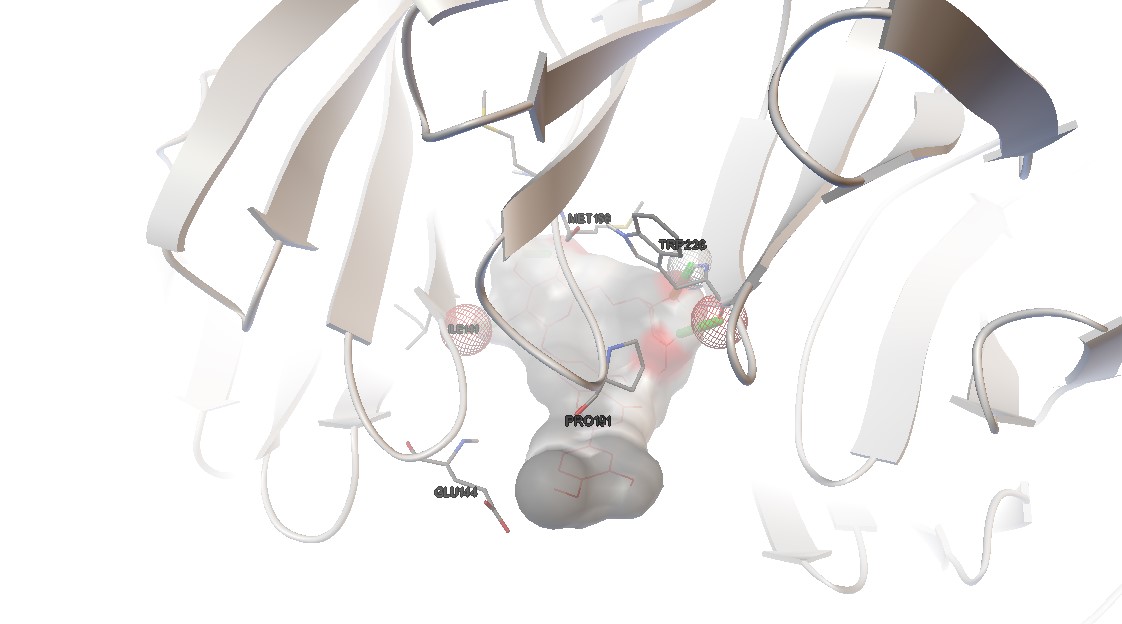
 **
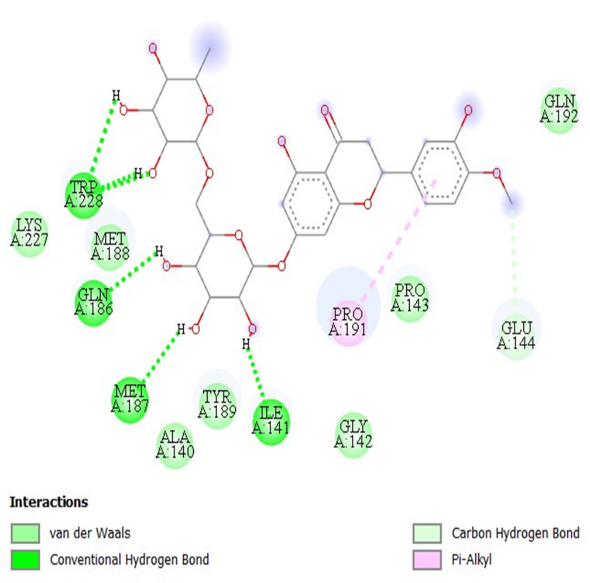

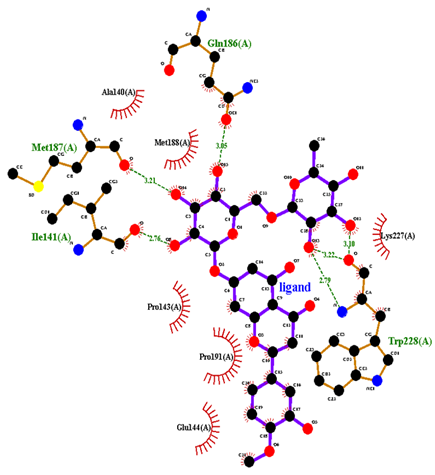
**

**S14: Molecular Docking, secondary structure interaction and ligplus analysis of Hesperidin interactions with Sortilin.**


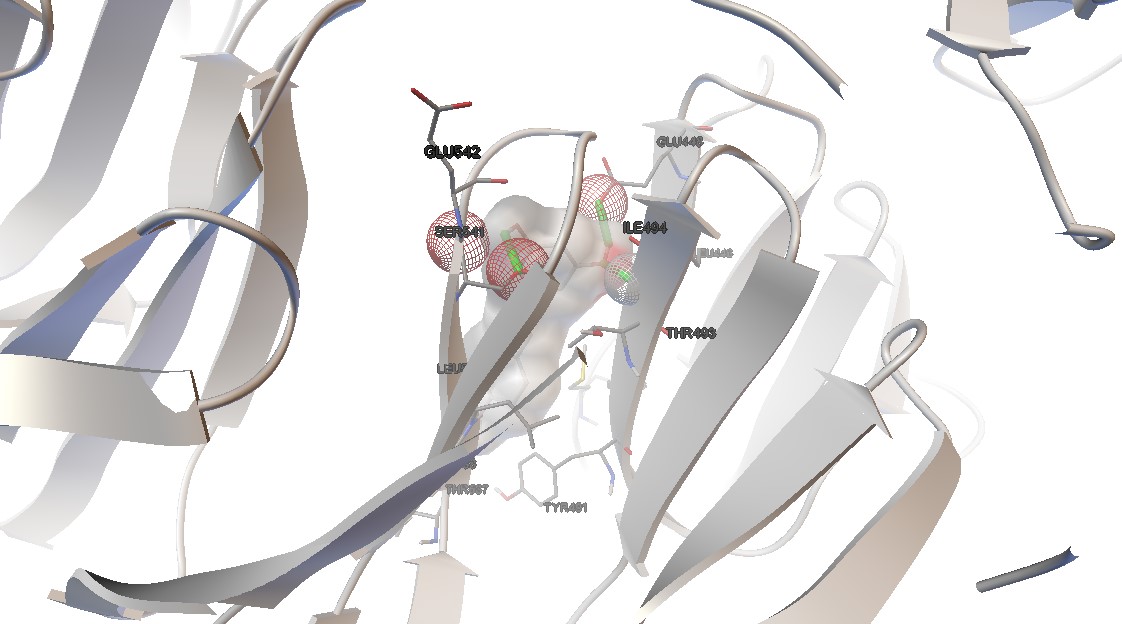

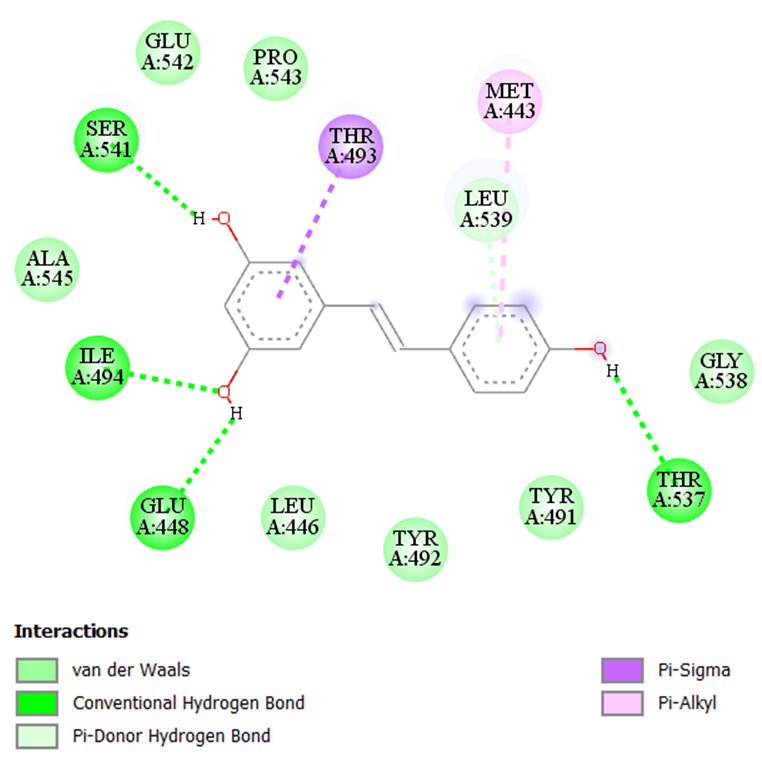

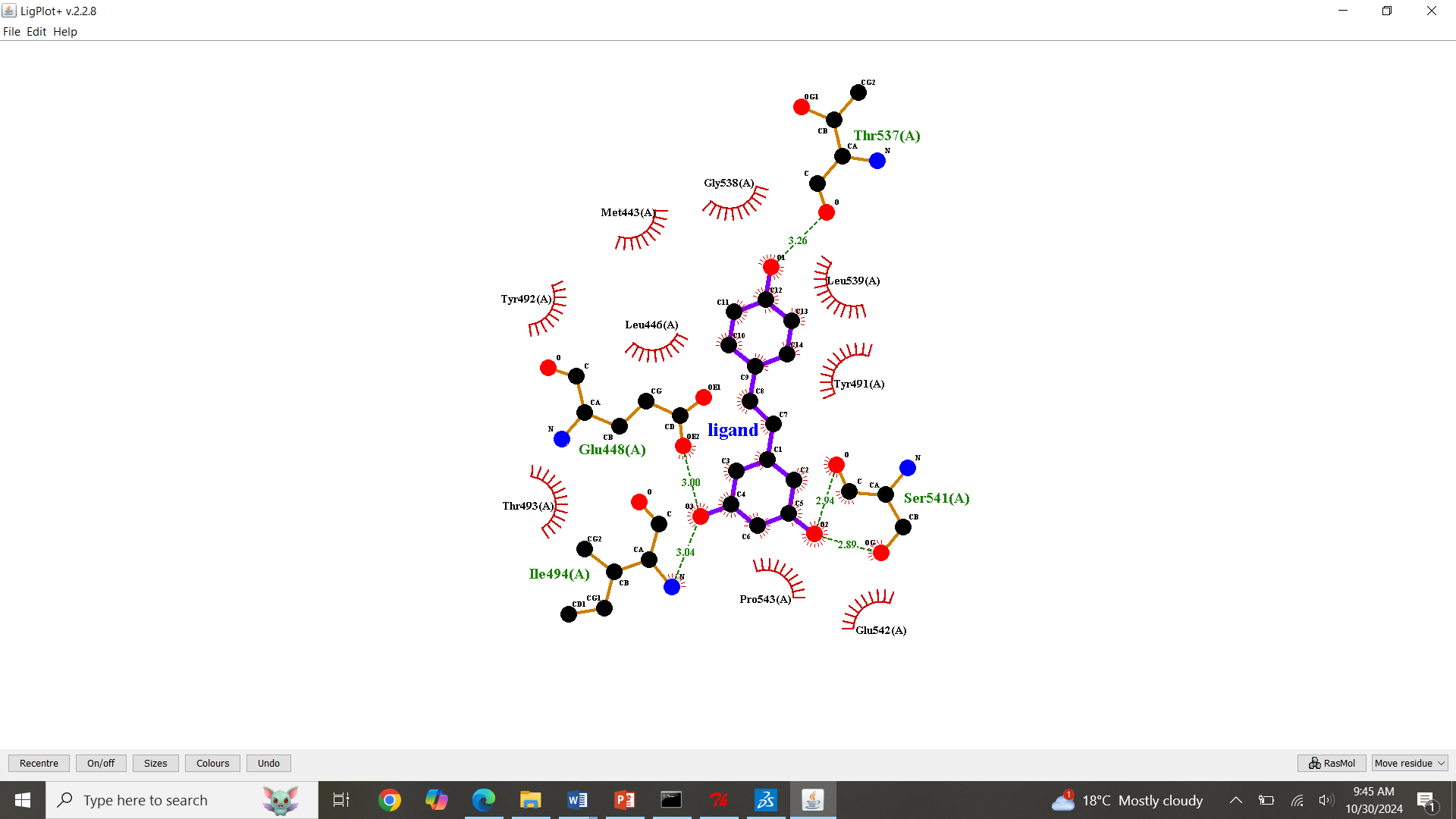


**S15: Molecular Docking, secondary structure interaction and ligplus analysis of Resveratrol interactions with Sortilin.**


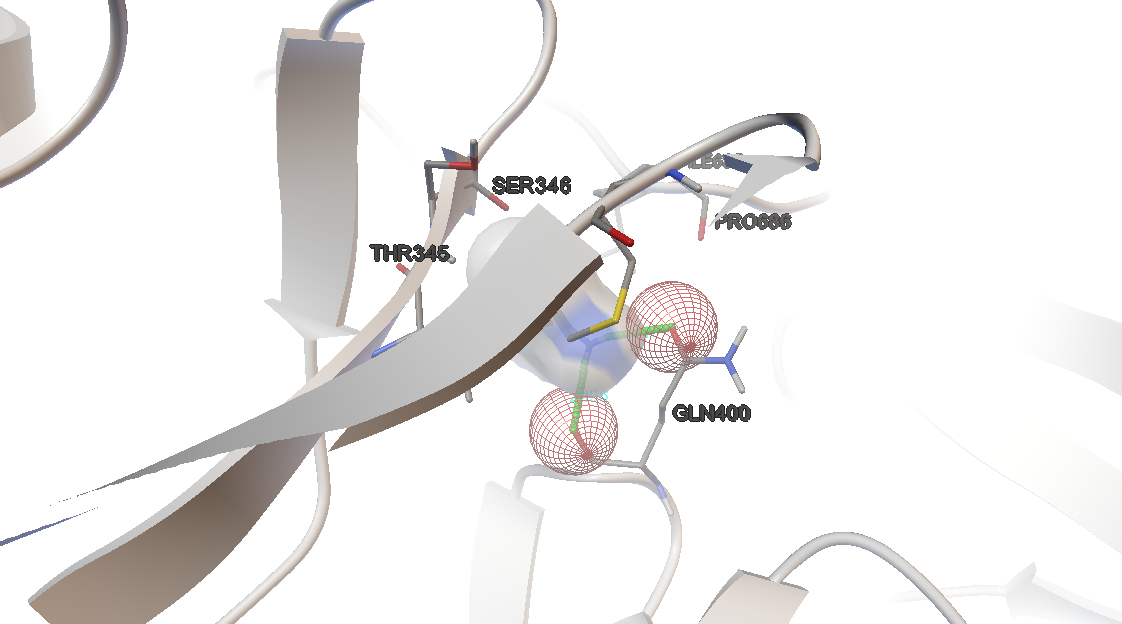

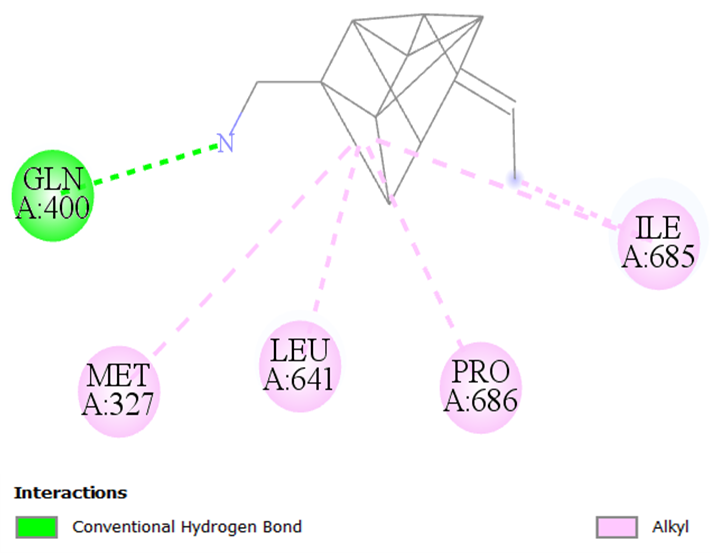

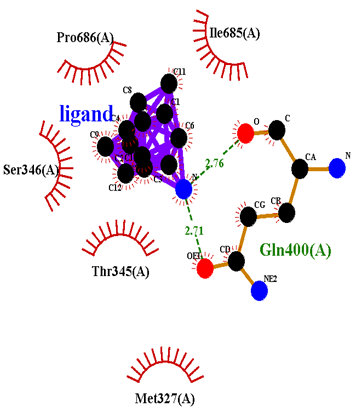


**S16: Molecular Docking, secondary structure interaction and ligplus analysis of Memantine interactions with Sortilin.**


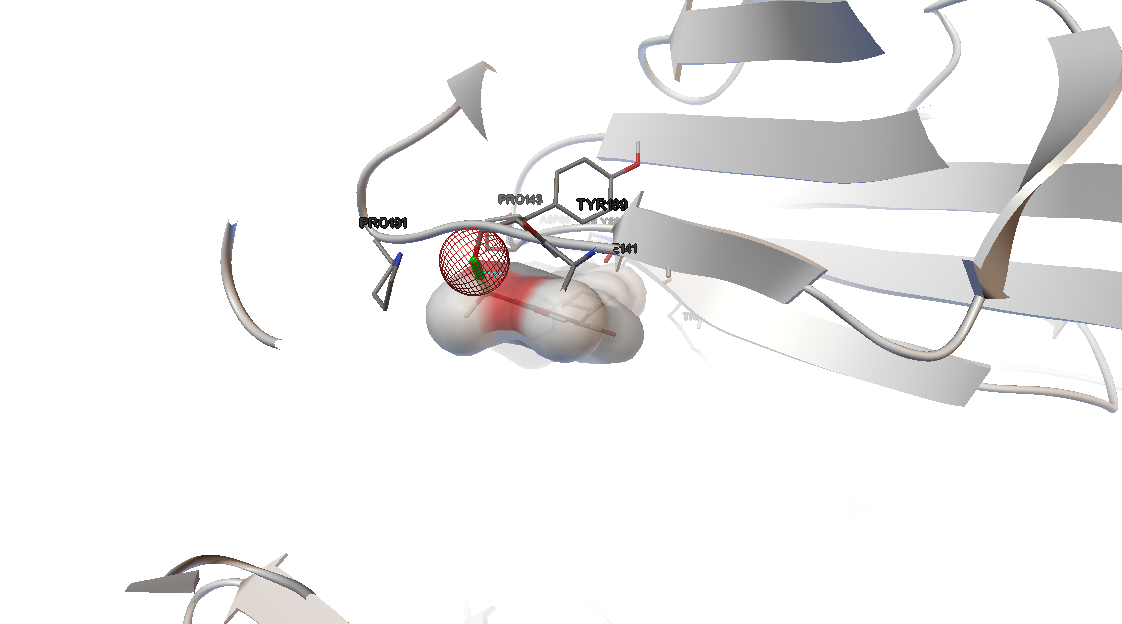

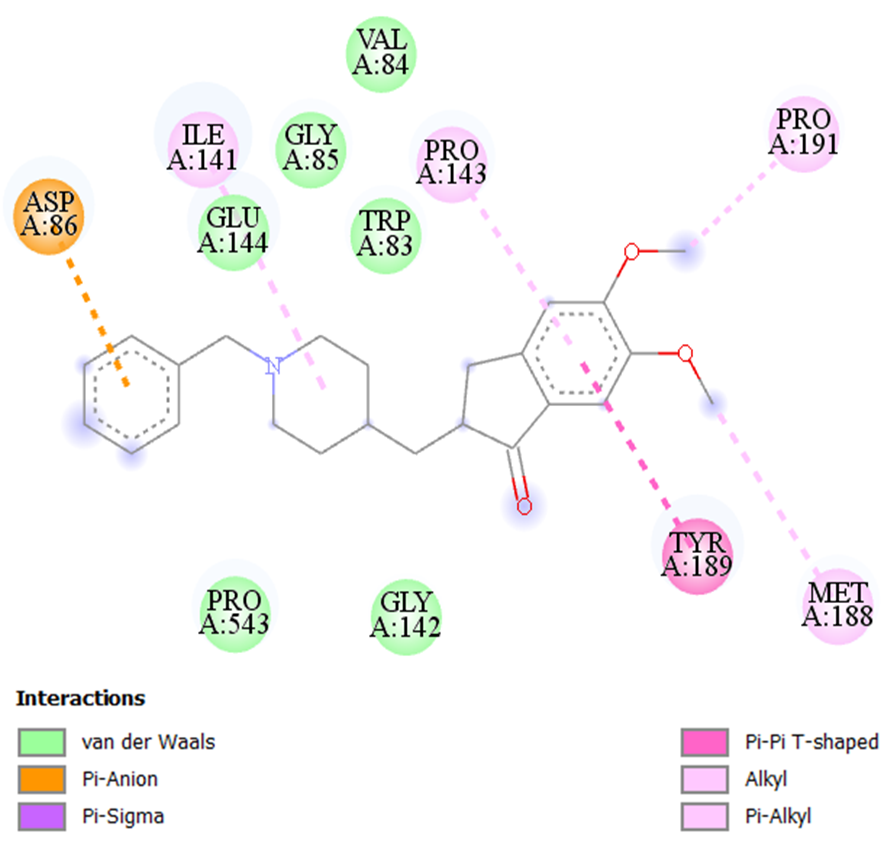

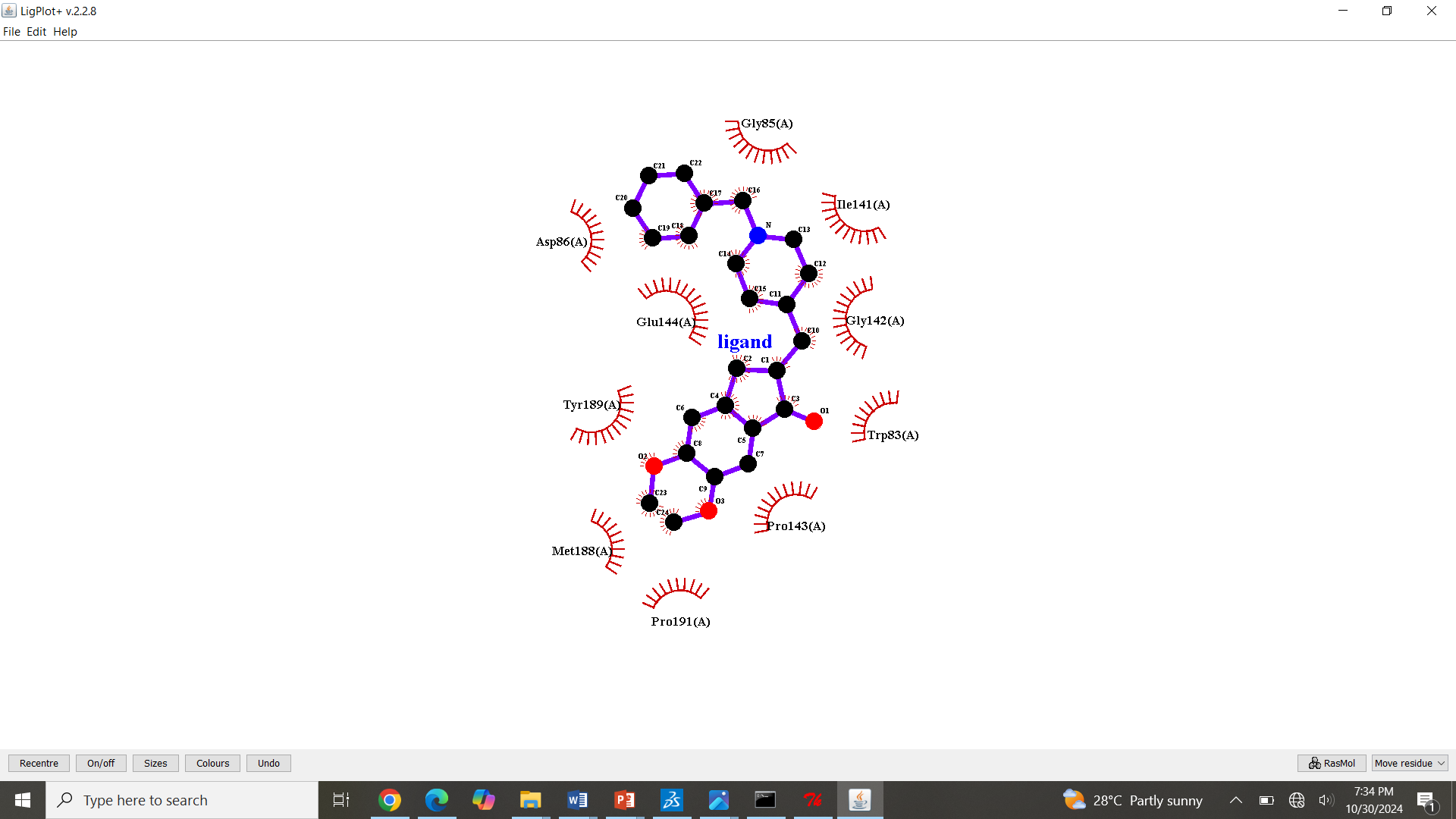


S**17: Molecular Docking, secondary structure interaction and ligplus analysis of Donepezil interactions with Sortilin.**


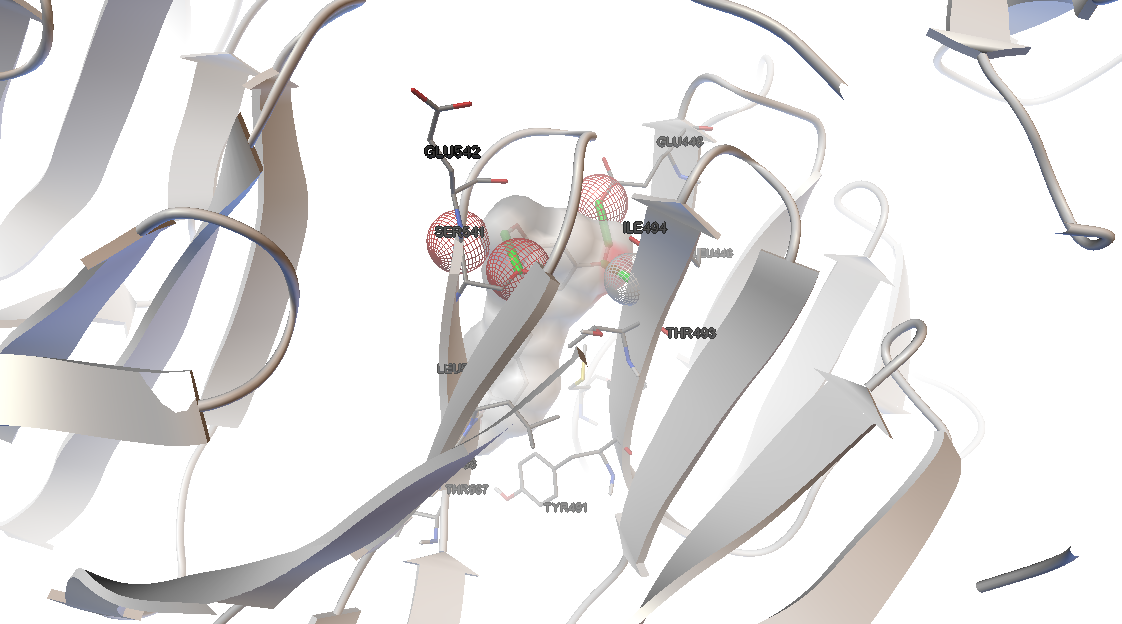

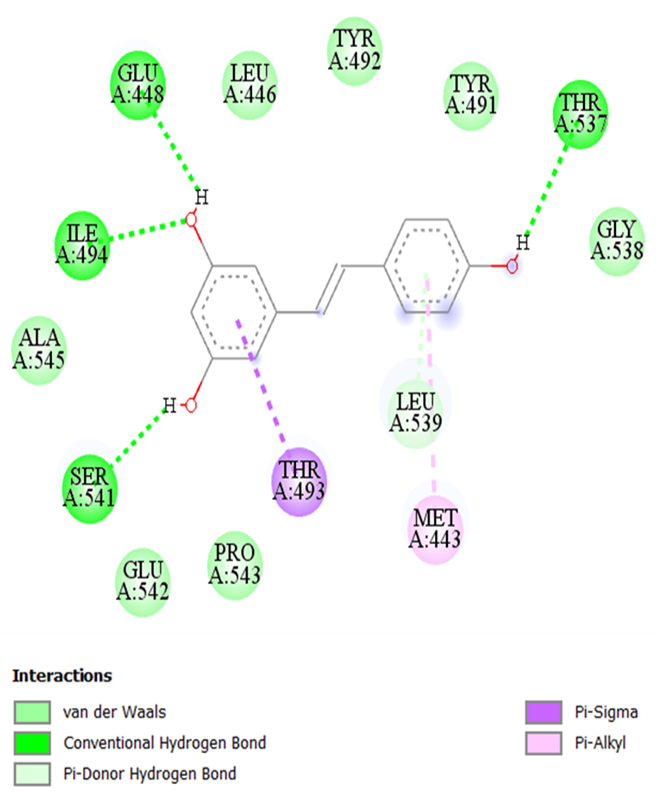

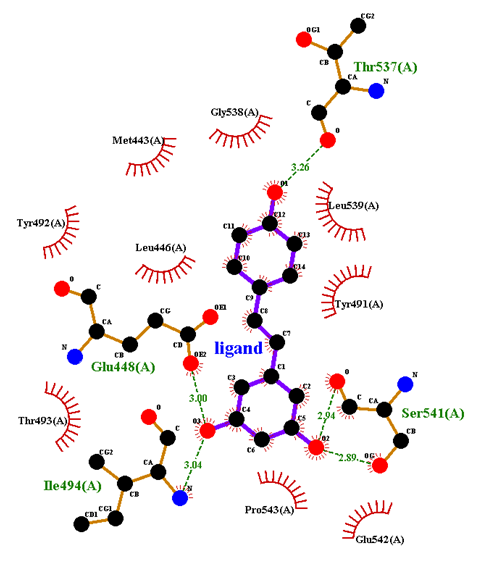


S**18: Molecular Docking, secondary structure interaction and ligplus analysis of Rivastigmine interactions with Sortilin.**


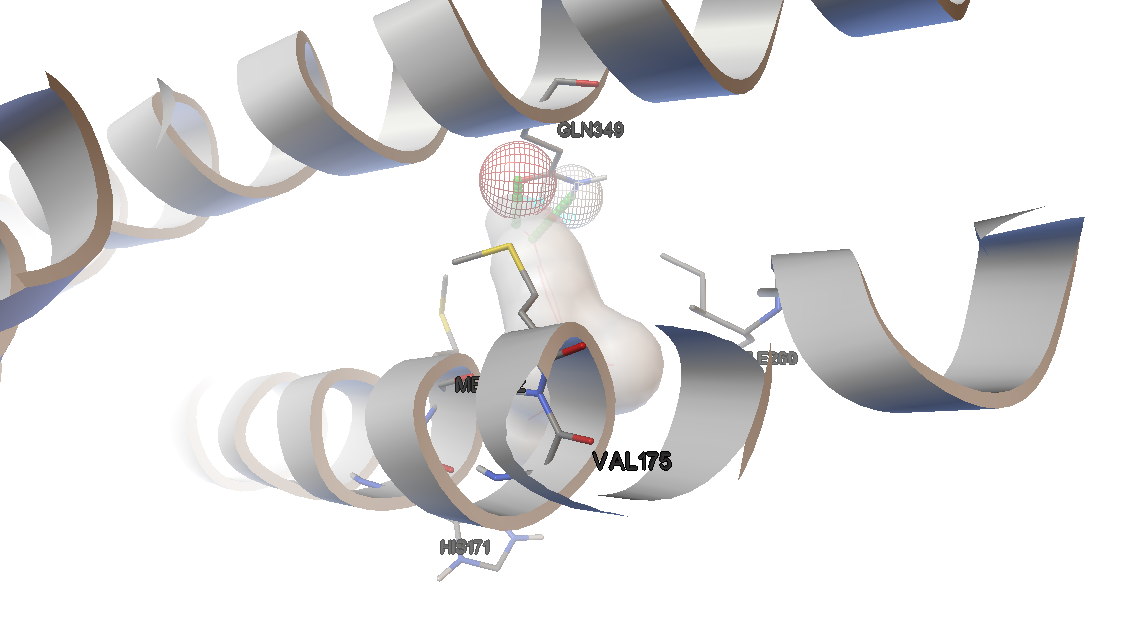

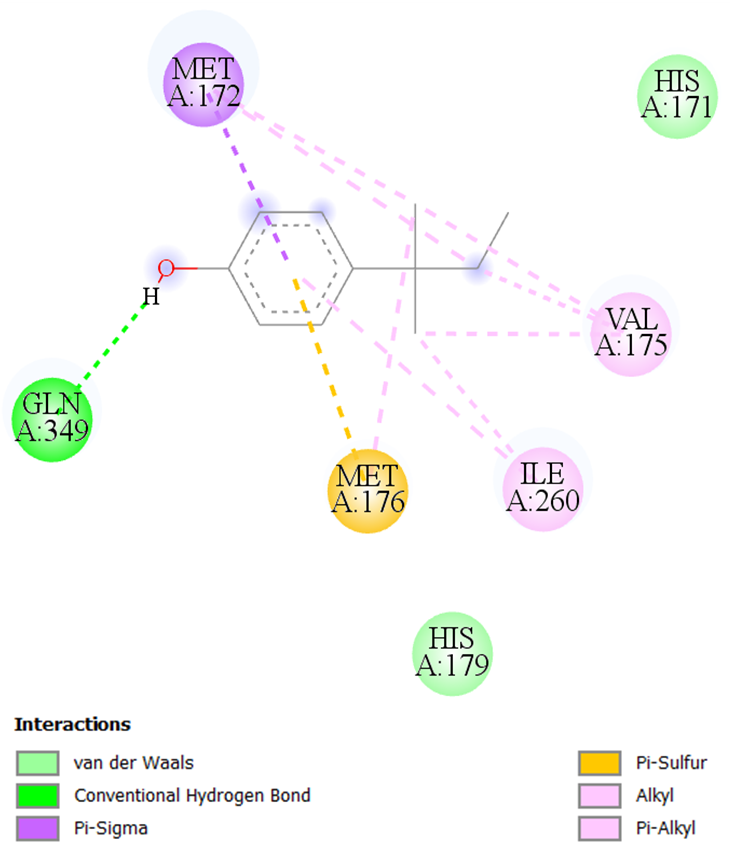

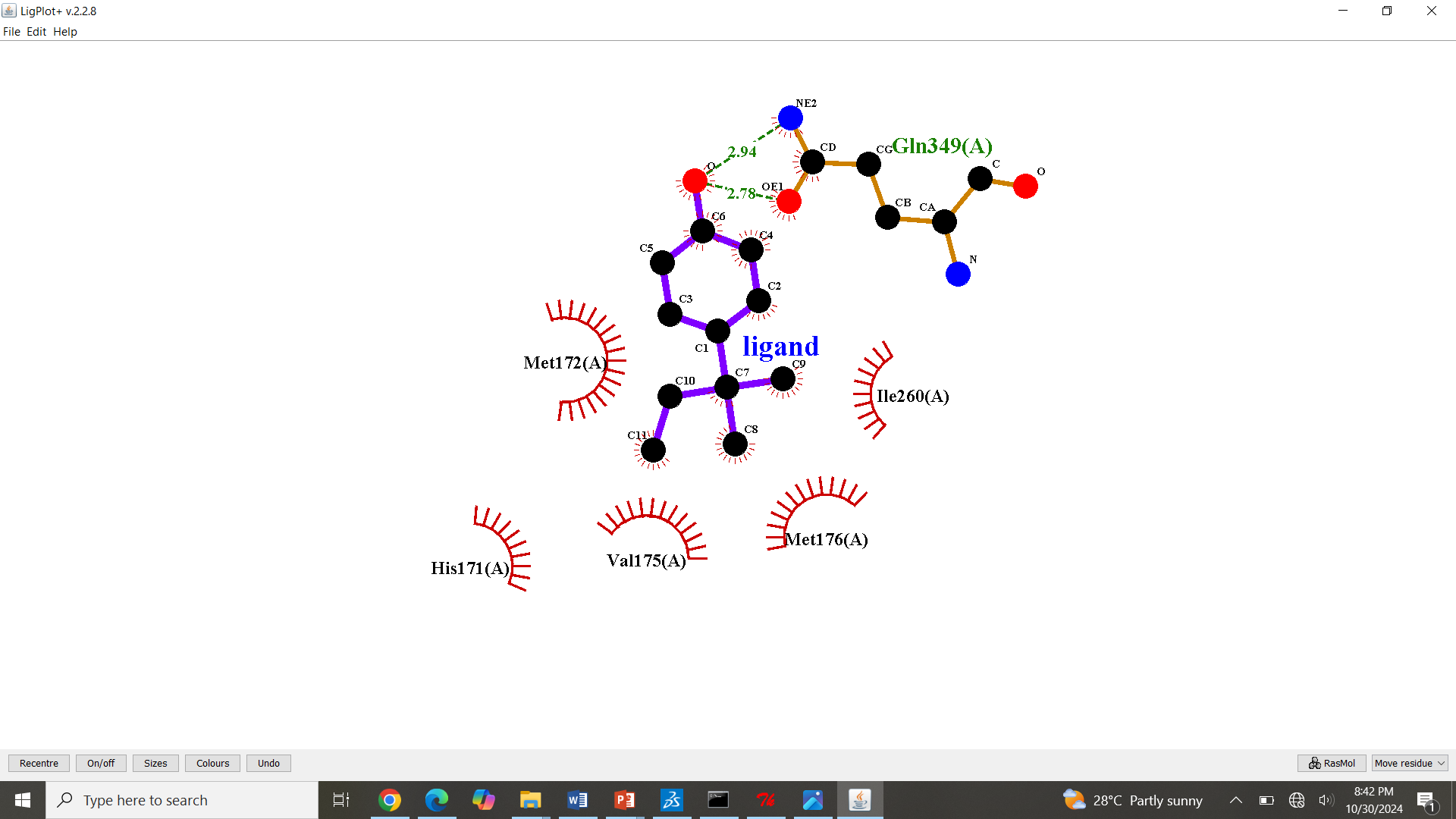


**S19: Molecular Docking, secondary structure interaction and ligplus analysis of 4-tert-Amylphenol interactions with Clustrin**.


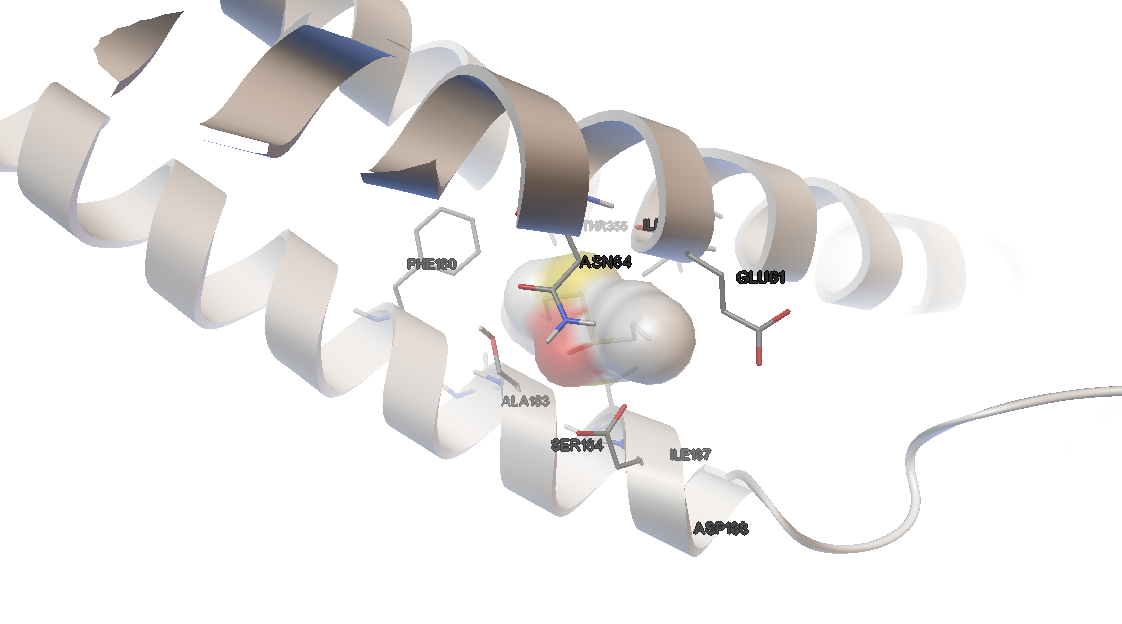

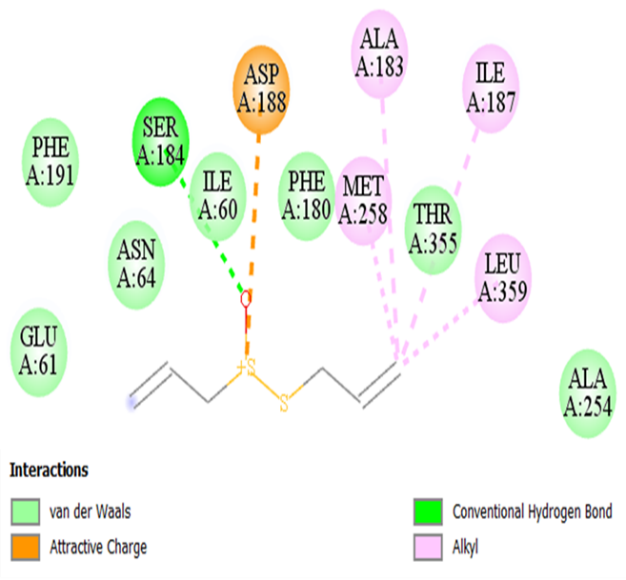

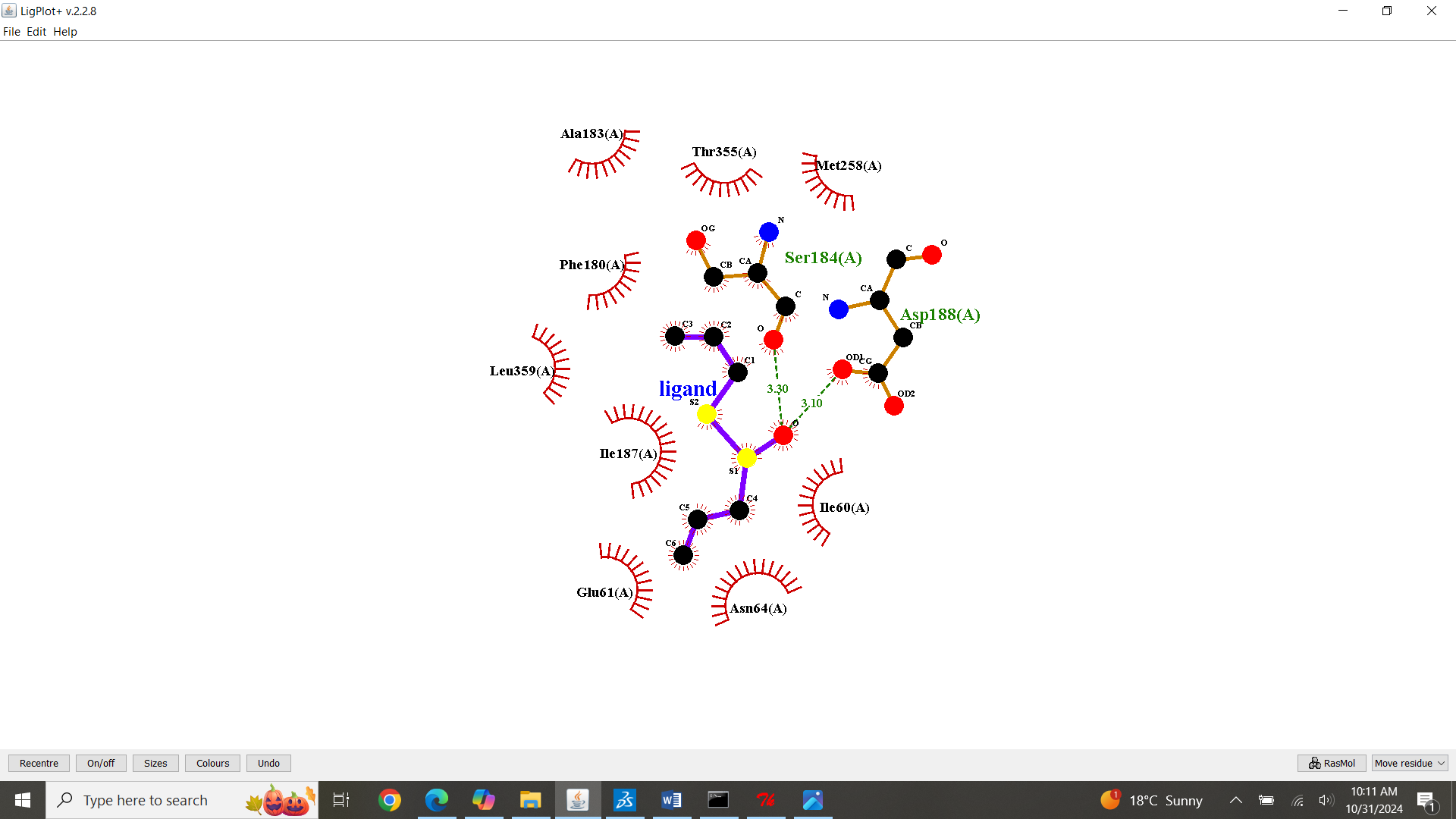


**S20: Molecular Docking, secondary structure interaction and ligplus analysis of Allicin interactions with Clustrin**.


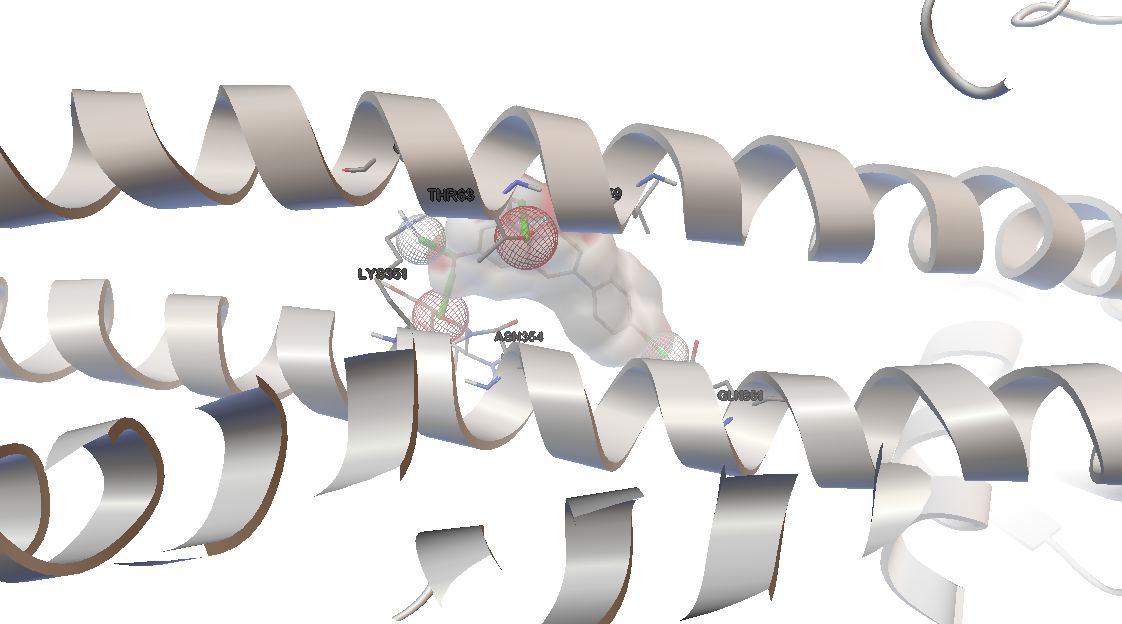

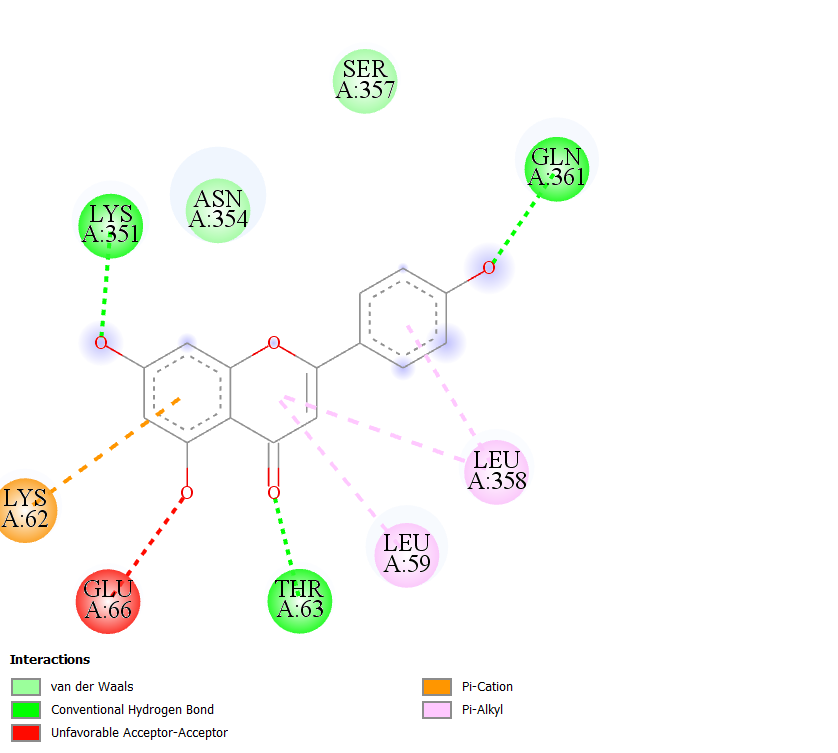

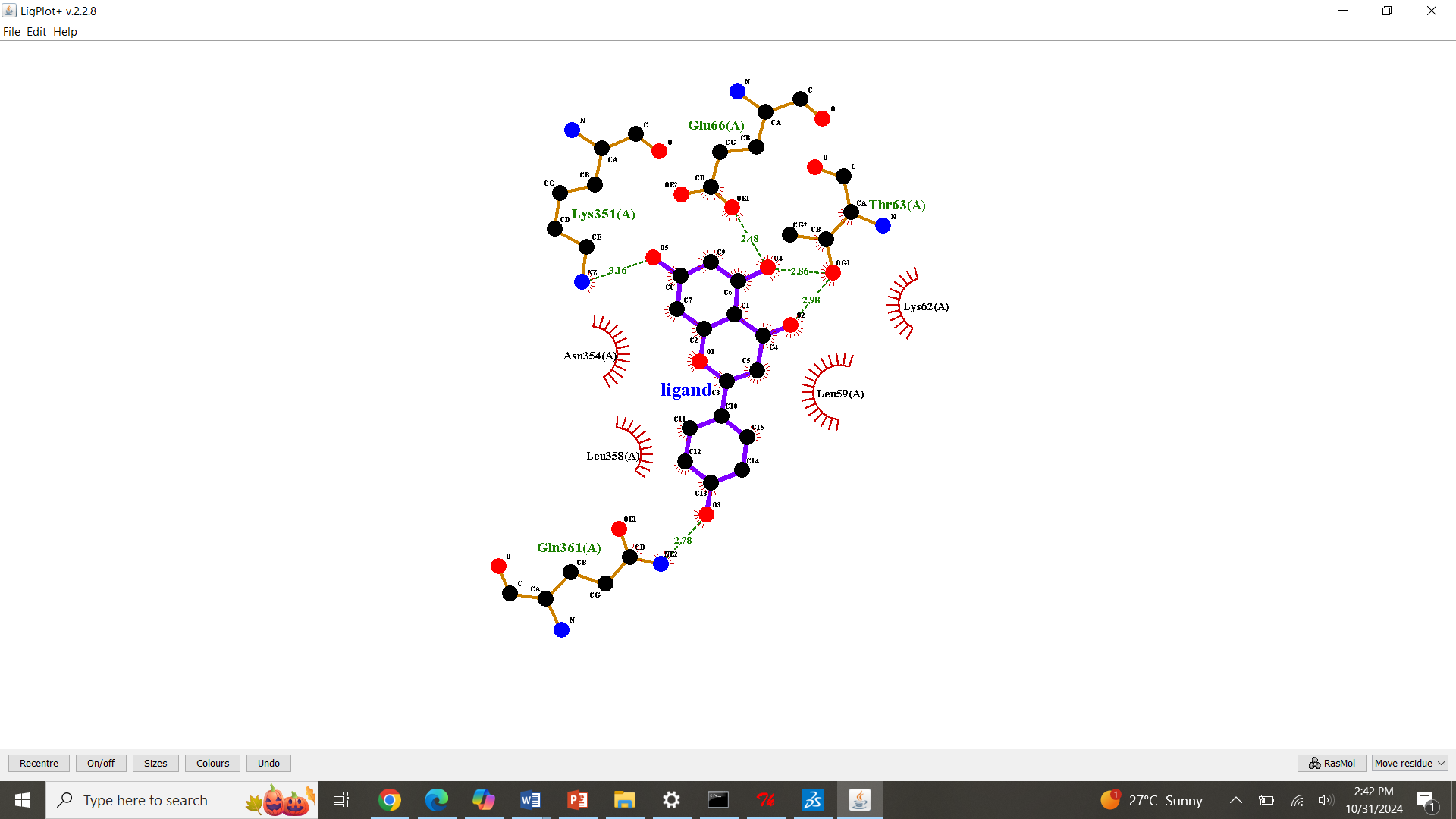


**S21: Molecular Docking, secondary structure interaction and ligplus analysis of Apigenin interactions with Clustrin**.


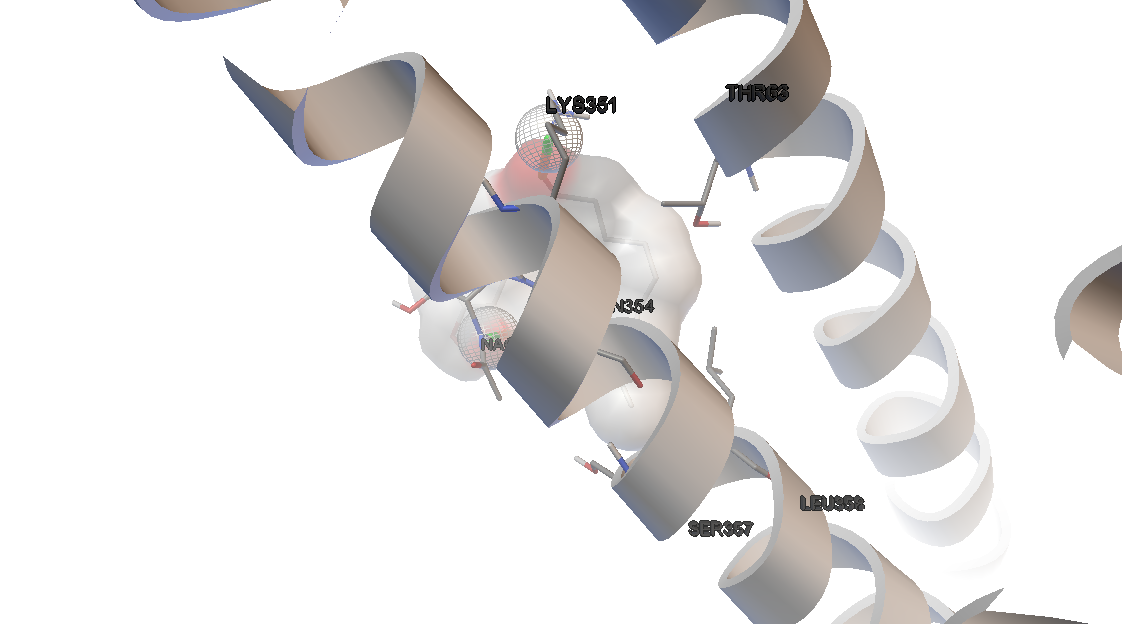

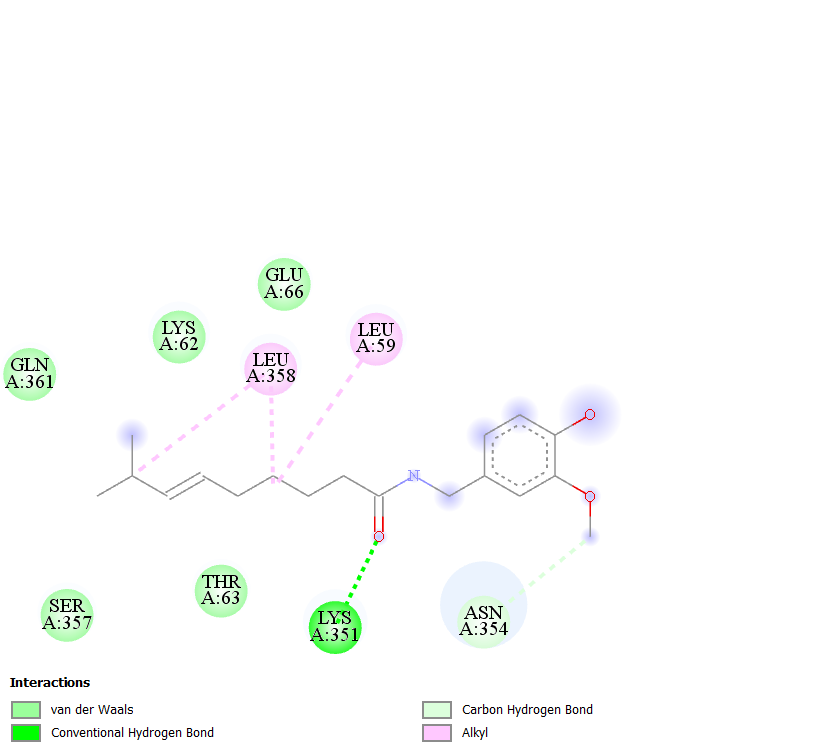

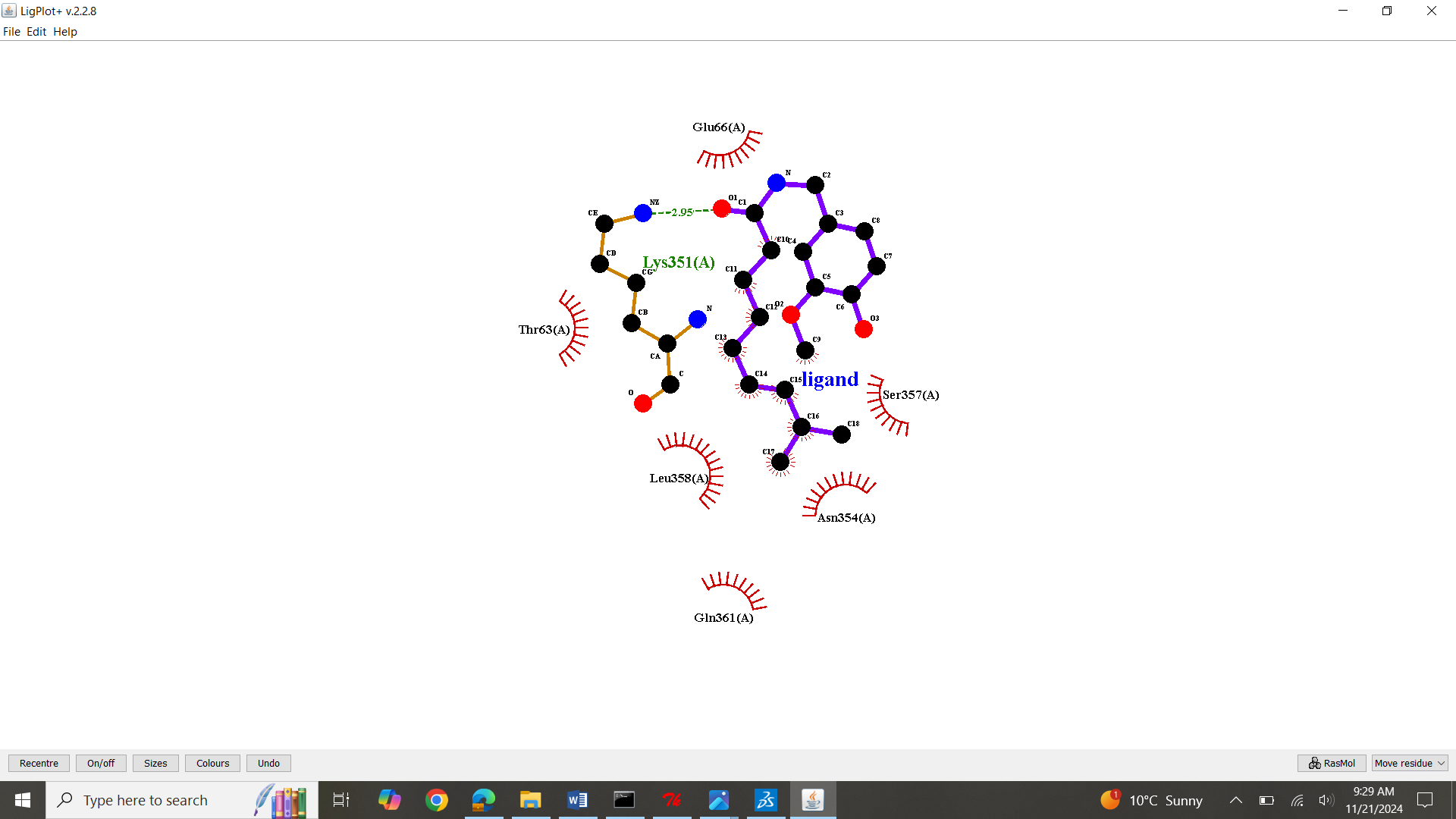


**S22: Molecular Docking, secondary structure interaction and ligplus analysis of Astragalosides interactions with Clustrin**.


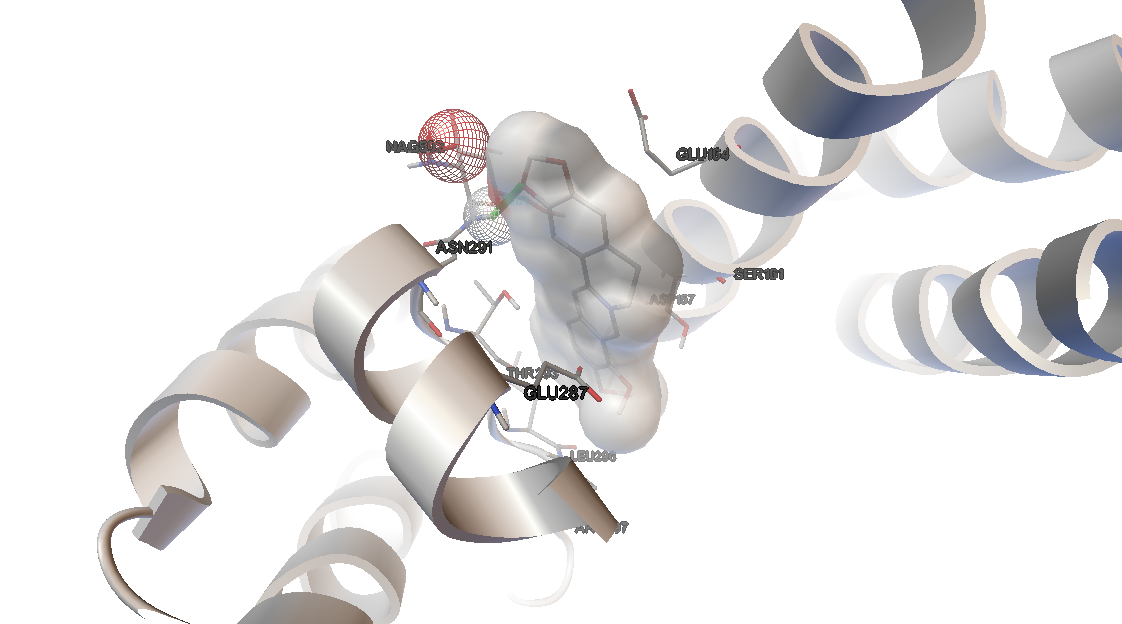

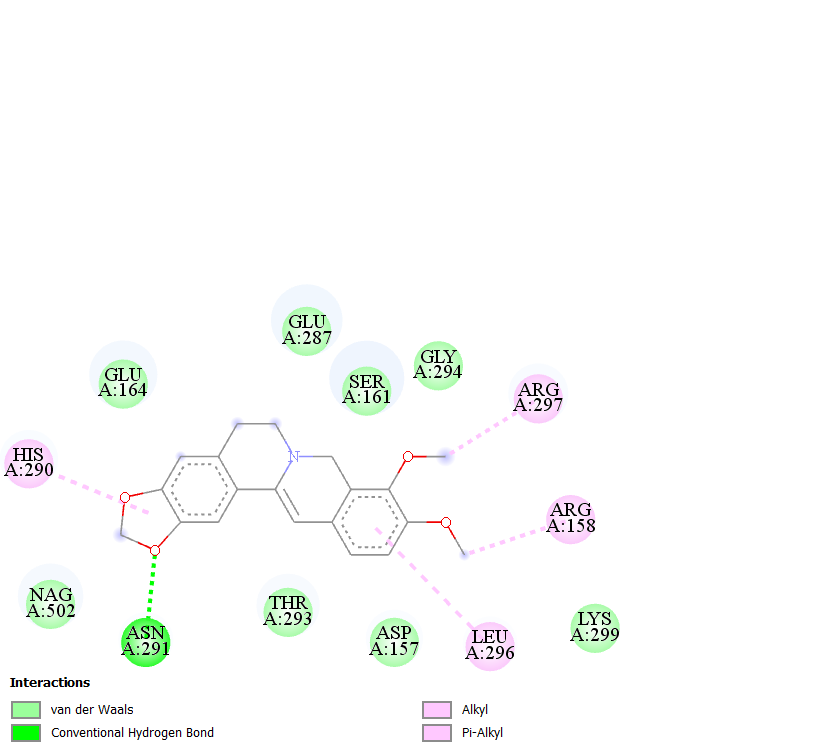

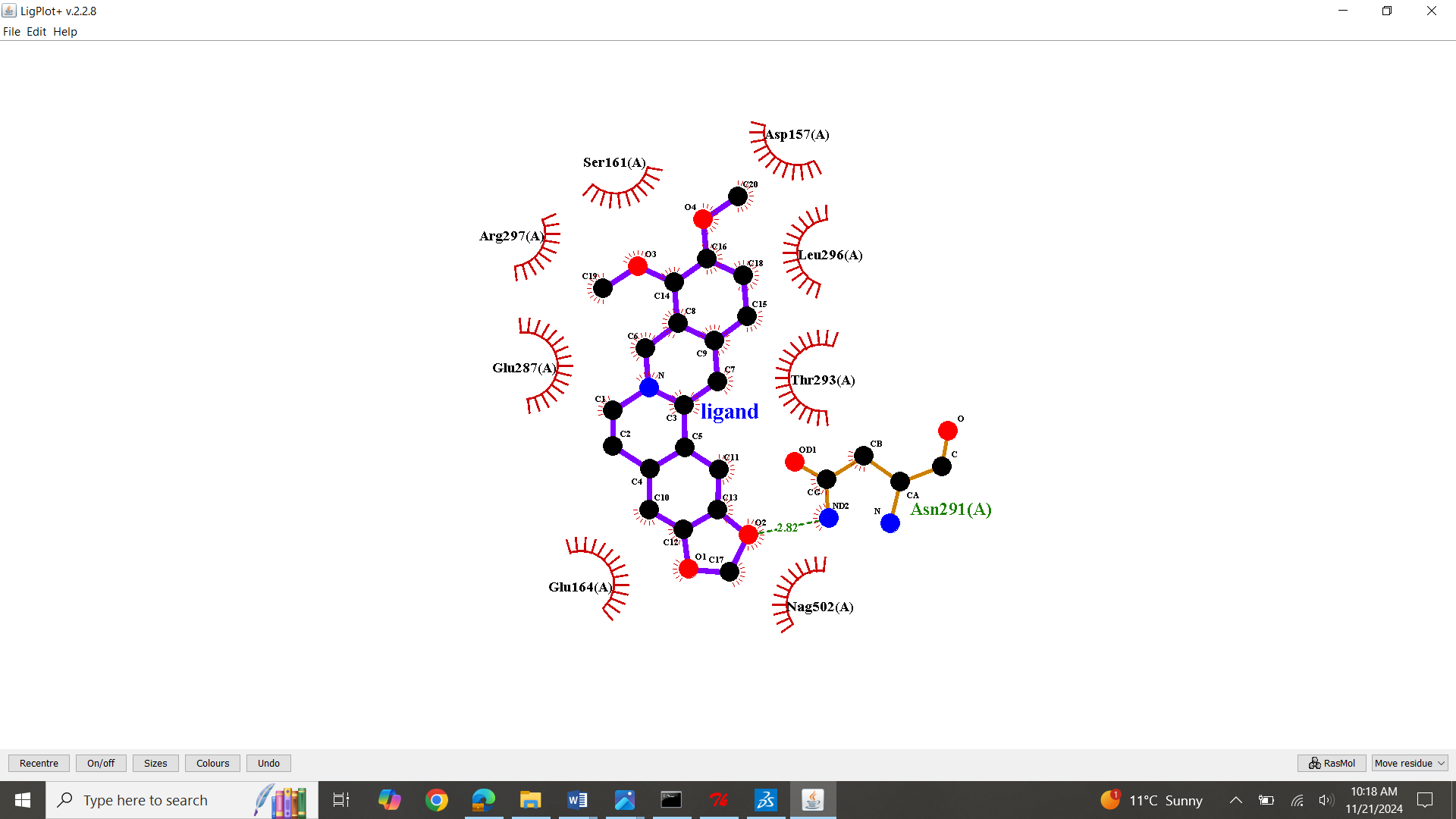


**S23: Molecular Docking, secondary structure interaction and ligplus analysis of Berberine interactions with Clustrin**.


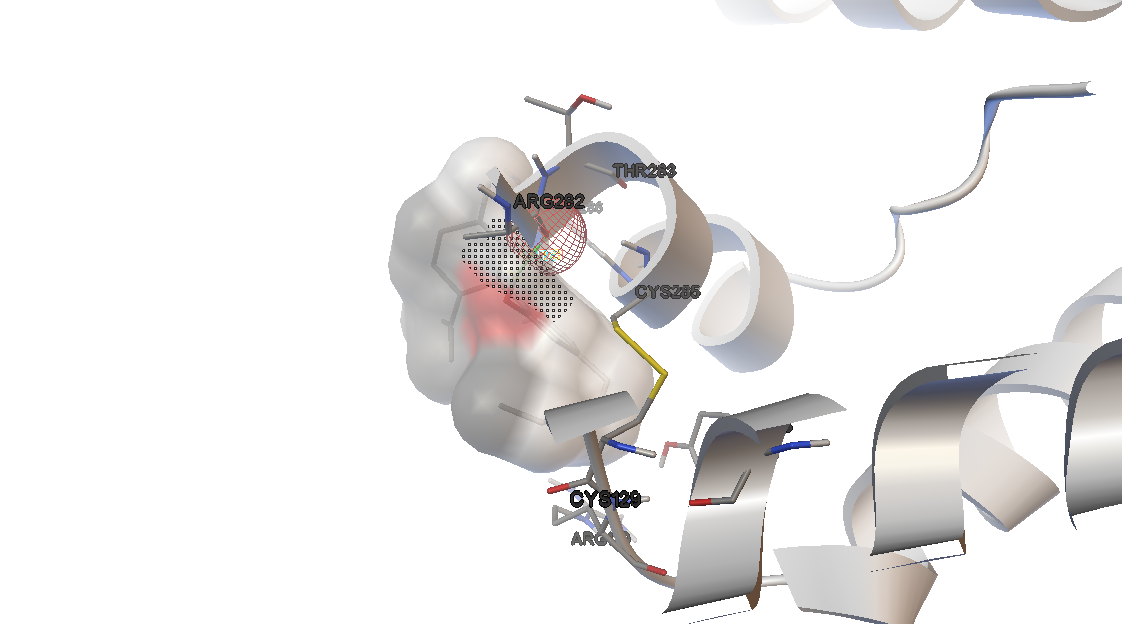

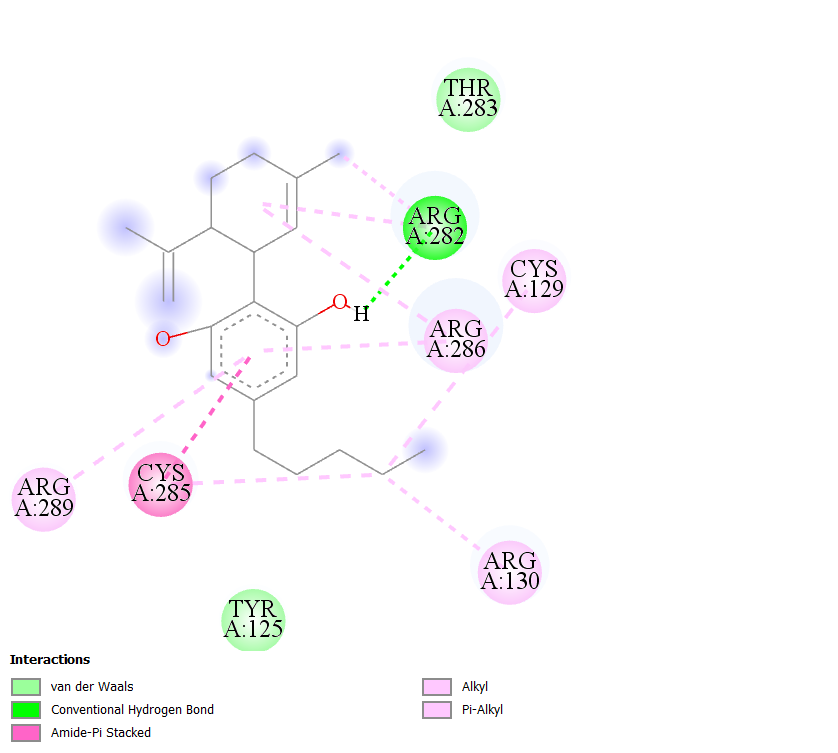

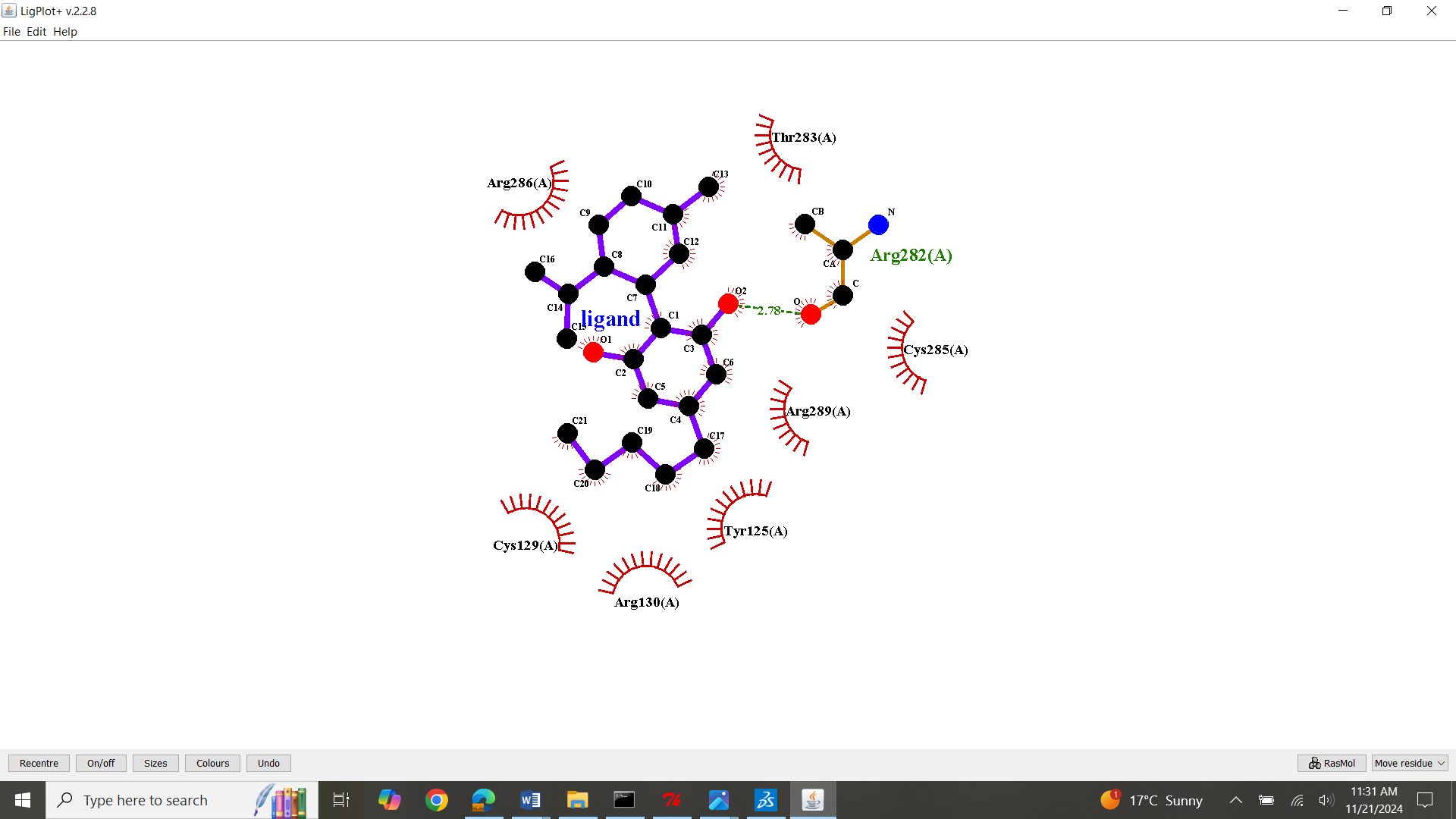


**S24: Molecular Docking, secondary structure interaction and ligplus analysis of Cannabidiol interactions with Clustrin**.


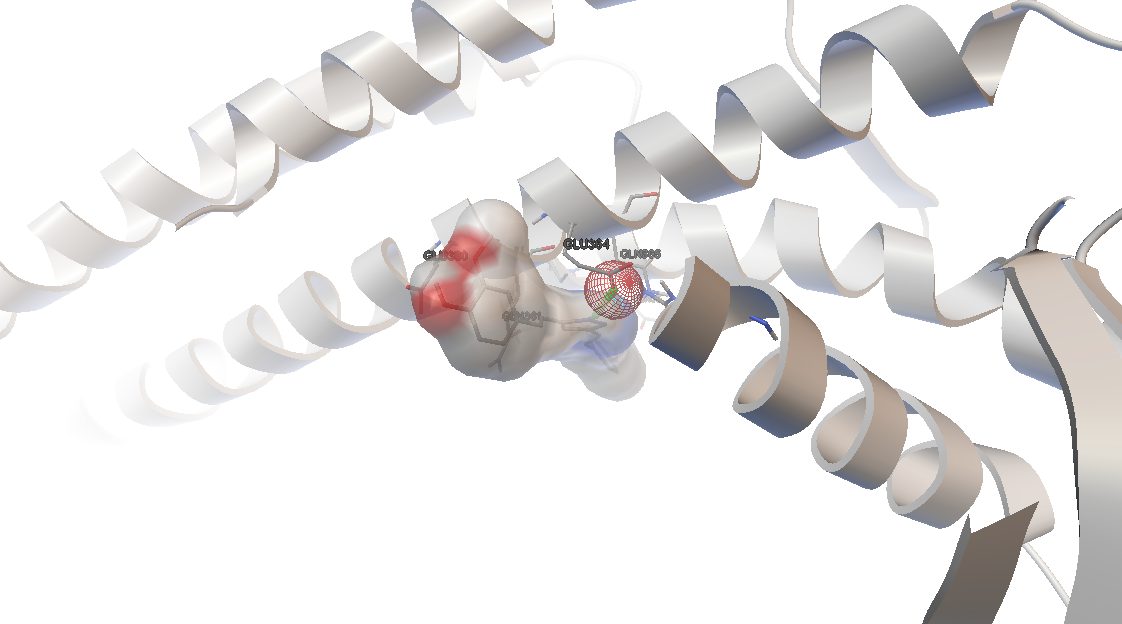

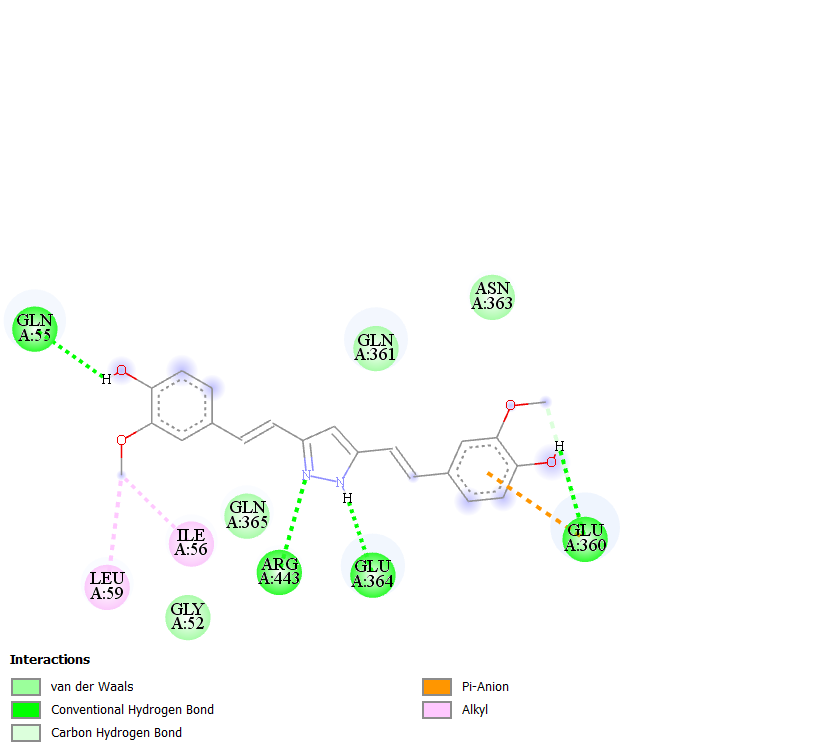

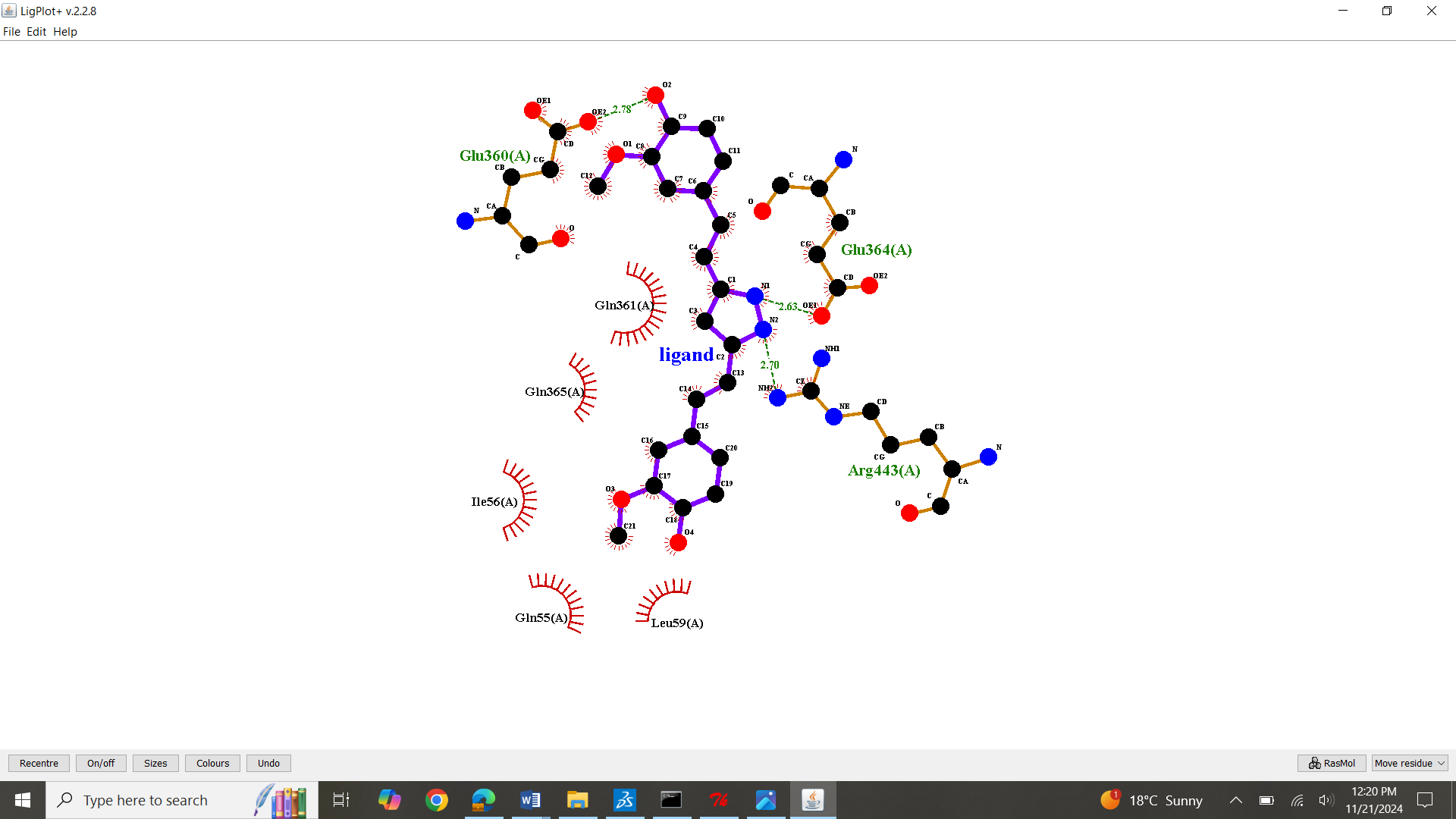


**S25: Molecular Docking, secondary structure interaction and ligplus analysis of Curcumin interactions with Clustrin**.


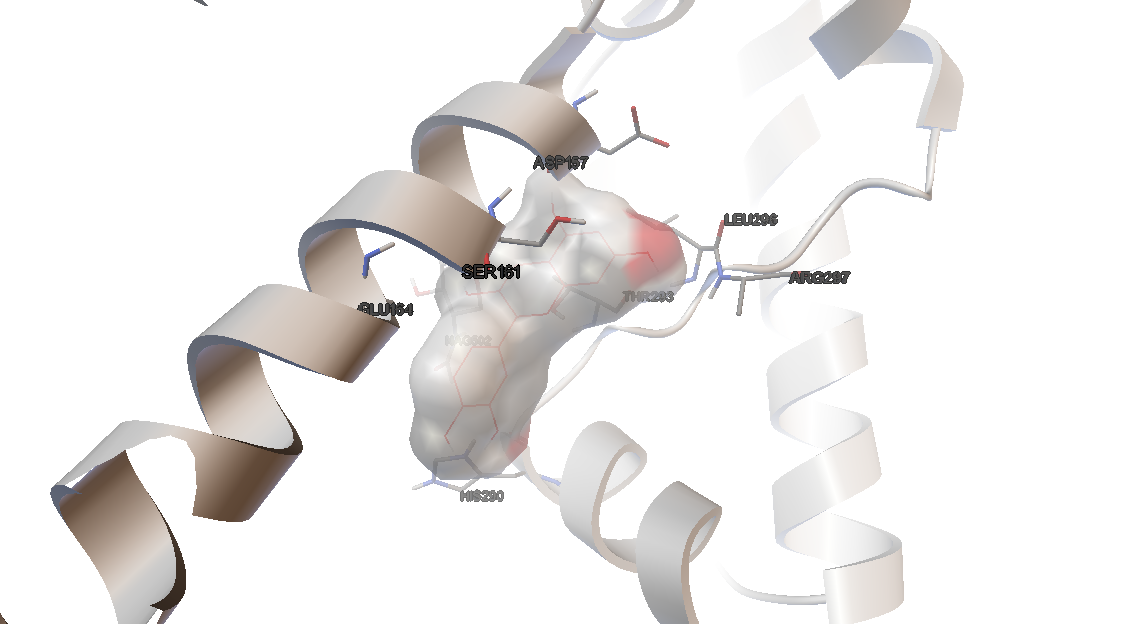

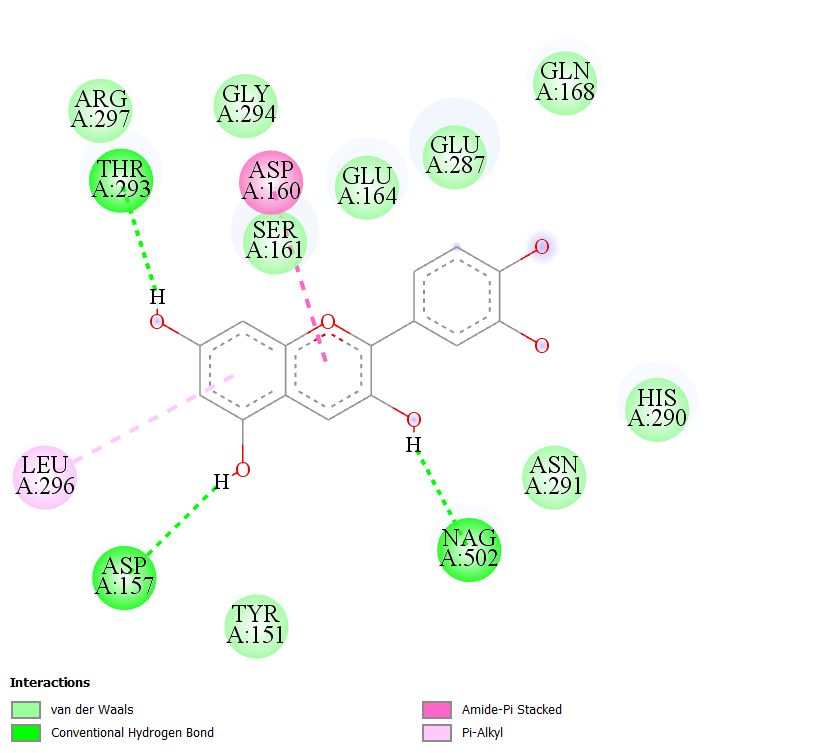

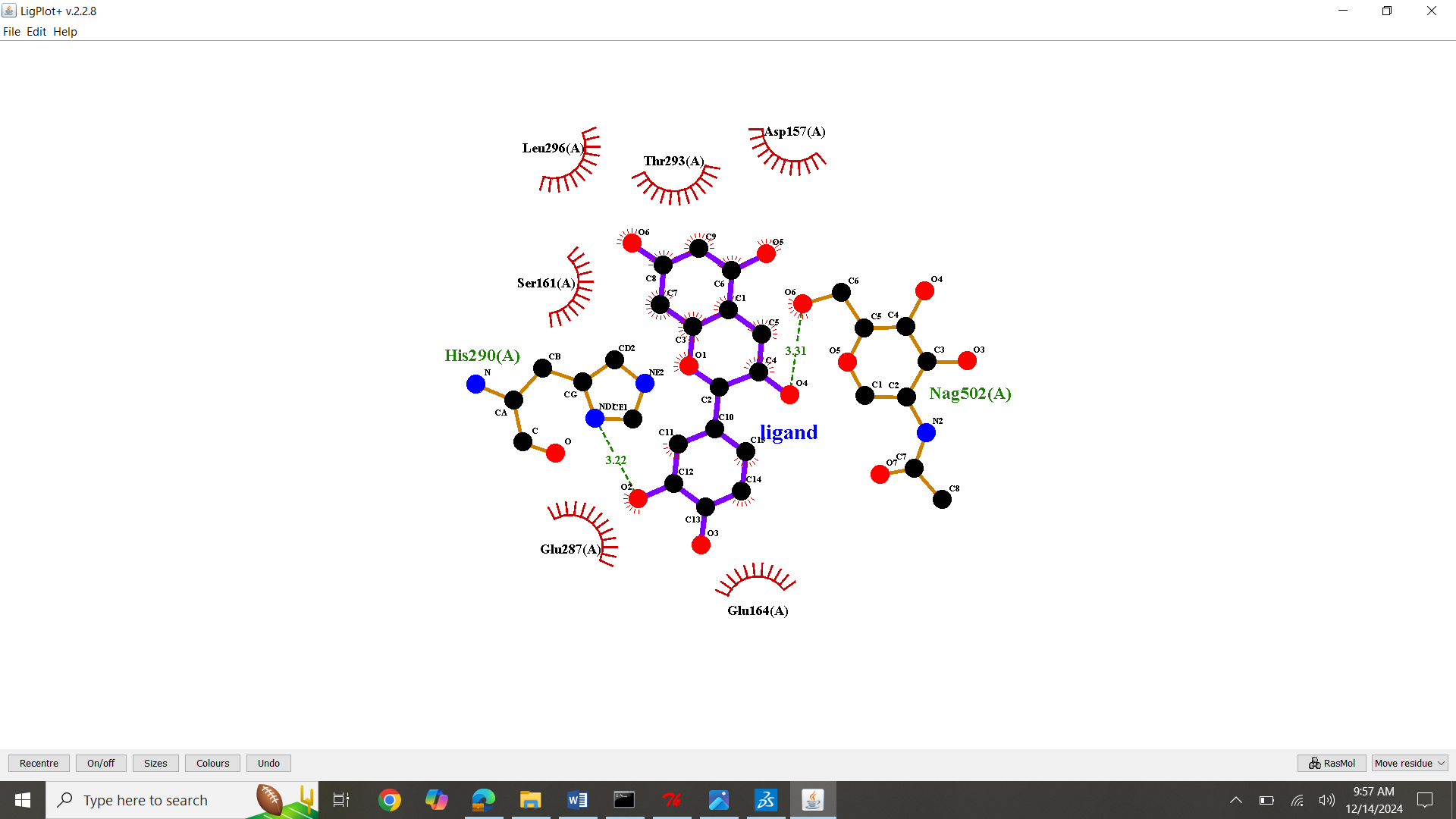


**S26: Molecular Docking, secondary structure interaction and ligplus analysis of Cynidin interactions with Clustrin**.


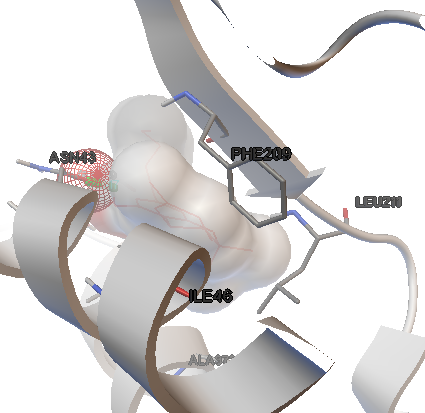

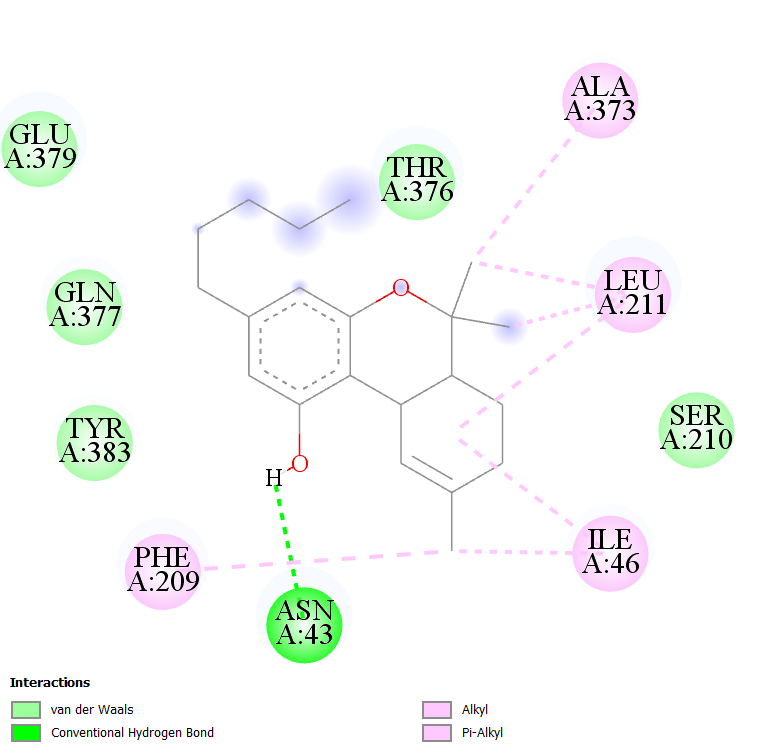

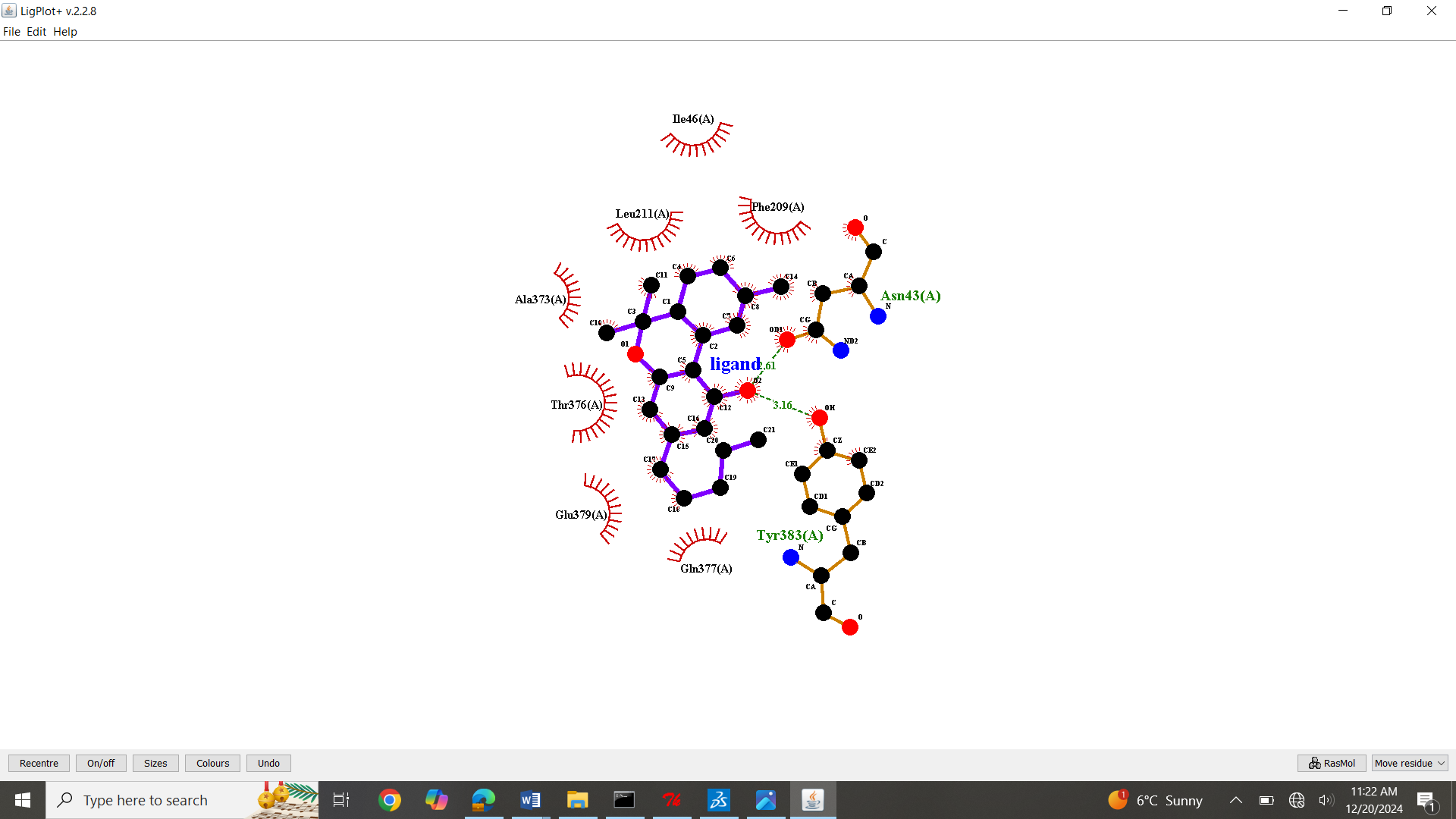


**S27: Molecular Docking, secondary structure interaction and ligplus analysis of Dronabinol interactions with Clustrin**.


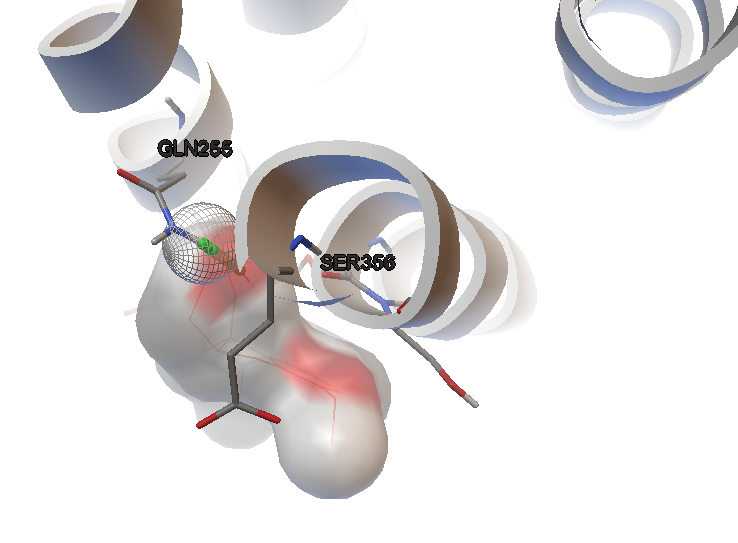

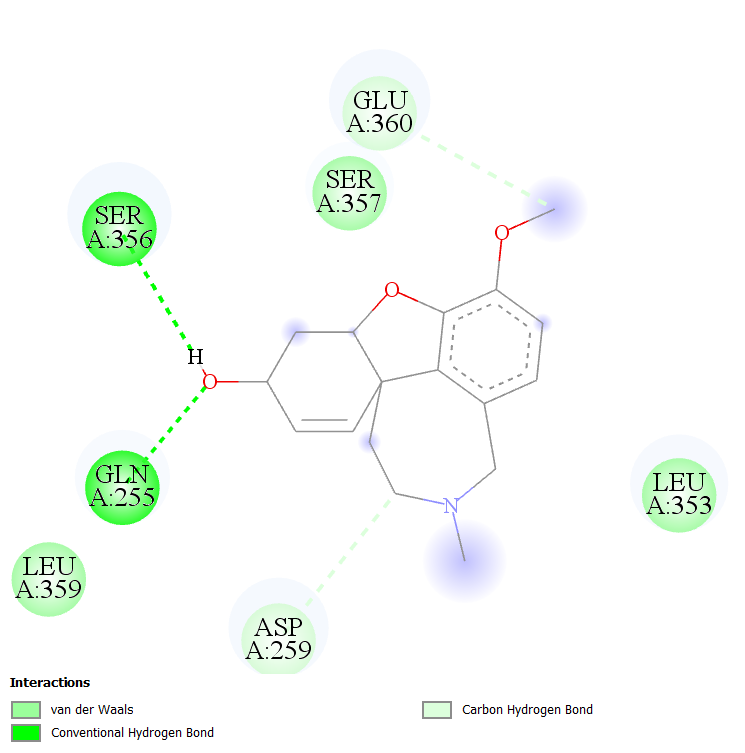

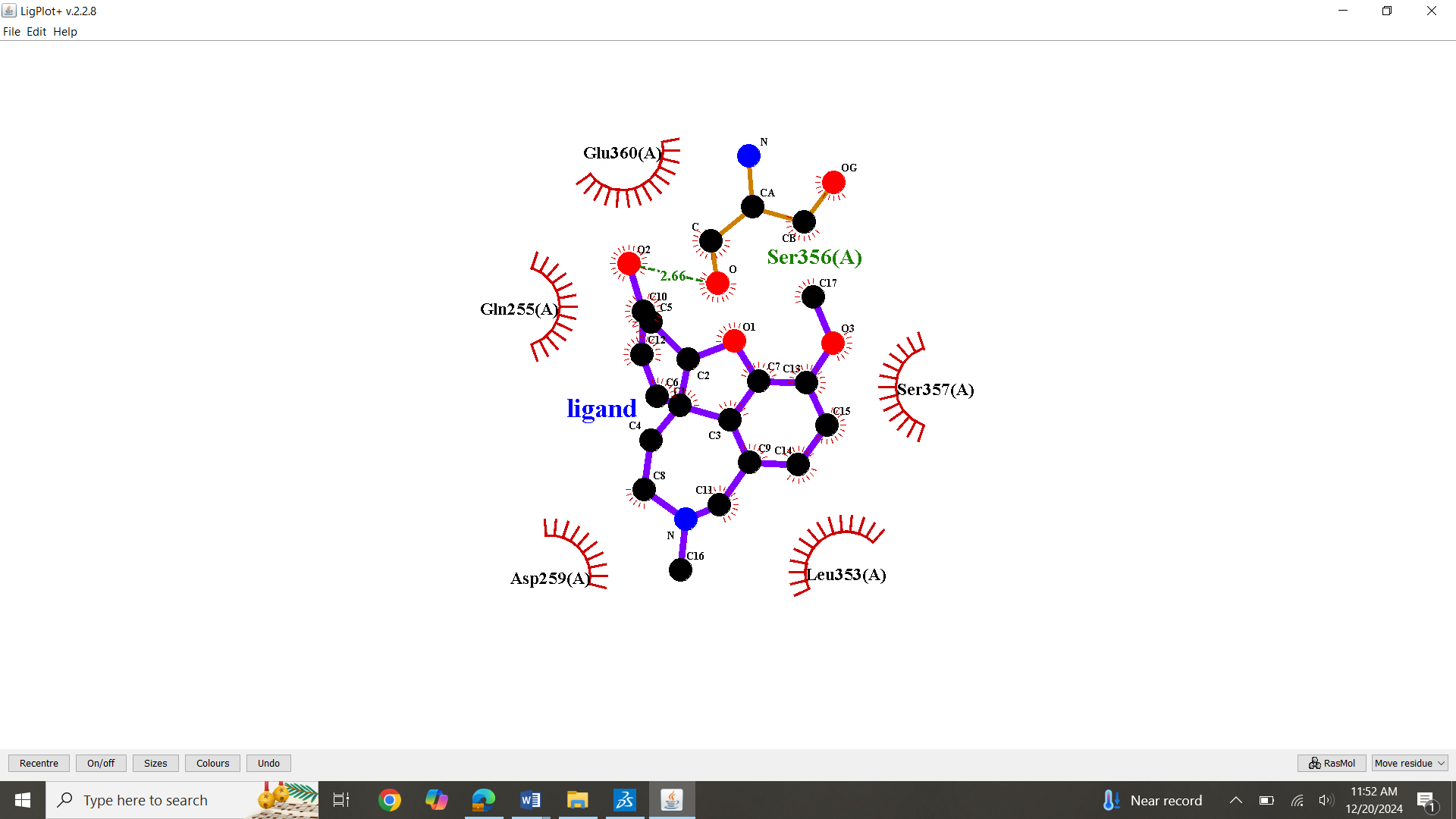


**S28: Molecular Docking, secondary structure interaction and ligplus analysis of Galantamine interactions with Clustrin**.


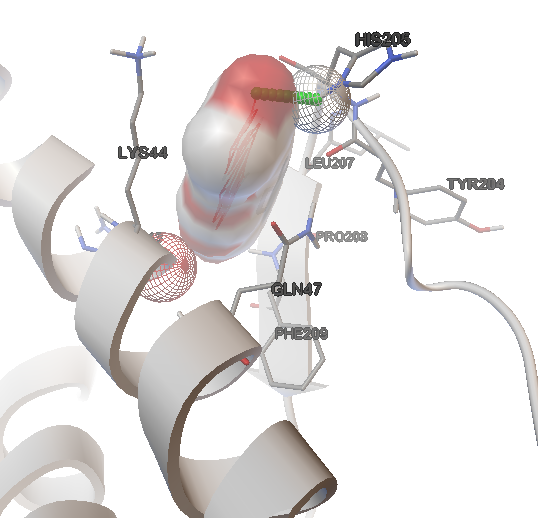

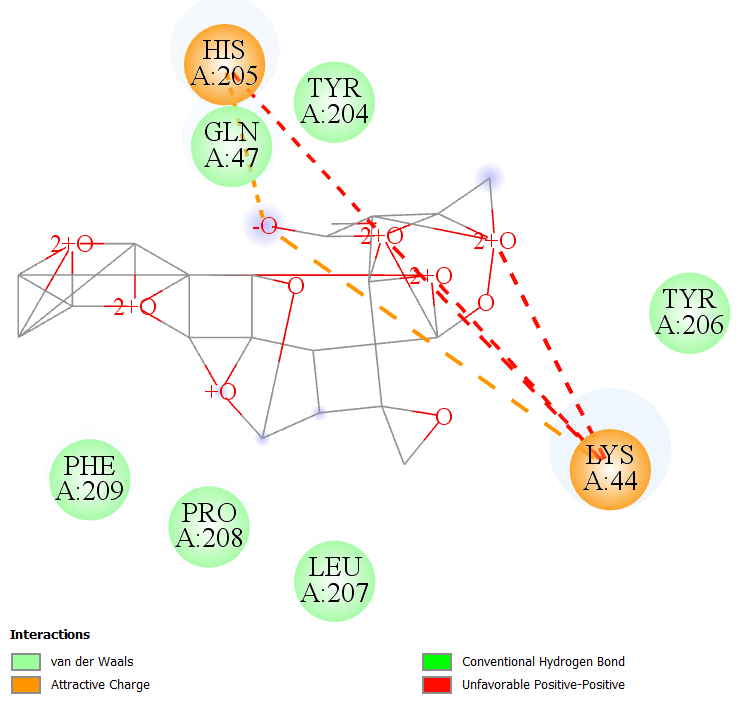

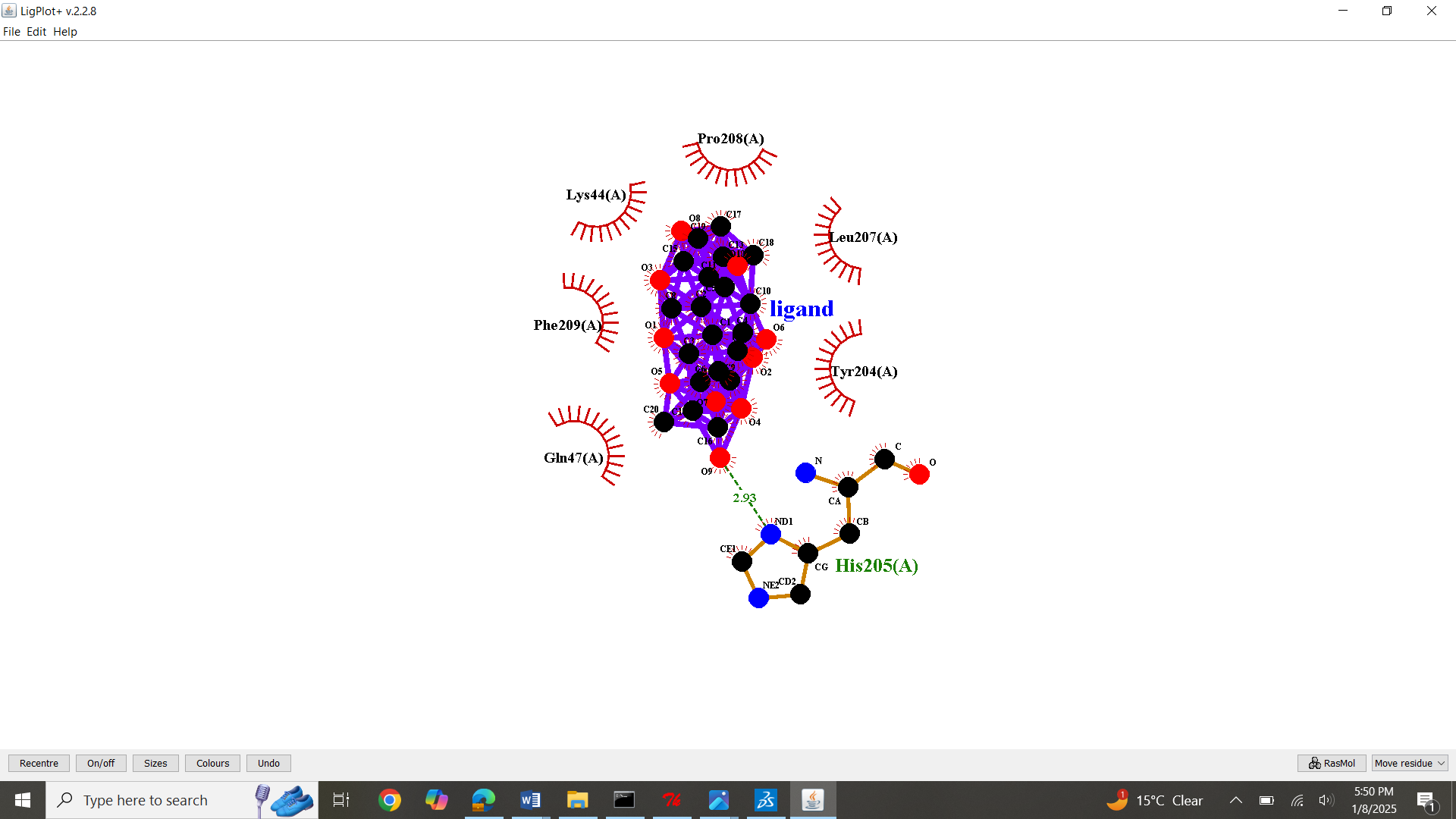


**S29: Molecular Docking, secondary structure interaction and ligplus analysis of Ginkolide interactions with Clustrin.**


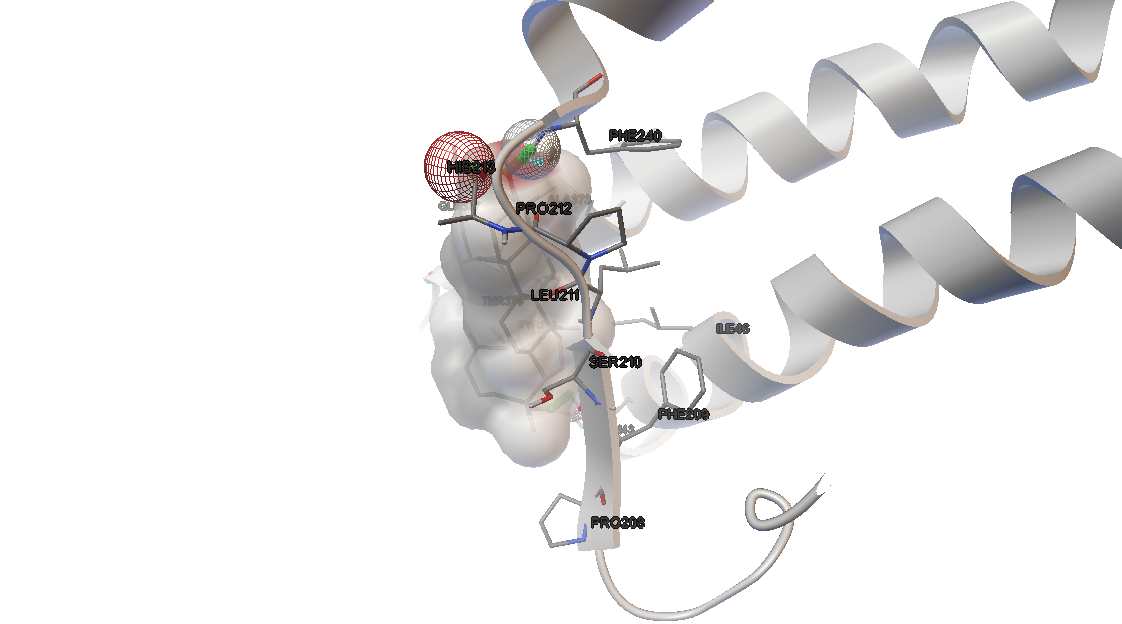

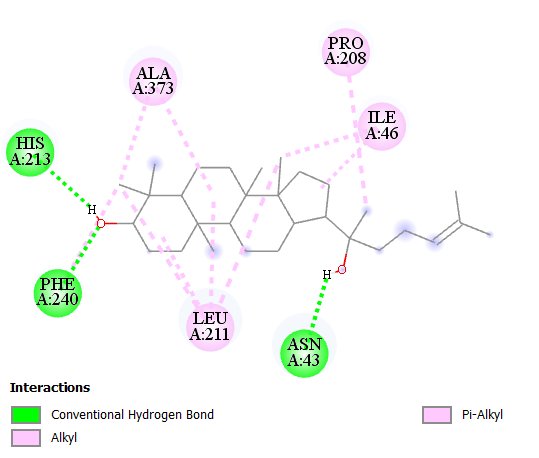

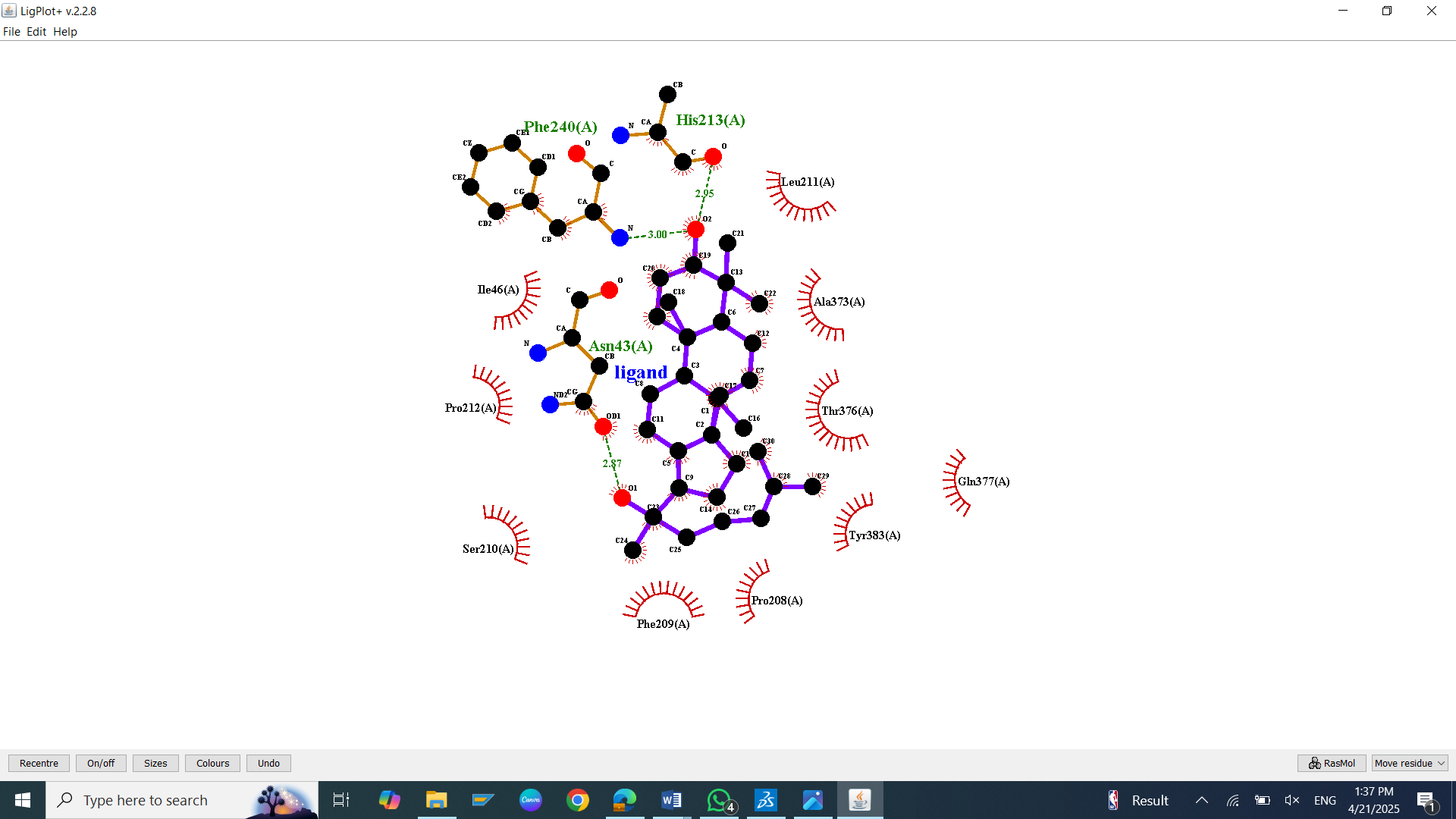


**S30: Molecular Docking, secondary structure interaction and ligplus analysis of Ginsenosides interactions with Clustrin.**


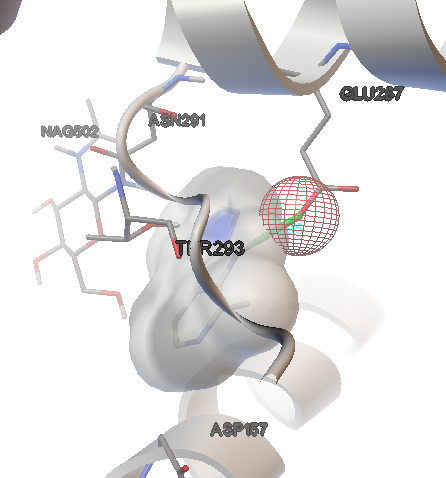

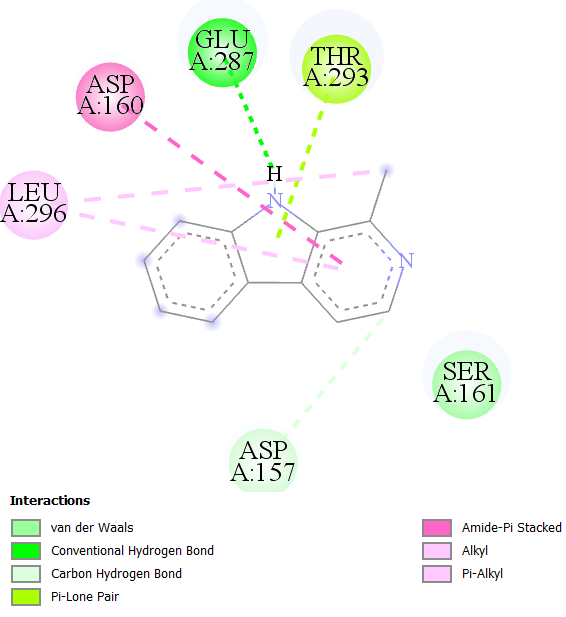

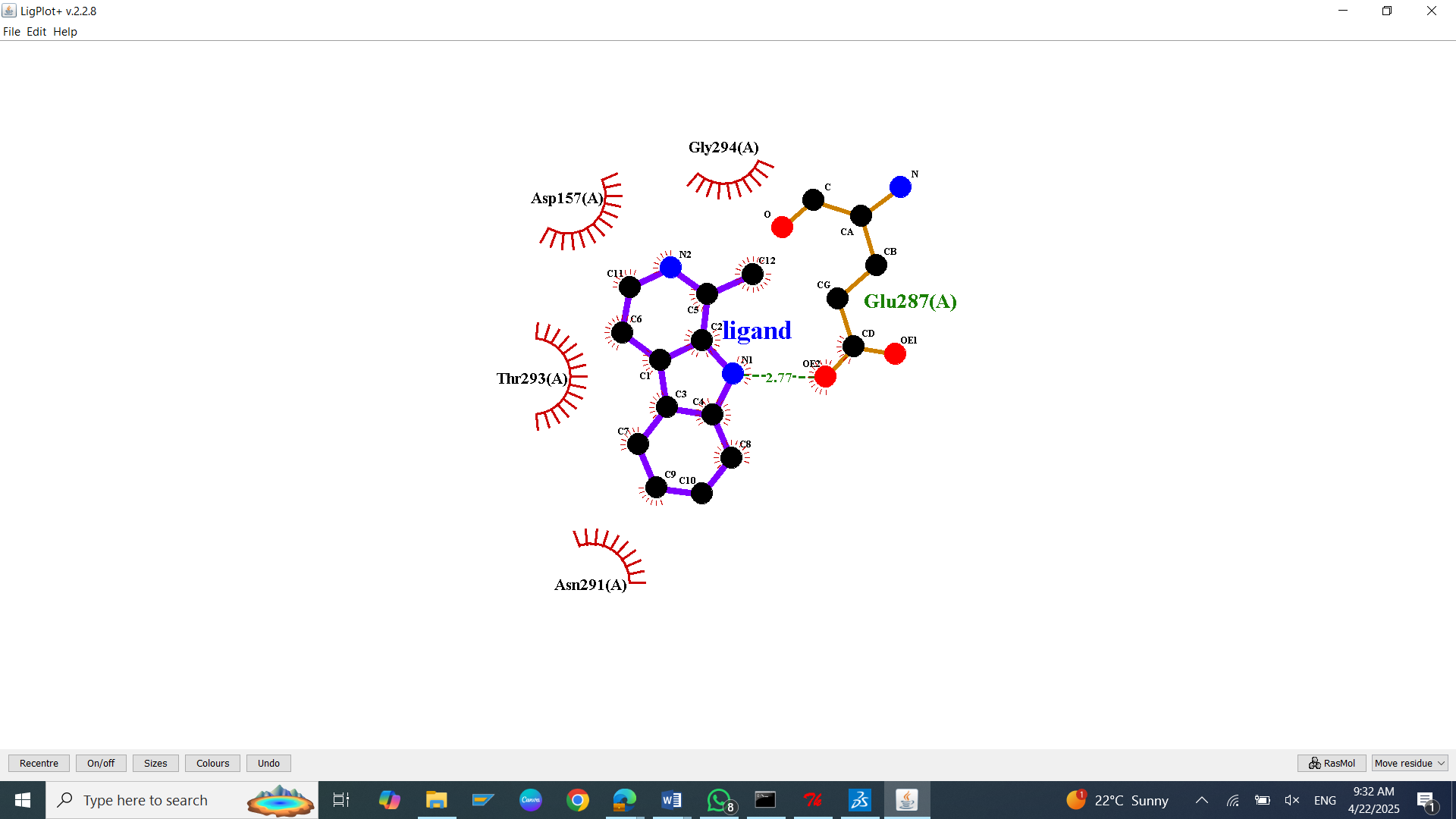


**S31: Molecular Docking, secondary structure interaction and ligplus analysis of Harman interactions with Clustrin.**


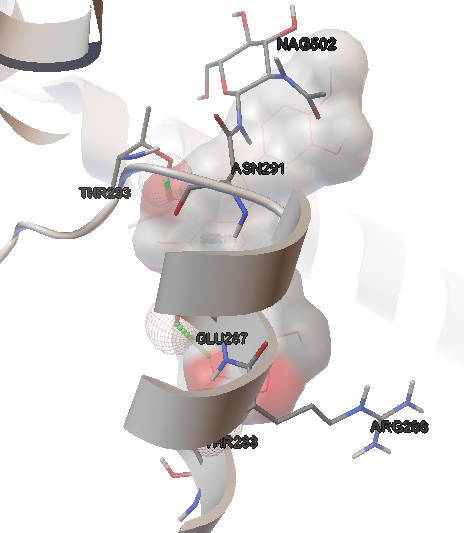

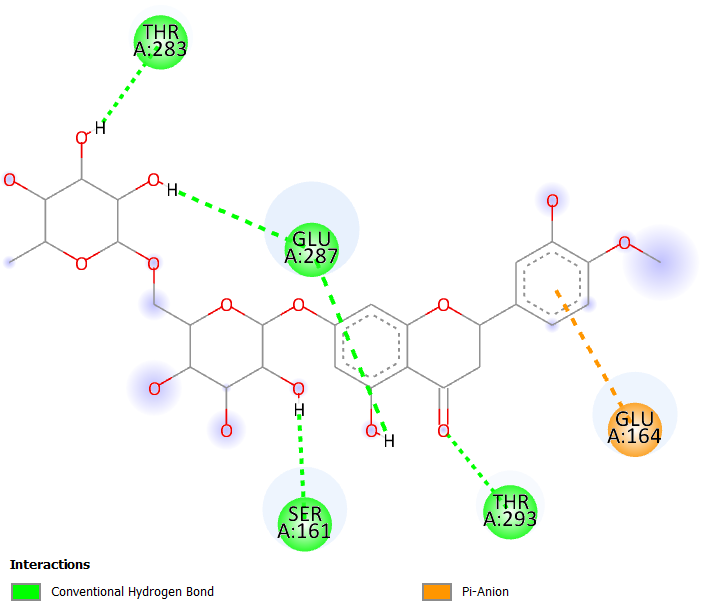

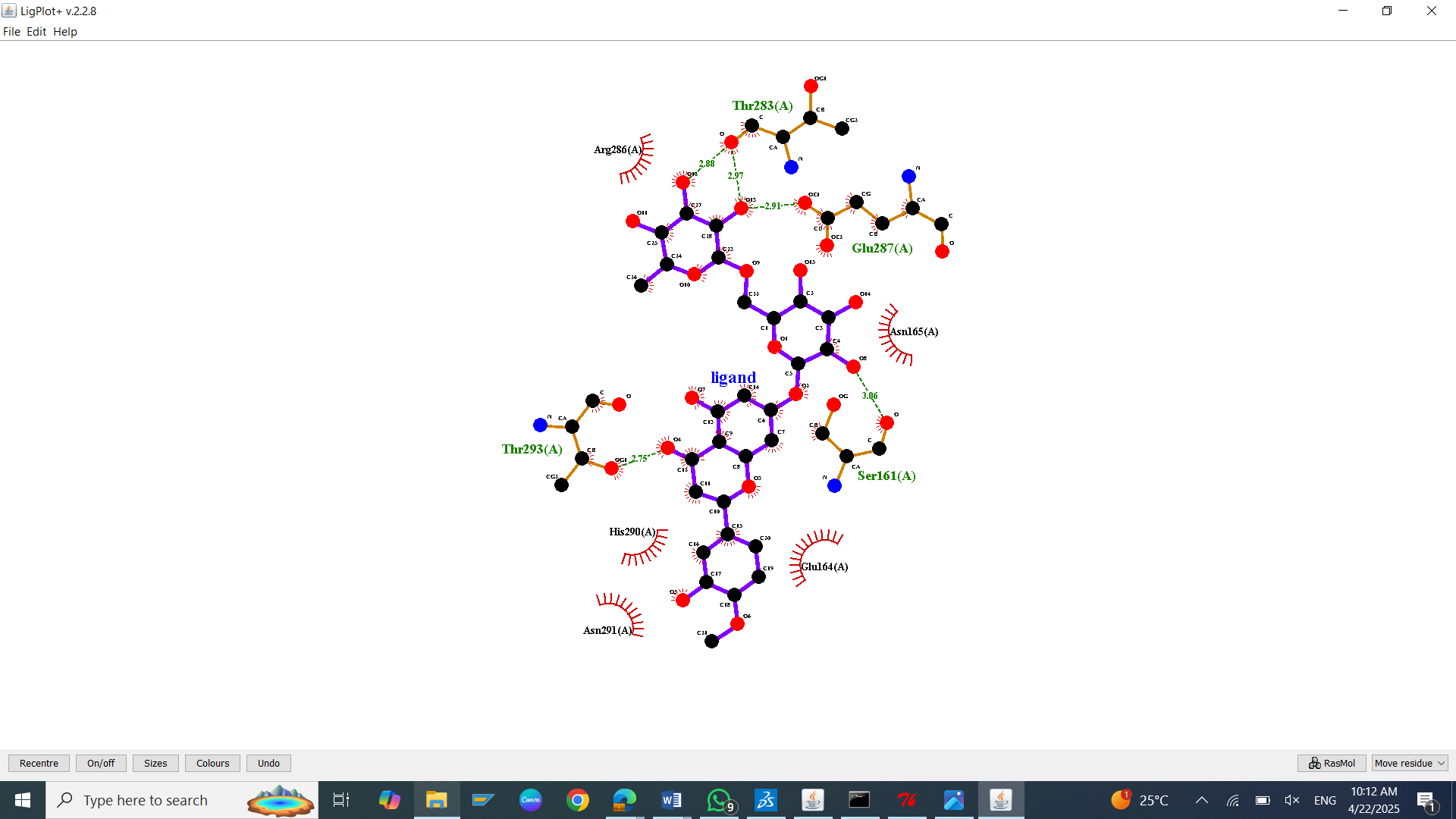


**S32: Molecular Docking, secondary structure interaction and ligplus analysis of Hesperidin interactions with Clustrin.**

**S33: Molecular Docking, secondary structure interaction and ligplus analysis of Resveratrol interactions with Clustrin.**

**S34: Molecular Docking, secondary structure interaction and ligplus analysis of Donepezil interactions with Clustrin.**

**S35: Molecular Docking, secondary structure interaction and ligplus analysis of Memantine interactions with Clustrin.**

**S36: Molecular Docking, secondary structure interaction and ligplus analysis of Rivastigmine interactions with Clustrin.**

**S37: Molecular Docking, secondary structure interaction and ligplus analysis of 4-tert-Amylphenol interactions with Amyloid peptide.**

**S38: Molecular Docking, secondary structure interaction and ligplus analysis of Allicin interactions with Amyloid peptide.**

**S39: Molecular Docking, secondary structure interaction and ligplus analysis of Apigenin interactions with Amyloid peptide.**

S**40: Molecular Docking, secondary structure interaction and ligplus analysis of Astragalosides interactions with Amyloid peptide.**

**S41: Molecular Docking, secondary structure interaction and ligplus analysis of Berberine interactions with Amyloid peptide.**

**S42: Molecular Docking, secondary structure interaction and ligplus analysis of Cannabidiol interactions with Amyloid peptide.**

**S43: Molecular Docking, secondary structure interaction and ligplus analysis of Curcumin interactions with Amyloid peptide.**

**S44: Molecular Docking, secondary structure interaction and ligplus analysis of Cyanidin interactions with Amyloid peptide.**

**S45: Molecular Docking, secondary structure interaction and ligplus analysis of Dronabinol interactions with Amyloid peptide.**

**S46: Molecular Docking, secondary structure interaction and ligplus analysis of Galantamine interactions with Amyloid peptide.**

**S47: Molecular Docking, secondary structure interaction and ligplus analysis of Gingkolide interactions with Amyloid peptide.**

**S48: Molecular Docking, secondary structure interaction and ligplus analysis of Ginsenosides interactions with Amyloid peptide.**

**S49: Molecular Docking, secondary structure interaction and ligplus analysis of Harman interactions with Amyloid peptide.**

**S50: Molecular Docking, secondary structure interaction and ligplus analysis of Hesperidin interactions with Amyloid peptide.**

**S51: Molecular Docking, secondary structure interaction and ligplus analysis of Reservetarol interactions with Amyloid peptide.**

**S52: Molecular Docking, secondary structure interaction and ligplus analysis of Donepezil interactions with Amyloid peptide.**

**S53: Molecular Docking, secondary structure interaction and ligplus analysis of Memantine** **interactions with Amyloid peptide.**

**S54: Molecular Docking, secondary structure interaction and ligplus analysis of Rivastigmine interactions with Amyloid peptide.**

**S55: Molecular Docking, secondary structure interaction and ligplus analysis of 4-tert-Amylphenol interactions with TAU.**

**S56: Molecular Docking, secondary structure interaction and ligplus analysis of Allicin interactions with TAU.**

**S57: Molecular Docking, secondary structure interaction and ligplus analysis of Apigenin interactions with TAU.**

**S58: Molecular Docking, secondary structure interaction and ligplus analysis of Astragalcocides interactions with TAU.**

**S59: Molecular Docking, secondary structure interaction and ligplus analysis of Berberine interactions with TAU.**

**S60: Molecular Docking, secondary structure interaction and ligplus analysis of Cannabidiol interactions with TAU.**

**S61: Molecular Docking, secondary structure interaction and ligplus analysis of Curcumin interactions with TAU.**

**S62: Molecular Docking, secondary structure interaction and ligplus analysis of Cyanidin interactions with TAU.**

**S63: Molecular Docking, secondary structure interaction and ligplus analysis of Dronabinol interactions with TAU.**

**S64: Molecular Docking, secondary structure interaction and ligplus analysis of Galantamine interactions with TAU.**

**S65: Molecular Docking, secondary structure interaction and ligplus analysis of Gingkolide interactions with TAU.**

**S66: Molecular Docking, secondary structure interaction and ligplus analysis of Ginsenosides interactions with TAU.**

**S67: Molecular Docking, secondary structure interaction and ligplus analysis of Harman interactions with TAU.**

**S68: Molecular Docking, secondary structure interaction and ligplus analysis of Hesperidin interactions with TAU.**

**S69: Molecular Docking, secondary structure interaction and ligplus analysis of Resveratrol interactions with TAU.**

**S70: Molecular Docking, secondary structure interaction and ligplus analysis of Donepezil interactions with TAU.**

**S71: Molecular Docking, secondary structure interaction and ligplus analysis of Memantine interactions with TAU.**

**S72: Molecular Docking, secondary structure interaction and ligplus analysis of Rivastigmine interactions with TAU.**
